# Biofilm Disruption from within: Light-Activated Molecular Drill-Functionalized Polymersomes Bridge the Gap between Membrane Damage and Quorum Sensing-Mediated Cell Death

**DOI:** 10.1021/acsbiomaterials.4c01177

**Published:** 2024-08-23

**Authors:** Bela B. Berking, Sjoerd J. Rijpkema, Bai H. E. Zhang, Arbaaz Sait, Helene Amatdjais-Groenen, Daniela A. Wilson

**Affiliations:** Systems Chemistry Department, Institute for Molecules and Materials, Radboud University, Nijmegen 6500 HC, The Netherlands

**Keywords:** biofilm, nanodrill, polymersome, quorum sensing, membrane damage

## Abstract

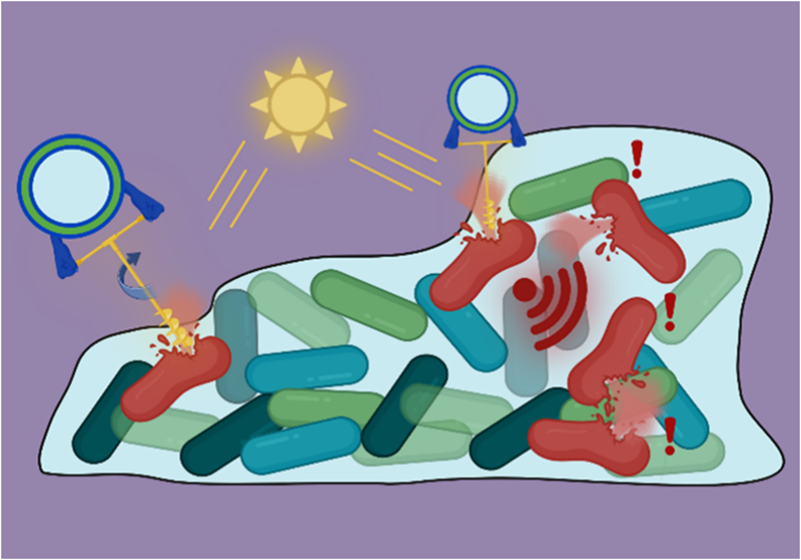

Bacterial biofilms represent an escalating global health concern with the proliferation of drug resistance and hospital-acquired infections annually. Numerous strategies are under exploration to combat biofilms and preempt the development of antibacterial resistance. Among these, mechanical disruption of biofilms and enclosed bacteria presents a promising avenue, aiming to induce membrane permeabilization and consequent lethal damage. Herein, we introduce a hemithioindigo (HTI) motor activated by visible light, capable of disrupting sessile bacteria when integrated into a polymeric vesicle carrier. Under visible light, bacteria exhibited a notable outer membrane permeability, reduced membrane fluidity, and diminished viability following mechanical drilling. Moreover, various genetic responses pertaining to the cell envelope were examined via qRT-PCR, alongside the activation of a self-lysis mechanism associated with phage stress, which was coupled with increases in quorum sensing, demonstrating a potential self-lysis cascade from within. The multifaceted mechanisms of action, coupled with the energy efficiency of mechanical damage, underscore the potential of this system in addressing the challenges posed by pathogenic biofilms.

## 1 Introduction

The emergence of antibiotic-resistant bacterial biofilms represents a mounting health crisis, contributing to elevated mortality rates among hospitalized patients, substantial extensions in hospitalization periods, and associated medical expenses. Up to 80% of surgical site infections are estimated to be linked to bacterial biofilms, with persister cells frequently responsible for recurrent infections and the failure of postantibiotic therapy completion.^1,2^

Biofilms are characterized as a sessile group of bacteria embedded in a self-produced extracellular matrix (ECM).^3^ While bacterial biofilms make up the majority of biomass in the microbial world, in a medical setting, these coordinated masses can be disruptive to tissue healing and significantly impair the ability of healthy cells to function normally.^4,5^ A common example of a hospital-acquired pathogen is presented by *Pseudomonas aeruginosa*, an opportunistic Gram-negative rod-shaped bacterium that often colonizes medical devices, open wounds and tissues, and more specifically the lungs of cystic fibrosis patients.^6,7^ Biofilm growth on top of these tissues can lead to altered production of cytokines, reduced immune response, and, in response, inflammation of the tissue, at the expense of the suffering patient. Not only does this impose a burden on patients, hindering the healing process, but it also carries significant economic ramifications, with global estimates reaching up to $281 billion in 2019.^8^

A big hurdle in combating bacterial biofilms is the protection given to sessile bacteria by the ECM.^9,10^ Consisting of a multitude of biomolecules, including deoxyribonucleic acid (DNA), proteins, lipids, and polysaccharides, as well as active enzymes, this protective shield is able to absorb common antibiotics and shelter bacteria from the host immune system.^11−13^ The design of novel antimicrobials faces the first challenge when tasked with overcoming this barrier, either by active penetration or diffusion into the deeper layers of biofilms to ensure sufficient eradication of the complete biofilm. Bacteria have adapted to these attacks with an arsenal of mechanisms such as fortifying the biofilm with increased amounts of eDNA through self-lysis or phage-mediated cell lysis.^14,15^ These coordinated responses have been found to lie under partial control of the bacterial communication system, the quorum sensing apparatus, steering these lysis responses in a cascade-like manner through parts of the biofilm.^16^

Molecular rotors have been gaining great attention recently due to their promising applications in the medical field. By utilizing intelligent systems with built-in stimuli responsiveness, targets can be manipulated at the flick of a light switch, allowing for the penetration of lipid bilayers.^17^ Molecular rotors have been used to effectively kill cancer cells, fungi, and bacteria, with the main target being penetration of the membrane, leading to increases in reactive oxygen species ROS, leakage of intracellular contents, and overall loss of integrity.^18−20^ Especially as antimicrobial treatments, these rotors have excellent chances of impacting the global challenge of growing resistance owing to their diverse modes of action. A variety of induced stresses were detected when bacteria were exposed to molecular rotors, showcasing the difficulty that these systems pose to bacteria for gaining resistance, a mechanism that was proven to be not possible for multiple strains tested.^20,21^ Examples in the literature have been utilizing these novel antimicrobials in a “pure” state, not conjugated to any carrier, which allows for the potential advantage of easier cell wall penetration and distribution. However, this approach restricts these rotors from carrying payloads. Conversely, multiple rotors connected to a particle provide further advantages as they could enhance further biofilm disruption due to stronger synergetic forces over the membrane in addition to sensing capabilities. Incorporating these rotors into polymersomes offers a solution, enabling the attachment of various cargoes to the vesicle through integrated functional handles within or on the polymer membrane or taking advantage of the inner aqueous compartment provided by polymersomes.^22^ By decorating a polymersome with a molecular rotor, we could deliver a payload deep inside the biofilm, overcoming the defensive barrier provided by the ECM.

**Figure 1:**
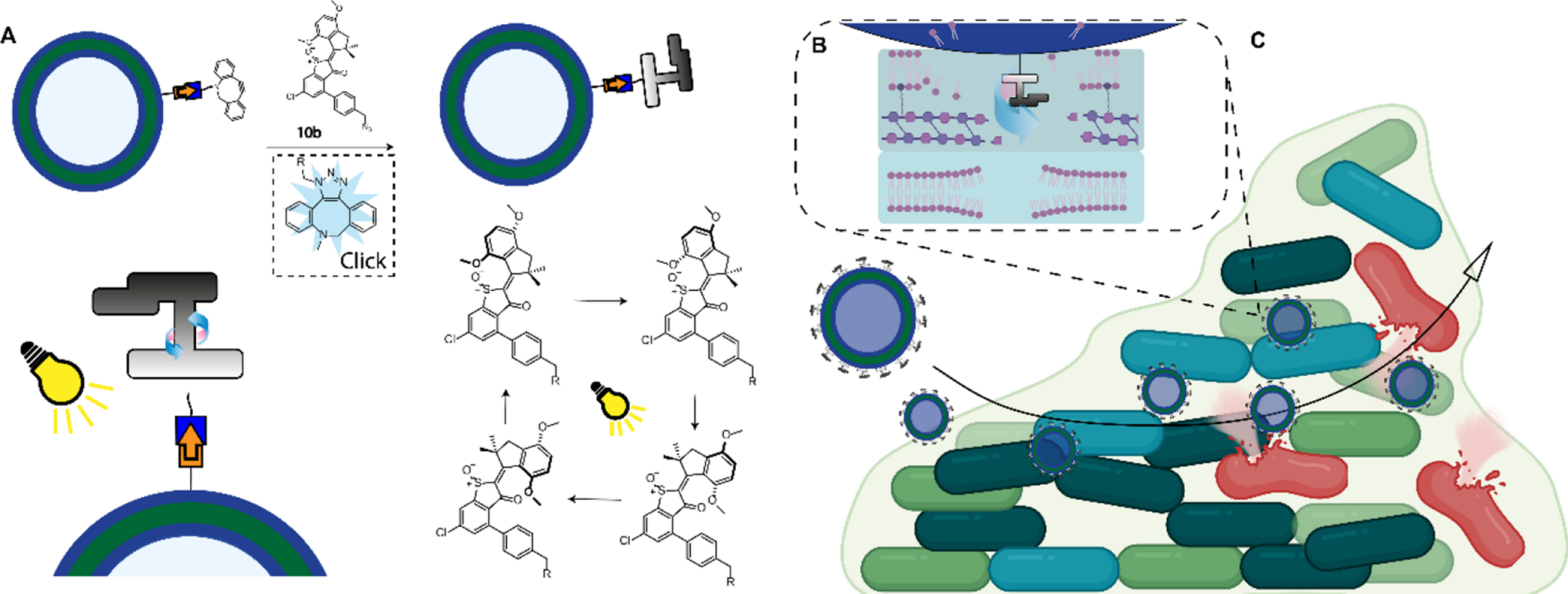
(A) System design and conjugation step to fabricate hemithioindigo (HTI) rotor-decorated polymersomes with the steps of HTI rotation. (B) Proposed mechanism of membrane-HTI interaction. Outer membrane, peptidoglycan layer, as well as the inner membrane of the bacterial cell all become permeabilized and damaged. (C) Biofilm infiltration and the killing of bacteria via membrane damage. Accumulation of HTI-polymersomes in the center of the biofilm results in effective killing of the bacteria, even without direct contact to the cell via a quorum sensing cascade. Figure created with Biorender.com.

The group of Dube has developed several hemithioindigo (HTI)-based rotors.^23−26^ Based on their work, we have designed an HTI rotor that can be coupled to polymersomes. HTI motor 10 possesses an azide handle, which allows for copper-free click binding to polymersomes with dibenzylcyclooctyne (DBCO) handles. A single binding spot was chosen over two to not allow for accidental coupling to two different polymersomes. This enables the polymersome to function as a nanodrill, a capability previously demonstrated with other molecular motors^27^ but not yet explored with nanostructures. The integration of this capability with a cargo-carrying polymersome, to the best of our knowledge, has not been attempted before. We fabricated HTI-functionalized polymersomes from supramolecular assemblies based on poly(ethylene glycol)-*b*-polystyrene (PEG-*b*-PS) diblock copolymer (Figure 1) and exposed the biofilms to this system.

Upon light activation, HTI-Polymersomes efficiently infiltrated biofilms and eradicated a majority of bacterial cells within them. Furthermore, hydrophobic payloads encapsulated within the polymersomes, as demonstrated by Nile Red, exhibited notable accumulation within the biofilm, suggesting the potential for targeted delivery. Detection of loss of membrane fluidity and increased outer membrane permeability of bacteria corroborated the main target of the HTI-polymersome antimicrobial effect. We set out to investigate potential molecular responses and were able to determine three genetic responses after exposure: the upregulation of the outer membrane homeostasis pathway *mlaA* (maintenance of outer membrane lipid asymmetry) which upholds the outer membranes lipid asymmetry and flexibility,^28^ and the upregulation of *PA3691*, which is linked to a hypothetical protein responsible for a peptidoglycan repair response.^29^ As a last effort to fortify the biofilm, bacteria activate a self-lysis mechanism via the endolysin *lys*, which causes the excretion of eDNA.^30^ We hypothesize that the mechanical damage mimics a phage-like attack and activates a self-lysis signal via quorum sensing, as we detected increases in the quorum sensing apparatus, to fortify the biofilm. We believe that the mechanism goes beyond just opening up the membrane but rather “tricks” the whole biofilm into a self-lysis response as it would in nature.

## 2 Experimental Section

### 2.1 General Preparation of Polymersomes with DBCO Handles

Modified from a previous report,^31^ a general procedure is described: MeO-PEG-*b*-PS (8 mg) and DBCO-PEG-*b*-PS (2 mg) were dissolved in a mixture of THF and 1,4-dioxane (1 mL, 4:1 *v/v*) in a 15 mL capped vial with a magnetic stir bar. After dissolving the solution for 0.5 h at 21 °C, a syringe pump equipped with a syringe and a needle was used to deliver ultrapure water at a rate of 1 mL/h for 0.5 h via a rubber septum while vigorously stirring the mixture (900 rpm). The appearance of a cloudy suspension indicated the formation of the polymersomes. Upon finishing the water addition was finished, 8 mL of ultrapure water was added to the suspension, which ensured a rapid quenching of the PS domain within the bilayer of the polymersomes. The polymersomes were spun down via centrifuge (10 min, 10.000 rpm) and washed with ultrapure water a total of three times.

### 2.2 Click Reaction on DBCO-PEG-*b*-PS Polymersomes

DBCO-PEG-*b*-PS polymersomes (10 mg, 20% functionalized) were diluted with MeOH (2 mL). An excess of 10b in MeOH (100 μL,1 mg/mL) was added to the solution, and the mixture was stirred for 16 h in the dark. The polymersomes were washed (3× with ultrapure water) by centrifugation to remove the methanol and excess of 10b, and were resuspended in ultrapure water.

### 2.3 Particle Characterization

Size and surface charge were determined using a DLS Zetasizer blue instrument (Malvern). Samples were diluted to 0.01 mg/mL polymer concentration and measured. Transmission Electron Microscopy (TEM) was conducted on a JEOL JEM-1400 FLASH running at 120 kV. Samples were airdried overnight on a copper grid.

### 2.4 dSTORM Microscopy

To show HTI drill binding on the surface, dSTORM super-resolution microscopy was conducted on the ONI nanoimager. Polymersomes with 20% DBCO handle availability were incubated with AlexaFluor 647-N_3_ for 16 h for binding. Channel Slides carrying an Avidin surface coating were provided by ONI. Polymersomes were incubated with an anti-PEG antibody. Polymersomes were then gently flowed through the channel to be captured on the surface. Unbound particles were washed away before the dSTORM buffer was added to the channel. Samples were imaged using a 647 laser utilizing 3000 frames acquisition.

### 2.5 Biofilm Experiments

The overnight culture was inoculated in 6 mL of Brain Heart Infusion (BHI) by adding 5 μL of *P. aeruginosa* ATCC 10145, 50% glycerol stock, and incubated overnight at 37 °C. The next day, the resulting culture was diluted to an OD of 0.001 and was seeded into wells of 96- or 12-well plates for biofilms to grow. The plate was incubated at 37 °C for 2 h after which the medium was refreshed to remove the bulk of the planktonic cells. The plate was then returned to the incubator for overnight incubation to allow for biofilm formation. After allowing biofilm formation for 24 h, the medium was carefully aspirated and the wells were washed 3 times with 1× phosphate-buffered saline (PBS). Polymersomes were added at 0.1 mg/mL to the wells, resulting in the addition of 10 μL to 100 μL of PBS in the wells. 10 μL of ultrapure water was added to the control wells to mimick the slight dilution of the PBS buffer. Half of the plate was wrapped in aluminum foil to cover the wells (no light exposure control), while the other half was exposed to a 450 nm laser for 2 h to activate the polymersomes. The well plate was placed on ice during the laser exposure to slow down bacterial metabolism and prevent further biofilm production as well as prevent heating effects. After 2 h, the wells were carefully aspirated, and experiments were conducted as described below.

### 2.6 Confocal Microscopy

Imaging was conducted using an SP8x AOBS-WLL confocal laser scanning microscope. Syto9 and propidium iodide were used to create a suitable working solution: for Syto9, a final concentration of *c* = 11.1 nM, and for propidium iodide, a final concentration of *c* = 66.6 nM in PBS (150 mM NaCl, 100 mM NaPO4 mM, pH 7.4). Biofilms were stained for 10 min and washed three times with PBS (150 mM NaCl, 100 mM NaHPO_4_, pH 7.4). Lasers were set at λex = 470 nm, λem = 500–520 nm and λex = 560 nm, λem = 620–670 nm. Images were later analyzed with Imaris.

### 2.7 Period Acid-Schiff Assay

Based on a known method, the PAS assay was carried out using the solutions collected after treatment of the biofilms to quantify the exopolysaccharides released due to the drilling. 175 μL of 0.5% periodic acid (in 5% acetic acid) was added to wells of a 96-well plate. 25 μL of the collected samples were then added to the wells and left to incubate for 30 min. 100 μL of Schiff’s reagent was then added to the wells and left to incubate overnight. Absorbance measurements were then taken at 544 nm with a Spark multimode microplate reader (Tecan).

### 2.8 Bradford Assay

To assess if an increased amount of protein could be detected in the supernatant after HTI exposure, which could be a result of ECM damage, a Bradford assay was conducted. 40 μL of Bradford reagent was added to 20 μL of the collected samples in a 96-well plate. Ultrapure water was then added to make up a final volume of 200 μL. Absorbance measurements were taken at 595 nm with a Spark multimode microplate reader (Tecan).

### 2.9 NPN Assay

To assess outer membrane permeability, the fluorescent probe *n*-phenyl-1-Naphthylamine (NPN) was used. Biofilms were left to form over 24 h incubation time after which various treatments were conducted as described before. Wells were gently washed three times with PBS before the addition of NPN solution to a final concentration of 12 μM. Parallel to NPN, Syto9 was added to normalize wells in terms of biomass to acquire a more accurate comparison. Wells were measured at λex= 320 nm, λem= 420 for NPN and λex= 450 nm, λem= 550 for Syto9

### 2.10 Membrane Fluidity Assay

Membrane fluidity assay was performed as previously described.^32^ Biofilms were left to form over 24 h incubation time after which various treatments were conducted as described before. Wells were gently washed three times with 1x PBS, Using a cell scraper all material was scraped off the wells and centrifuged for 10 min at 6000 rcf to collect cells. The supernatant was removed, and the cells were fixed in 0.37% Glutaraldehyde for 30 min. Bacteria were then frozen in liquid nitrogen for 5 min, thawed, and resuspended in 0.6 mM DPH solution and measured at λex= 350 nm, λem= 425.

### 2.11 RNA Extraction

Following irradiation, the total RNA was extracted from the biofilms using the RNeasy Kit of QIAGEN. The media was removed from the biofilm and washed once with 1× PBS. Before scraping the biofilm of the wells, 1 volume of 1× PBS and 2 volumes of RNA protect bacteria reagent were added to each well. The samples were then scraped and transferred into bead-beating tubes, and the cells were lysed by 0.1 mm Zirconia/silica beads in the BeadBug 6 bead homogenizer for 3 cycles of 30 s on and off at 4000 rpm Following the lysis, the lysate was transferred to an RNeasy Mini Spin Column and placed in 2 mL collection tubes. The columns were centrifuged at ≥8000×*g* for 15 s, and the flow-through was discarded and the collection tube was reused. This step was repeated until all lysate was processed. Then, to wash the spin column membrane, 700 μL of Buffer RW1 was added and centrifuged at ≥8000×*g* for 15 s. The flow-through was discarded, and the columns were placed in new collection tubes. Subsequently, 500 μL of Buffer RPE was added and centrifuged at ≥8000x g for 15 s. This step was repeated once more with 500 μL of Buffer RPE and centrifuged at ≥8000×*g* for 2 min to ensure the removal of ethanol. The spin columns were then transferred to new 1.5 mL collection tubes, and 30–50 μL of Rnase-free water was added directly to the membrane. The columns were then centrifuged at ≥8000×*g* for 1 min to elute the RNA.

The concentration and purity of the RNA were determined by a Nanodrop 1000 spectrophotometer. The absorbance ratio A260/280 served as a measure of protein contamination, while the ratio A260/230 served as a measure of contamination of polysaccharides, phenols, and salts. Additionally, Agarose Gel electrophoresis was conducted.

### 2.12 cDNA Synthesis

For the cDNA synthesis, the RNA was treated with DNase to remove genomic DNA. This was done using DNase I Amplification grade by Invitrogen. The following was added to RNase-free PCR strips on ice: 500 ng of total RNA, 1 μL of 10X DNase I Reaction Buffer, 1 μL of DNase I Amplification grade (1U/μL), and DEPC-treated water to 10 μL. Then, the PCR strips were incubated for 15 min at room temperature. Then, 1 μL of 25 mM EDTA was added to inactivate the DNase I. Lastly, the RNA samples were incubated for 10 min at 65 °C. cDNA was synthesized using SuperScript II Reverse Transcriptase by Invitrogen. To each DNase-treated RNA 9 μL of the following mix were added: 1 μL of random primers (250 ng/μL), 1 μL of 10 mM dNTP’s, 4 μL of 5X first Strand Buffer, 1 μL of 0.1 M DTT, 1 μL of RNaseOUT (10 U/μL), 0.5 μL of Superscript II (200 U/μL), and 0.5 μL of DEPC-treated water. Mixed and incubated for 10 min at 25 °C, followed by another incubation of 50 min at 42 °C, and the reaction was inactivated by heating it for 15 min at 70 °C. After the cDNA synthesis, the cDNA was purified using the QIAquick PCR Purification Kit of QIAGEN. This was done according to the manufacturer’s instructions. Following purification, the cDNA concentration was measured with a Qubit 4 Fluorometer using the 1X high-sensitivity dsDNA assay.

### 2.13 qRT-PCR

qRT-PCR was performed using an iQ SYBR Green Supermix by Bio-Rad. Each reaction contains 10 μL of iQ SYBR Green Supermix, 2 μL of (10 μM) forward primer, 2 μL of (10 μM) reverse primer, 1 ng of cDNA, and DEPC-treated water to a final volume of 20 μL. The primers that were used are listed in Table 1.

**Table 1:** Primer Design for All Genes of Interest^a^

gene of interest	forward sequence (5′→3′)	reverse sequence (5′→3′)
*mlaA*	GCATCAACCGTCCCATCTTC	GGTTGTTGGCCAGGTTCTTC
*PA3691*	TCGAGATGAAGTCCGCTCAG	GCATCCTTCACGGCTTTCTG
*lys*	ATGAAACTGACCGAGCAGCA	TGTGTCGATCTCCCTCTCGT
*recA*	GAAGTTCTACGCCTCGGTCC	GTTCTTCACCACCTTGACGC
*gyrA*	ATGGAGGTGATCCGTGAGGA	TTCTTCACGGTACCGAAGGC
*rhlR*	CTCCTCGGAAATGGTGGTCT	TTCTGGGTCAGCAACTCGAT

a Primers were designed using Primer3 and verified via Blast.

The qRT-PCR was performed in a Bio-Rad C1000 Touch Thermal Cycler using the following protocol and the primers in Table 1: 95 °C for 30 s, 95 °C for 30 s, 60 °C for 10 s, and 72 °C for 20 s (repeat 39X), followed by a melt-curve analysis from 58 to 95 °C at a 0.5 °C/cycle melt rate. The relative gene expression was calculated using the 2-ΔΔCT method, where all Ct values were normalized to the housekeeping genes gyrA and recA

### 2.14 Cytotoxicity Studies

HEK293T, Chinese Hamster Ovarian (CHO) cells were cultured in Dulbecco’s modified Eagle’s medium (DMEM) supplemented with 10% fetal bovine serum (FBS). After cells reached a confluence of around 50%, they were rinsed with 1x PBS three times and detached with 4 mL of Trypsin for 3 min. Trypsin was quenched by adding 8 mL of DMEM. The cells were transferred to a 15 mL falcon and spun down for 5 min at 0.3 rcf. The supernatant was discarded, and cells were seeded with DMEM complete medium in a 96-well plate at a density of 4.0–4.5 × 10^4^ cells/mL and incubated for 24 h at 37 °C with 5% CO2. Afterward, HTI-polymersomes and DBCO-polymersomes were added to the wells and subjected to light exposure or not. 10 μL of CCK8 (Sigma-Aldrich) was added to the wells and incubated for 3 h, and the absorbance was measured at 450 nm to determine viability. Experiments were carried out in replicates of 8.

## 3 Results and Discussion

### 3.1 Synthesis of the Drill

**Scheme 1:**
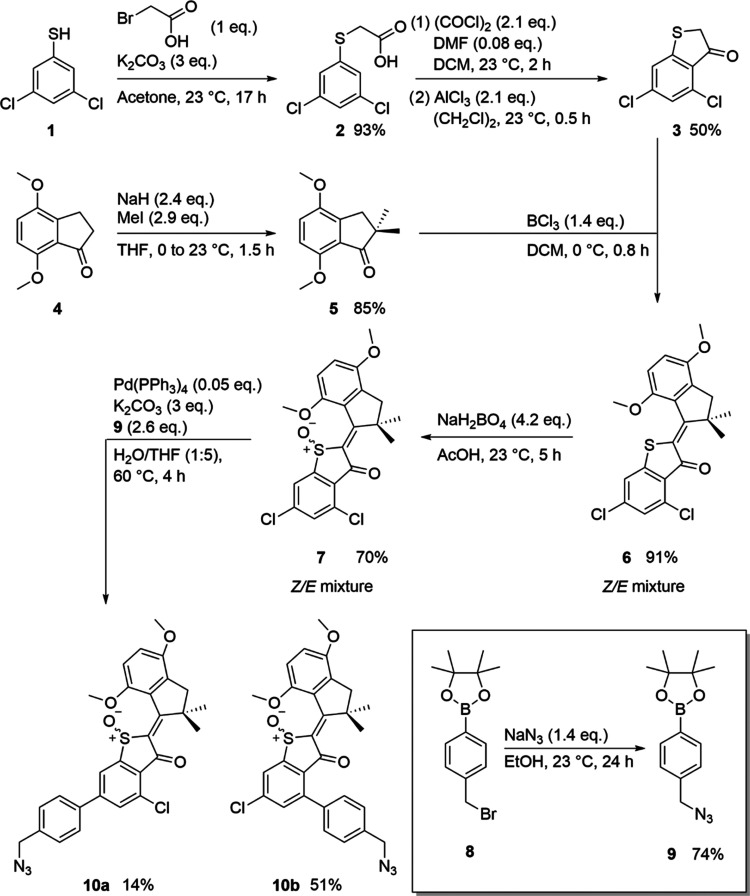
Synthesis of the Final Products (**10**) Starting from Commercially Available 3,5-Dichlorobenzenethiol (**1**) and 4,7-Dimethoxy-2,3-dihydro-1H-inden-1-on (**4**) Only the thermodynamically most stable Z isomers are shown for clarity. Only the Z isomer of 7 was used to make 10.

The synthesis of the azide functionalized HTI motor **10** (Scheme 1) was based on the previous work from the group of Dube.^23,26^

Starting from the commercially available 3,5-dichlorobenzenethiol **1**, we first converted it to the thioacetic acid **2** via a nucleophilic substitution reaction in 93% yield. Then, the acid was converted into the acyl chloride via Vilsmeier reagent,^33^ after which without purification an intramolecular Friedel–Crafts acylation was performed to make the dichlorinated benzothiophenone **3**. Although conversion looked promising with only a small impurity, after purification via column chromatography, only a 50% yield was obtained. In subsequent repetitions, we found that it was also possible to continue with the crude oil in order to minimize the loss of compound. Commercially available 4,7-dimethoxy-1-indanone **4** was methylated twice in the 2-position using sodium hydride and methyl iodide to give indanone **5** in 85% yield in a fast reaction after purification. The condensation of **3** and **5** using boron trichloride as a Lewis acid then yielded dichlorinated HTI **6** as a mixture of *Z* and *E* isomers in 91% combined yield and was completed in less than an hour. Purification of **6** via column chromatography proved to be challenging as it was found to be unstable on silica. Similarly, Nuclear magnetic resonance (NMR) spectra of the motors were taken in CD_2_Cl_2_ instead of the more common CDCl_3_, as we found that the isomers were unstable in CDCl_3_. However, oxidizing the crude product of **6** with sodium perborate also yielded HTI motor **7** as a mixture of *Z* and *E* isomers without complications. Purification of **7** via column chromatography was possible without stability issues, making it possible to separate the isomers into 31% *Z* and 39% *E*. Because the *Z* isomer is thermodynamically more stable, we used this isomer for the next reaction step. Subsequently, we had to connect a moiety that could attach to the polymersome, for which we used an azide handle. For this, we had to make the azide boronic acid pinacol ester **9** from the commercially available **8** by reaction with sodium azide. This reaction was performed as reported in the literature with a similar yield of 74%.^34^ A subsequent Suzuki cross-coupling between motor **7** and boronic acid pinacol ester **9** gave the final functionalized HTI motor **10a** in 14% and **10b** in 53% yield, respectively, after purification in only the *Z* confirmation. Addition at both chloride positions was possible, but a clear preference for **10b** was found. We hypothesize this position is more reactive due to closer proximity to the ketone. We also obtained the double addition product in trace amounts. **10b** was then used to bind to the polymersome.

### 3.2 Self-Assembly of HTI-Decorated Polymersomes

**Figure 2:**
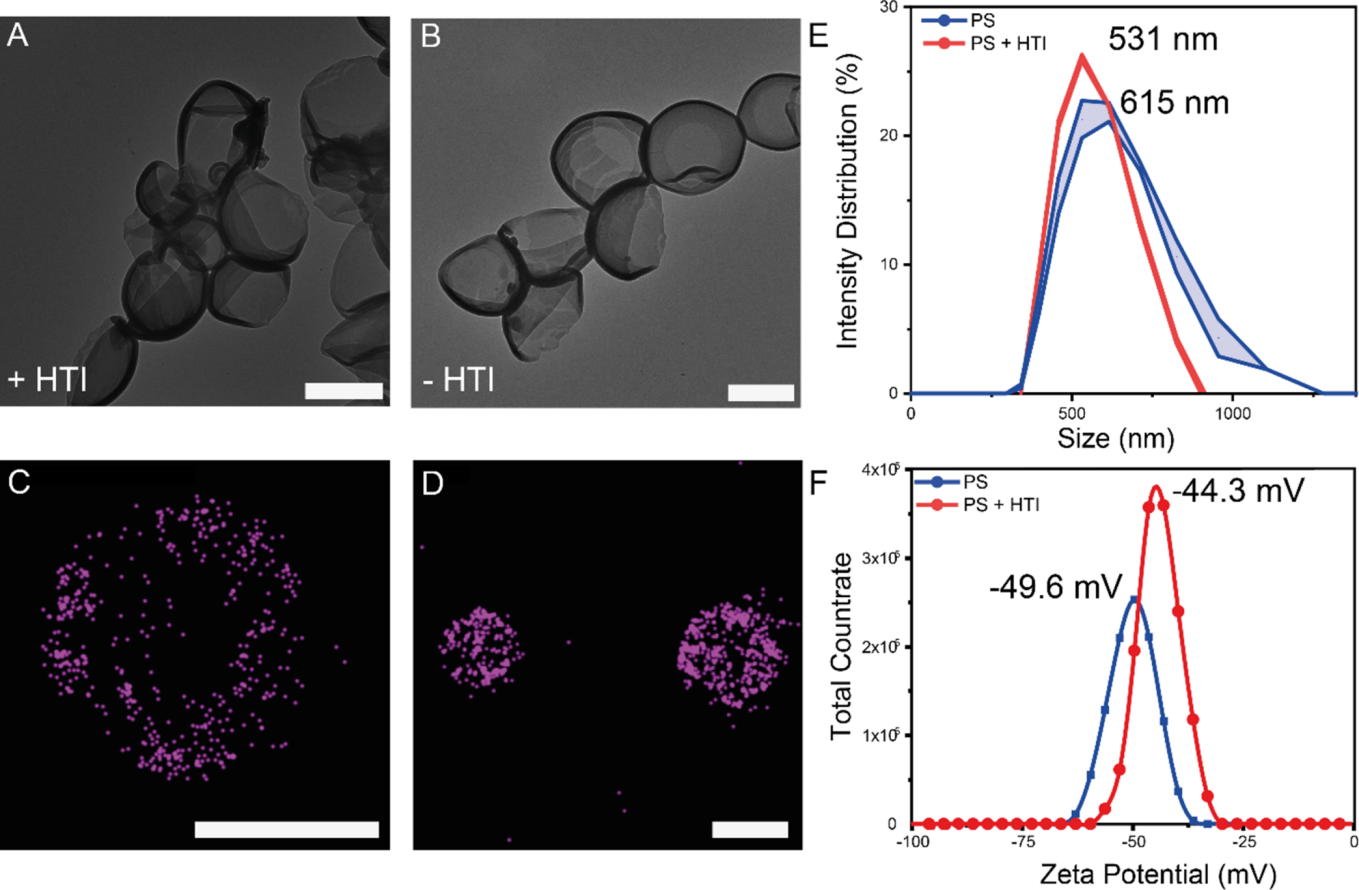
Particle characterization: TEM of polymersomes with HTI (A) and without HTI (B). Scale bars represent 500 nm. dStorm image of polymersome with AF647 (C, D). Scale bar represents 200 nm. Size analysis (E) of polymersomes with (red) and without HTI (blue) in nanometers and surface charge (F) in mV determined by DLS.

Polymersomes were self-assembled by slow addition of water over a solution of amphiphilic polymer PEG-*b*-PS in THF-Dioxane (4:1) using a process we have previously reported.^22,35,36^ Afterward, **10b** was attached to the polymersomes via a click reaction between the azide and DBCO functional groups overnight. The resulting polymersomes were washed with MeOH 3× to remove excess **10b**, after which they were resuspended in ultrapure water. Removal of MeOH and excess unbound **10b** was verified by NMR (Figures S5 and S6). We have previously shown the availability and reactivity of the DBCO handles.^22^ To confirm the binding of **10b** to the polymersomes, we redissolved part of the sample in CD_2_Cl_2_ and investigated the sample via diffusion NMR. This showed comparable diffusion speeds for both the PEG-*b*-PS signals and motor signals, whereas uncoupled **10b** has an approximately 10× faster diffusion speed compared to the polymer, indicating successful coupling (Figures S1–S3). Additionally, 1H-13C HMBC spectra before and after binding show a shift of the DBCO alkyne peaks, going from 85 to 137 ppm after binding (Figure S7). After fabrication of HTI-decorated polymersomes, particles were characterized using TEM, dSTORM, and DLS (Figure 2). TEM images revealed no morphological changes to the polymersomes before and after binding (Figure 2A,B). To demonstrate the homogeneity of the HTI drill on the surface, the available handles were cross-linked to a fluorophore in the same way the drill is bound, imitating the binding procedure. Utilizing dSTORM microscopy, a surface mapping of the polymersomes reveals evenly distributed handles (Figure 2C,D). This is of importance to understand later mechanisms by excluding the effects of an asymmetric particle. Particle characterization revealed polymersome formation with an average size of around 500 nm for particles with and without a drill (Figure 2E). Zeta potential showed an increase toward a more positive surface charge, which can be linked to the presence of the positively charged HTI drill on the surface (Figure 2F). Change of the surface charge is a good indication of successful binding and has been previously used to determine surface modification.^35^ Furthermore, binding was verified with diffusion NMR and HTI-decorated polymersomes were disassembled to detect the presence of HTI molecules (Figure S2).

### 3.3 Evaluation of Antibiofilm Properties

**Figure 3:**
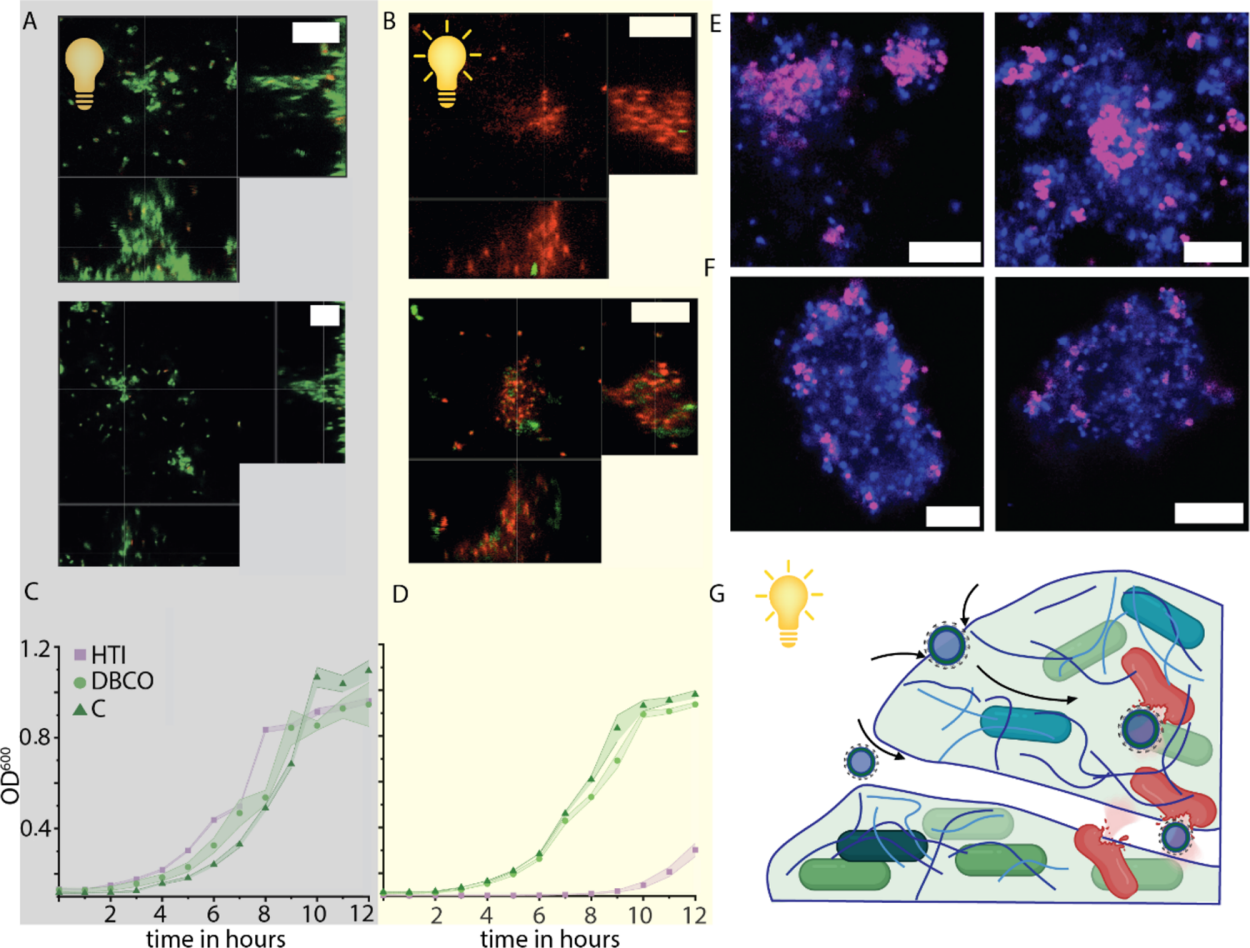
Confocal microscopy of biofilms exposed to HTI-decorated polymersomes without (A) and with (B) subsequent light activation. Scale bars are 10 μm. Recovery of bacteria after exposure to HTI in the dark (C) and under light (D) determined by OD_600_ measurements over 12 h, *n* = 6–9. HTI-polymersomes (magenta) infiltrate biofilms (blue) (E), while DBCO-polymersomes remain on the outer edge of biofilms (F). Scale bars represent 10 μm. Potential mechanisms of biofilm infiltration (G) include pushing through ECM or passing through pores. Figure created with Biorender.com.

Initial experiments aimed at demonstrating the ability of the Drill system to remove biofilm mass via mechanical damage, so release of extracellular matrix material was expected. Polysaccharides and protein concentrations were determined in the supernatant after exposure. Results indicated no significant increase in ECM components in the supernatant, which implies that the activated HTI drill did not manage to shred off the ECM (Figure S4). By visualizing 24 hr old biofilms formed by *P. aeruginosa* after exposure, it became evident that the structure of the biofilms remained intact, as can be seen in orthogonal slices of exposed biofilms (Figure 3A,B). Structures of the ECM still reach heights of more than 10 μM. Although structurally intact, a significant amount of bacteria in the biofilm suffered membrane damage, visible by Live/Dead staining. In a rescue experiment, we resuspended the bacteria previously exposed to the HTI-polymersomes and mock treatments in media and let them grow in a forced planktonic state for 12 h (Figure 3C,D). All treatments were able to recover and reached close to stationary phase after 12 h, while the HTI-polymersome-treated biofilms under light exposure did not recover (Figure 3D). This raises the question of polymersome infiltration into the biofilm and subsequent bacterial damage. At around 500 nm, HTI-decorated polymersomes are capable of infiltrating water channels and pores which have been known to stretch through the biofilm, although at this size only a small percentage would be expected to diffuse into the biofilms.^37^ When visualizing biofilms after exposure to fluorescent HTI-polymersomes (magenta), accumulation can be seen in the center areas of the biofilm (blue) (Figure 3E). DBCO-decorated control polymersomes can be seen remaining on the outer area of the biofilms with just a few individual particles having diffused into the biofilms (Figure 3F). We hypothesize that upon contact, the HTI-polymersomes can push themselves forward into the biofilm via rotational force of the HTI drill, damaging bacteria in the process (Figure 3G).

The delivery of cargo into the biofilm is an advantageous property that we envisioned for this system to have. Using a nanoparticle over only the molecular rotor allows for more modifications and also stronger mechanical forces over the bacterial membrane. In Figure 3E, the Nile Red used to label polymersomes is embedded inside the layers during self-assembly, a method used previously to deliver f.e. doxorubicin.^38^ Utilizing a different hydrophobic antibiotic, the same effect could be achieved. Due to the different compartments polymersomes offer, hydrophilic drug entrapments would also be possible.^39^

**Figure 4:**
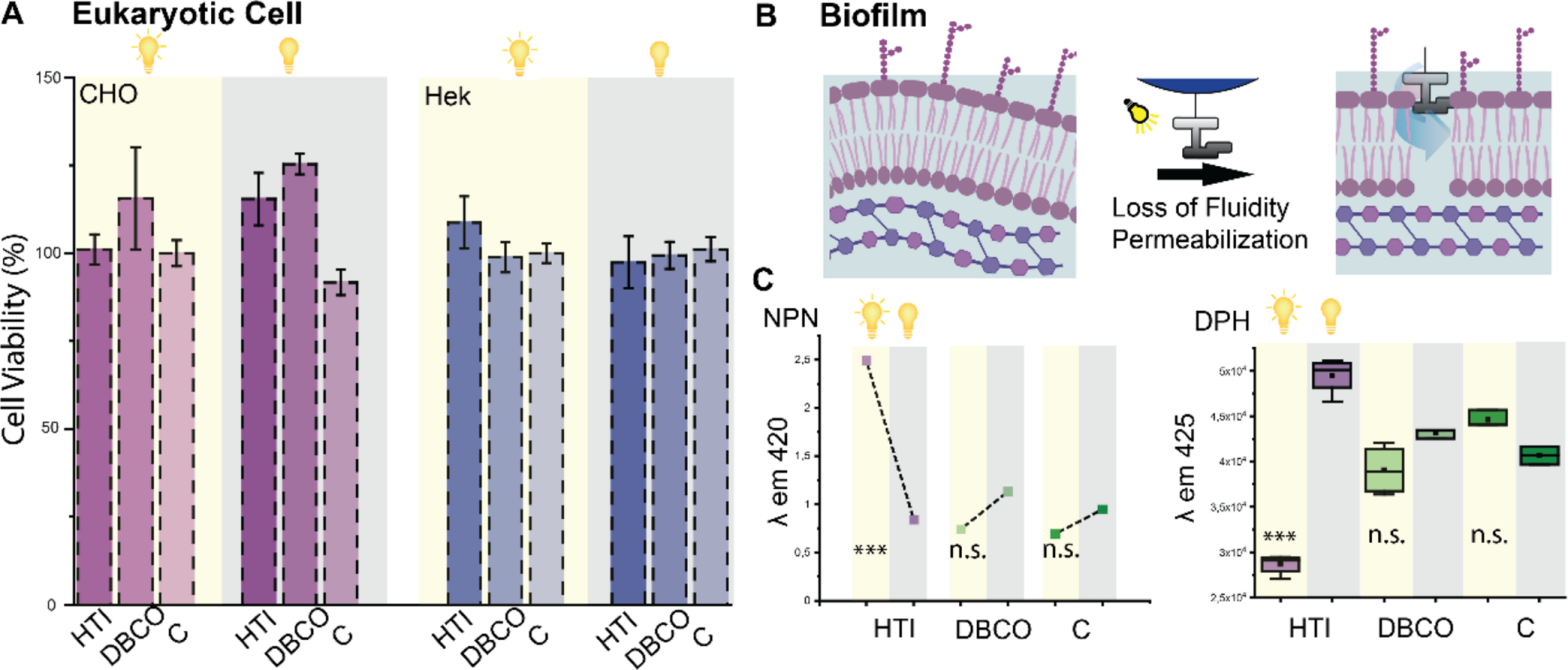
Effects of HTI-polymersomes on eukaryotic cells vs biofilms. Cytotoxicity studies of HTI-polymersomes, DBCO-polymersomes, and controls under light and dark using CHO and Hek cells. *N* = 8, significance tested with two-sided *t* test, yet no significant decrease in viability was detected. (B) Outer membrane permeability and membrane fluidity determined by NPN and DPH. Schematic overview of changed membrane properties after exposure to HTI-polymersomes, verified experimentally (C) with NPN and DPH probes. Experiments were conducted in 4–8 biological replicates, *p* ≤ 0.01. Figure created with Biorender.com.

To assess potential harmful effects on eukaryotic cells, we conducted cytotoxicity studies using Chinese Hamster Ovarian (CHO) cells and Hek cells. Remarkably we did not detect any decrease in cell viability when exposing cells to HTI-polymersomes under light (Figure 4A). Additionally, the light control experiments show that light itself is not strong enough to damage healthy cells and consequentially healthy tissue.

### 3.4 Assessment of Bacterial Membrane Damage

It has been previously shown that one of the main modes of action by molecular rotors is disruption and damage of the membrane (Figure 4B).^20,21^ Subjecting biofilms to membrane-sensitive probes such as *N*-phenyl-naphthylamine (NPN) and 1,6-diphenyl-1,3,5-hexatriene (DPH) allowed one to better quantify the damage inflicted and show the light-activated mechanism of membrane damage even when bacteria are fortified in their biofilms (Figure 4C).

NPN assays have been widely utilized to showcase an increase in the permeability of the outer membrane (OM).^40^ When biofilms are exposed to HTI-decorated polymersomes yet kept from light exposure, the OM remains at similar levels of permeability as the controls (Figure 4B). Activating the HTI drill results in a significant increase in the permeability of the OM, indicating membrane damage by insertion and opening of the OM. This results in the loss of membrane integrity as well as fluidity, which was demonstrated by the probe DPH. Light-activated damage decreased the fluidity of the membrane, turning it more rigid, an effect of the membrane damage and lipid bilayer destruction (Figure 4B).^41^

### 3.5 Genetic Analysis

**Figure 5:**
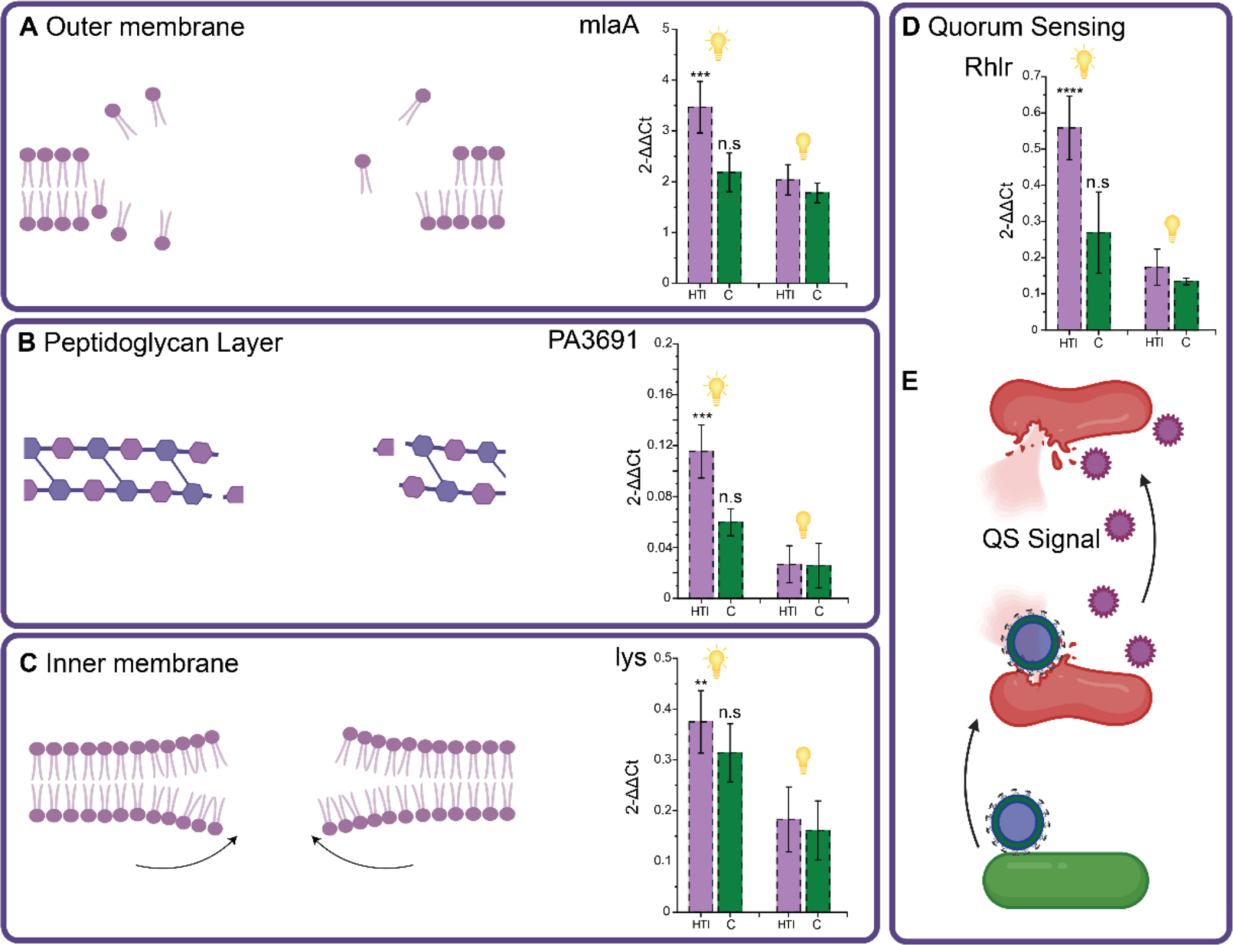
Analysis of membrane marker genes *mlaA*, *PA3691*, and *lys* for outer membrane (A), peptidoglycan layer (B), and inner membrane (C) as well as *Rhlr* to monitor quorum sensing (QS) (D). Genes were analyzed via qRT-PCR in three biological replicates, *p* ≤ 0.01. (E) Proposed mechanism of quorum sensing-mediated lysis after exposure to HTI-polymersomes. The initial membrane damage triggers quorum sensing response, which affects bacteria even without HTI-polymersome contact. Figure created with Biorender.com.

We hypothesized that bacteria would activate various molecular responses to handle the damage inflicted, protect the biofilm, and adapt to further stressors of such a kind, as they would in a natural setting. Analysis of various genes was achieved by monitoring transcript levels after exposure (Figure 5).

Responsible for maintaining the outer membrane homeostasis is the Mla System. MlaA has been shown to increase outer membrane integrity when being activated, which has been studied in the case of membrane-targeting antibiotics and by deletion studies.^42,43^ When exposed to the HTI-polymersomes under light activation, this apparatus is significantly upregulated, as a response to outer membrane damage and loss of fluidity, as previously demonstrated (Figure 4B), an attempt to reorganize the lipid composition by trafficking phospholipids to increase membrane resilience via lipid asymmetry.^28,44^ Following the outer membrane, the HTI-polymersomes encounter the peptidoglycan layer, where the hypothetical protein PA3691 has been attributed the role of peptidoglycan repair by upregulation under membrane damage,^29,45^ which can be seen in Figure 5B under light activation. *P. aeruginosa* has been shown to undergo a self-lysis mechanism to fortify the biofilm using DNA under external stressors such as strong light and mechanical damage.^30,46^ This type of response has also been observed when biofilms experience a phage attack, where the outer layer will self-lyse to protect the inner layer, a mechanism believed to be sensing-mediated and induced by the surrounding damaged cells as a cascade-like reaction.^16^ Considering the combination this system uses, utilizing light as fuel to exert mechanical damage, self-lysis is a viable explanation for the observed structurally intact biofilms with nonviable bacteria in them. To monitor this mechanism, the responsible lysing protein Lys (PA0629) was chosen to be analyzed via qRT-PCR. Since we have already demonstrated damage to the outer membrane, the way is paved for this endolysin to open the membrane to release material to fortify the biofilm. Transcript levels can be seen increasing for the treated biofilms and control biofilms, although HTI-exposed biofilms have higher transcript levels, which is due to the combination of light and mechanical damage.

Looking back at the intact biofilms previously visualized with confocal microscopy (Figure 3B), a reasonable explanation for this is partial lysis of the bacteria in order to increase the stability of the biofilm and protect it from further attacks. In the event of bacteriophage attacks, sensing-mediated lysis has been shown to occur in *Vibrio cholerae* biofilms as a means of biofilm formation or protection. We set out to investigate a correlation between the quorum sensing of biofilms after treatment as a potential trigger to cause cascade-like self-lysis, even when bacteria were not directly in contact with an HTI-polymersome. The *Rhlr* gene, a main protagonist in the quorum sensing apparatus, was significantly upregulated after treatment (Figure 5D), thus demonstrating a potential link between the previously described self-lysis after HTI-polymersome exposure. We believe that the internalization of HTI-polymersomes shown to occur (Figure 3E) causes a phage-like attack that, unlike in nature, happens in the center and the outer layer of the biofilm as opposed to just from the outer layer. This attack triggers a QS-mediated self-lysis of bacteria, resulting in the death of the biofilm from within. Bacteria that have come into contact with HTI-polymersomes undergo membrane damage which causes an increase in produced quorum sensing molecules. This signal cascade then causes bacteria to self-lyse even when they have not come directly into contact with HTI-polymersomes (Figure 5E). This highly conserved lysis response could mean a certain specificity toward bacteria in which the HTI-polymersomes act, which is further supported by cytotoxicity studies demonstrating no adverse effects on Chinese hamster Ovarian cells and Hek cells (Figure 4A). These findings pave the way for further exploring HTI-polymersomes in more complex environments such as in vivo studies to efficiently eradicate pathogenic biofilms.

## 4 Conclusions

In conclusion, this study has demonstrated the development of HTI-decorated polymersomes as an effective strategy for combating bacterial biofilms. Through various experiments, we confirmed the successful infiltration of polymersomes into biofilms, leading to bacterial sensing and killing via a sensing drill and kill mechanism. Our mechanistic investigations revealed that membrane permeabilization and loss of fluidity are key modes of action, while a self-lysis “death” signal propagated via quorum sensing allows for further damage of the biofilm killing the bacteria from within the biofilm. While the HTI-decorated polymersomes did not break apart the ECM of the biofilm, this in fact turned into an advantage, as we anticipate it would prevent a strong immune response for in vivo applications. Genetic analysis further illustrated the diverse responses bacteria employ to survive stress induced by light-activated HTI-polymersomes, including outer membrane and peptidoglycan repair. These findings highlight the multitude of potential targets for antibacterial interventions, which is crucial for overcoming the current issues of antibiotic resistance. By leveraging a fuel-free system, we anticipate that light-activated rotors will emerge as a sustainable alternative to existing antibiofilm approaches. The ability to turn the bacterial signal/sensing apparatus against the entire biofilm makes this a unique approach to killing biofilms from within.

## Supporting Information Available

Additional experimental details; IR MS and NMR spectra (PDF)
