# Supplementary material for: Biofilm Disruption from within: Light-Activated Molecular Drill-Functionalized Polymersomes Bridge the Gap between Membrane Damage and Quorum Sensing-Mediated Cell Death
Source: ACS Biomater Sci Eng. 2024 Aug 23;10(9):5881–91. doi: 10.1021/acsbiomaterials.4c01177 (PMC11388143; doi:10.1021/acsbiomaterials.4c01177)
Supplement: Supplementary file 1 — ab4c01177_si_001.pdf [file ab4c01177_si_001.pdf]

Supplementary Information to

Biofilm Disruption from Within: Light activated Molecular Drill functionalized Polymersomes bridge the gap between Membrane Damage and Quorum Sensing mediated Cell Death

Bela B. Berking‡, Sjoerd J. Rijpkema‡, Bai H. E. Zhang, Arbaaz Sait, Helene Amatdjais-Groenen, Daniela A. Wilson*.

Experimental

Materials

All PEG polymers with different functional end groups were obtained from AV Chemistry. All other reagents were obtained from commercial sources and were used without purification unless otherwise stated. Solvents were dried by passing over activated alumina columns in a MBraun MB SPS800 under a nitrogen atmosphere and stored under argon. Reactions were carried without the need for an inert atmosphere unless stated otherwise, in which case the reaction was performed under a dry atmosphere of argon. Standard syringe techniques were applied for the transfer of dry solvents and air- or moisture sensitive reagents. Styrene was passed over alumina to remove the inhibitor 4-tert-butylcatechol. Ultrapure water was obtained from a QPOD MilliQ system.

Instrumentation

Nuclear Magnetic Resonance (NMR) characterization was carried out on a Bruker AVANCE HD nanobay console with a 9.4 T Ascend magnet (400 MHz) and a Bruker AVANCE III console with a 11.7 T UltraShield Plus magnet (500 MHz) equipped with a Bruker Prodigy cryoprobe, in chloroform ($CDCl_3$). NMR spectra were recorded at 298 K unless otherwise specified. Chemical shifts are given in parts per million (ppm) with respect to tetramethylsilane (TMS, δ 0.00 ppm) as internal standard for ^1H NMR. Coupling constants are reported as J-values in Hz. Peak assignment is based on 2D gDQCOSY, ^1H-^{13}C gHSQCED, and ^1H-^{13}C gHMBC spectra. Side group and end of chain signals separated from the bulk polymer ^1H signal are only reported when observed with clear s/n ratio and no overlap with polymer peaks, and may be (in)visible on other NMR spectrometers or with different concentrations. Gel permeation chromatography (GPC) equipped with PL gel 5 μm mixed D column calibrated for polystyrene (580 to 377,400 g/mol) was carried out on a Shimadzu instrument with THF as eluent using differential refractive index and UV (254 nm) detectors. Transmission electron microscopy (TEM) was carried out on a JEOL TEM 1400 equipped with CCD camera at 60 kV. Samples were prepared by drop casting 5 μL of appropriately diluted samples on a carbon coated Cu grid (200 mesh) and dried overnight at room temperature. Cryogenic TEM was carried out with a JEOL TEM 2100. Malvern Zetasizer nano S was used for dynamic light scattering (DLS) measurements equipped with He-Ne laser of wavelength 633 nm. Fluorescence was measured on a Tecan Spark 200. All images analysis was carried out using ImageJ, available in a public domain http://fiji.sc/.[51] 300 W xenon light source was purchased from Asahi Spectra, Japan (MAX-303) with a wavelength range of 385-740 nm.

Experimental Procedures

Synthesis of molecular motor. *2-((3,5-dichlorophenyl)thio)acetic acid) (2)*. To a solution of bromoacetic acid (2.50 g, 14.0 mmol, 1 eq.) and K_2CO_3 (5.85 g, 42.3 mmol, 3 eq.) in acetone at 0 °C was added **1** (2.15 g, 15.5 mmol, 1.1 eq.). Then the mixture was warmed to 23 °C and stirred for 16 h. The reaction mixture was made acidic (pH 1) by addition of an aqueous solution of HCl (70 mL, 2 M). The crude product was extracted with AcOEt (3 x 100 mL) and the combined organic layers were dried over Na_2SO_4 and evaporated in *vacuo*. The crude product was purified by recrystallization from heptane to yield **2** (3.06 g, 93%). **^{1}H NMR** (400 MHz, $CDCl_3$) δ 7.28 (d, J = 1.8 Hz, 2H, 2-C*H*), 7.26 (d, J = 1.8 Hz, 1H, 4-C*H*), 3.74 (s, 2H, 6-CH_2) ppm. **^{13}C NMR** (101 MHz, $CDCl_3$) δ 174.8 (1-*C*), 138.2 (5-*C*), 135.5 (3-*C*), 127.2 (2-*C*), 127.1 (4-*C*), 35.7 (6-*C*) ppm. ***R*f** 0.51 (AcOEt/Acetone, 4:1 v/v). **m.p.** 101.8 °C. **IR (FTIR, solid)** 3674, 2971, 2901, 1690, 1557, 1394, 1278, 1201, 1066, 922, 891, 870, 847, 827, 793, 659 cm^{-1}. **HR-MS (ESI-MS)** [$C_8H_5Cl_2O_2S^-$] calc. 234.93873, found 234.93947.

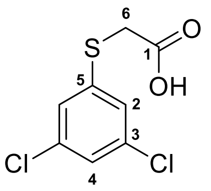

4,6-dichlorobenzo[b]thiophen-3(2H)-one (3). To a solution at 0 °C of **2** (2.93 g, 12.35 mmol, 1 eq.) in dry CH_2Cl_2 (40 mL) and under Argon atmosphere was added oxalyl chloride (2.29 mL, 3.32 g, 26.16 mmol, 2.1 eq.) and DMF (80 µL, 75 mg, 1.00 mmol, 0.08 eq.). Then the mixture was warmed to 23 °C and stirred for 2 h. After removing the volatiles *in vacuo*, the residue was dissolved in 1,2-dichloroethane (40 mL) and cooled to 0 °C. $AlCl_3$ (3.40 g, 25.5 mmol, 2.1 eq.) was added slowly under an Argon atmosphere and the reaction mixture was allowed to warm up to 23 °C. After stirring for 30 min, a saturated solution of $NaHCO_3$ (140 mL) was added and then was extracted with CH_2Cl_2 (3 x 200 mL). The combined organic layers were dried with Na_2SO_4 and evaporated *in vacuo*. The crude product was purified by column chromatography from Heptane/AcOEt (98:2 v/v) to yield the beige/pink solid **3** (1.36 g, 50%). **^{1}H NMR** (400

MHz, CD_2Cl_2) δ 7.35 (d, *J* = 1.7 Hz, 1H, 6-C*H*), 7.19 (d, *J* = 1.7 Hz, 1H, 4-C*H*), 3.86 (s, 2H, 8-CH_2) ppm. **^{13}C NMR** (101 MHz, CD_2Cl_2) δ 196.1 (1-*C*), 160.7 (7-*C*), 142.2 (5-*C*), 135.7 (3-*C*), 127.5 (4-*C*), 125.7 (2-*C*), 123.6 (6-*C*), 40.8 (8-*C*) ppm. ***R*f** 0.20 (AcOEt/Hept, 1:20 v/v). **m.p.** 152.5 °C. **IR (FTIR, solid)** 3077, 2984, 2928, 1696, 1681, 1571, 1547, 1386, 1368, 1295, 1224, 1209, 1172, 1081, 1019, 876, 840, 824, 785, 708, 647, 596, 579 cm^{-1}. **HR-MS (GC-EI-MS)** [$C_9H_6Cl_2OS^+$] calc. 217.93599, found 217.93594.

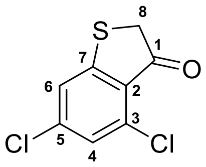

*4,7-dimethoxy-2,2-dimethyl-2,3-dihydro-1H-inden-1-one (**5**).* To a solution of **4** (2.48 g, 12.9 mmol, 1 eq.) in THF (60 mL) was added NaH (60Wt% in mineral oil, 1.22 g, 30.5 mmol, 2.4 eq.) in THF (5 mL). The mixture was cooled to 0 °C and MeI (2.4 mL, 5.4 g, 38 mmol, 2.9 eq.) was added dropwise followed by stirring for 1.5 hours at 23 °C. Afterwards the reaction mixture was poured in water (60 mL) and was made slightly acidic by addition of aqueous of HCl (2 M). The product was extracted with Et_2O/THF (1:1, 120 mL) and then with Et_2O (3 x 30 mL). The combined organic layer were washed with sat. $NaHCO_3$, dried with Na_2SO_4 and evaporated *in vacuo* to give a crude orange/brown powder. The crude product was purified by column chromatography from Heptane/AcOEt (9:1 v/v) to yield the slightly yellow crystals **5** (2.42 g, 85%). **^{1}H NMR** (400 MHz, $CDCl_3$) δ 6.98 (d, *J* = 8.7 Hz, 1H, 6-C*H*), 6.73 (d, *J* = 8.8 Hz, 1H, 7-C*H*), 3.89 (s, 3H, 11-CH_3), 3.83 (s, 3H, 10-CH_3), 2.85 (s, 2H, 3-CH_2), 1.20 (s, 6H, 12-CH_3) ppm. **^{13}C NMR** (101 MHz, $CDCl_3$) δ 209.3 (1-*C*), 152.1 (8-*C*), 150.4 (5-*C*), 142.8 (4-*C*), 124.5 (9-*C*), 116.6 (6-*C*), 109.5 (7-*C*), 56.0 (11-*C*), 55.8 (10-*C*), 45.5 (2-*C*), 39.2 (3-*C*), 25.5 (12-*C*) ppm. ***R*f** 0.38 (AcOEt/Hept, 2:3 v/v). **m.p.** 71 °C. **IR (FTIR, solid)** 3285, 3076, 2929, 2871, 2840, 1699, 1639, 1596, 1535, 1494, 1458, 1263, 1161, 1098, 1066, 996, 919, 811, 717, 673, 571 cm^{-1}. **HR-MS (GC-EI-MS)** [$C_{13}H_{16}O_3^+$] calc. 220.10994, found 220.10939.

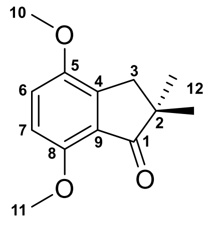

*(E)/(Z)-4,6-dichloro-2-(4,7-dimethoxy-2,2-dimethyl-2,3-dihydro-1H-inden-lidene)benzo[b]thiophen-3(2H)-one (**6**)*. **3** (0.822 g, 3.75 mmol, 1.4 eq.) was dissolved in dry CH_2Cl_2 (30 mL) under Ar atmosphere and cooled to -78 °C before BCl_3 (0.822 g, 3.75 mmol, 1.4 eq.) was added. This mixture was then added to a solution of **5** (0.592 g, 2.69 mmol, 1 eq.) in dry CH_2Cl_2 (6 mL) at 0 °C and under Ar atmosphere. After the mixture was stirred for 50 min at 0 °C, a saturated solution of $NaHCO_3$ (250 mL) was added and extracted with CH_2Cl_2 (3 x 200 mL). The combined organic layers were dried with Na_2SO_4 and evaporated *in vacuo*. The crude product **6** was obtained as a yellow solid (1.43 g, 91%). The crude product was used in the subsequent step. **E-isomer: ^{1}H NMR** (400 MHz, CD_2Cl_2) δ 7.39 (d, *J* = 1.7 Hz, 1H, 4-C*H*/6-C*H*), 7.23 (d, *J* = 1.7 Hz, 1H, 4-C*H*/6-C*H*), 6.91 (d, *J* = 8.8 Hz, 1H, 15-C*H*), 6.74 (d, *J* = 8.9 Hz, 1H, 14-C*H*), 3.82 (s, 3H, 19-CH_3), 3.80 (s, 3H, 18-CH_3), 3.00 (d, *J* = 15.8 Hz, 1H, 11-C*H*H), 2.86 (d, *J* = 15.8 Hz, 1H, 11-CH*H*), 1.66 (s, 3H, 20-CH_3), 1.19 (s, 3H, 21-CH_3) ppm. **^{13}C NMR** (101 MHz, CD_2Cl_2) δ 184.3 (1-*C*), 157.6 (9-*C*), 152.9 (16-*C*), 150.2 (13-*C*), 147.9 (8-*C*), 139.7 (2-*C*/5-*C*), 136.8 (17-*C*), 134.7 (3-*C*/7-*C*), 129.4 (12-*C*), 127.2 (4-*C*/6-*C*), 126.7 (2-*C*/5-*C*), 125.3 (3-*C*/7-*C*), 122.4 (4-*C*/6-*C*), 114.5 (15-*C*), 110.0 (14-*C*), 56.5 (19-*C*), 55.6 (18-*C*), 50.8 (10-*C*), 46.2 (11-*C*), 26.8 (20-*C*), 26.2 (21-*C*) ppm. **Z-isomer: ^{1}H NMR** (400 MHz, CD_2Cl_2) δ 7.29 (d, *J* = 1.7 Hz, 1H, 4-C*H*/6-C*H*), 7.23 7.16 (d, *J* = 1.7 Hz, 1H, 4-C*H*/6-C*H*), 6.96 (d, *J* = 8.9 Hz, 1H, 14-C*H*), 6.79 (d, *J* = 8.9 Hz, 1H, 15-C*H*), 3.90 (s, 3H, 18-CH_3), 3.81 (s, 3H, 19-CH_3), 2.90 (s, 2H, 11-CH_2), 1.54 (s, 3H, 20-CH_3/21-CH_3), 1.52 (s, 3H, 20-CH_3/21-CH_3) ppm. **^{13}C NMR** (101 MHz, CD_2Cl_2) δ 186.2 (1-*C*), 163.6 (9-*C*), 150.8 (16-*C*), 150.6 (8-*C*), 150.5 (13-*C*), 140.0 (2-*C*/5-*C*), 138.1 (12-*C*), 134.6 (3-*C*/7-*C*), 129.1 (17-*C*), 128.4 (3-*C*/7-*C*), 126.8 (4-*C*/6-*C*), 126.5 (2-*C*/5-*C*), 121.8 (4-*C*/6-*C*), 115.6 (14-*C*), 110.5 (15-*C*), 56.4

(19-*C*), 55.4 (18-*C*), 51.9 (10-*C*), 48.1 (11-*C*), 27.0 (20-*C* + 21-*C*) ppm. ***R*f** 0.26 (AcOEt/Hept, 1:9 v/v). **IR (FTIR, solid)** 3070, 2956, 2924, 2853, 1719, 1672, 1572, 1535, 1490, 1461, 1436, 1369, 1292, 1261, 1237, 1217, 1190, 1172, 1117, 1080, 962, 819, 796, 718, 683, 659, 640, 608, 523 cm^{-1}. **HR-MS (ESI-MS)** [$C_{21}H_{19}Cl_2O_3S^+$] calc. 421.04319, found 421.04445.

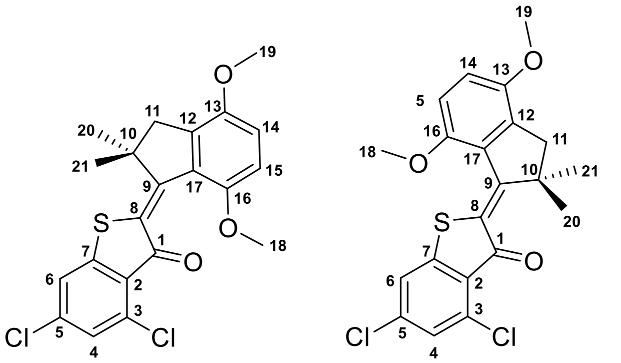

(E)/(Z)-4,6-dichloro-2-(4,7-dimethoxy-2,2-dimethyl-2,3-dihydro-1H-inden-1-ylidene)benzo[b]thiophen-3(2H)-one 1-oxide (7). The crude mixture of **6** (0.982 g, 2.33 mmol, 1 eq.) was dissolved in a mixture of 5% AcOEt in concentrated acetic acid (50 mL), after which sodium perborate tetrahydrate (1.43 g, 9.32 mmol, 4.2 eq.) was added. The mixture was stirred for 5 h at 23 °C. Then a saturated solution of $NaHCO_3$ (550 mL) was added and was extracted with AcOEt (3 x 300 mL). The combined organic layers were dried with Na_2SO_4 and evaporated in *vacuo*. The crude product was purified by column chromatography from Heptane/AcOEt (9:1 → 100% AcOEt, v/v) to yield **7** (717 mg, 70%) as the E-isomer (395 mg, 38.6%) and Z-isomer (322 mg, 31.4%). **E-isomer:** ^{1}**H NMR** (400 MHz, CD_2Cl_2) δ 7.96 (d, *J* = 1.7 Hz, 1H, 6-C*H*), 7.67 (d, *J* = 1.7 Hz, 1H, 4-C*H*), 7.00 (d, *J* = 8.9 Hz, 1H, 14-C*H*), 6.76 (d, *J* = 8.9 Hz, 1H, 15-C*H*), 3.83 (s, 3H, 19-C*H3*), 3.79 (s, 3H, 18-C*H3*), 3.08 (d, *J* = 15.7 Hz, 1H, 11-C*H*H), 2.92 (d, *J* = 15.7 Hz, 1H, 11-CH*H*), 1.91 (s, 3H, 21-C*H3*), 1.37 (s, 3H, 20-C*H3*) ppm. ^{13}**C NMR** (101 MHz, CD_2Cl_2) δ 179.8 (1-*C*), 170.4 (9-*C*), 153.5 (16-*C*), 153.3 (8-*C*), 150.4 (13-*C*), 141.1 (5-*C*), 140.2 (7-*C*), 138.1 (12-*C*), 134.4 (4-*C*), 133.8 (3-*C*), 130.7 (2-*C*), 128.8 (17-*C*), 126.2 (6-*C*), 116.5 (14-*C*), 110.1 (15-*C*), 56.6 (19-*C*), 55.7 (18-*C*), 52.7 (10-*C*), 46.8 (11-*C*), 28.9 (21-

C), 26.0 (20-*C*) ppm. ***R*f** 0.34 (AcOEt/Hept, 3:7 v/v). **IR (FTIR, solid)** 3078, 3005, 2963, 2928, 2840, 1699, 1580, 1540, 1495, 1440, 1422, 1381, 1300, 1263, 1192, 1157, 1157, 1133, 1116, 1082, 1065, 1036, 1007, 952, 885, 864, 830, 806, 757, 718, 691, 664, 646, 581, 567, 547, 532 cm^{-1}. **HR-MS (ESI-MS)** [$C_{21}H_{18}Cl_2NaO_4S^+$] calc. 459.02005, found 459.01948. **Z-isomer: ^{1}H NMR** (400 MHz, CD_2Cl_2) δ 7.89 (d, *J* = 1.8 Hz, 1H, 6-C*H*), 7.61 (d, *J* = 1.8 Hz, 1H, 4-C*H*), 7.06 (dd, *J* = 8.9, 1.0 Hz, 1H, 14-C*H*), 6.85 (d, *J* = 8.9 Hz, 1H, 15-C*H*), 4.03 (s, 3H, 18-CH_3), 3.83 (s, 3H, 19-CH_3), 3.00 (d, *J* = 16.5 Hz, 1H, 11-C*H*H), 2.91 (d, *J* = 16.5 Hz, 1H, 11-CH*H*), 1.52 (s, 3H, 20-CH_3), 1.51 (s, 3H, 21-CH_3) ppm. **^{13}C NMR** (101 MHz, CD_2Cl_2) δ 181.5 (1-*C*), 170.6 (9-*C*), 154.5 (8-*C*), 151.3 (16-*C*), 150.2 (13-*C*), 142.8 (7-*C*), 140.9 (5-*C*), 138.7 (12-*C*), 134.7 (4-*C*), 133.4 (3-*C*), 130.1 (2-*C*), 126.0 (17-*C*), 125.1 (6-*C*), 116.6 (14-*C*), 109.4 (15-*C*), 56.0 (18-*C*), 55.9 (19-*C*), 51.9 (10-*C*), 47.0 (11-*C*), 28.2 (20-*C*), 25.1 (21-*C*) ppm. ***R*f** 0.14 (AcOEt/Hept, 3:7 v/v). **IR (FTIR, solid)** 3071, 3049, 2940, 2921, 2837, 1719, 1673, 1573, 1538, 1492, 1293, 1261, 1181, 1085, 1066, 1046, 1030, 992, 860, 826, 803, 717, 691, 659, 614, 571, 503 cm^{-1}. **HR-MS (ESI-MS)** [$C_{21}H_{19}Cl_2O_4S^+$] calc. 437.03811, found 437.03907; [$C_{21}H_{18}Cl_2NaO_4S^+$] calc. 459.02005, found 459.02020.

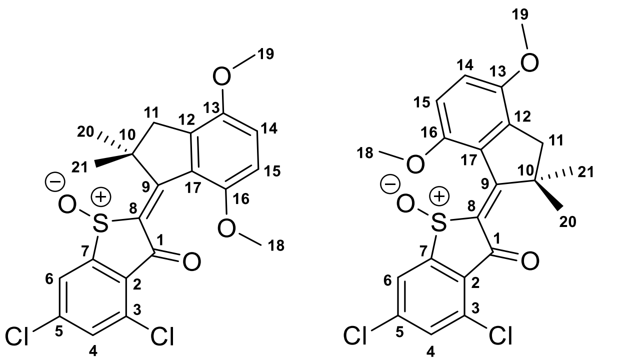

*2-(4-(azidomethyl)phenyl)-4,4,5,5-tetramethyl-1,3,2-Dioxaborolane (**9**)*. **8** (2.00 g, 6.73 mmol, 1 eq.) was dissolved in EtOH (80 mL) and sodium azide (619 mg, 9.52 mmol, 1.4 eq.) was added. The mixture was stirred for 24 h at 23 °C. The mixture was evaporated and co-evaporated with Petroleum ether (80-100). Then $MgSO_4$ and Petroleum ether (80-100) were

added and the suspension was plugged through a pad of silica on celite, and washed with Petroleum ether (80-100) and pentane. The organic layers was evaporated *in vacuo*. Recrystallization of the crude product from Petroleum ether (80-100) yielded **9** as colourless needles (1.29 g, 74%). **^{1}H NMR** (400 MHz, $CDCl_3$) δ 7.86 – 7.77 (m, 2H, 2-C*H*), 7.32 (m, 2H, 3-C*H*), 4.35 (s, 2H, 5-CH_2), 1.35 (s, 12H, 7-CH_3) ppm. **^{13}C NMR** (101 MHz, $CDCl_3$) δ 138.3 (4-*C*), 135.3 (2-*C* + 3-*C*), 127.4 (1-*C*), 83.9 (6-*C*), 54.8 (5-*C*), 24.8 (7-*C*) ppm. ***R*f** 0.39 (AcOEt/Hept, 1:9 v/v). **IR (FTIR, solid)** 2979, 2931, 2096, 1614, 1517, 1399, 1359, 1323, 1274, 1143, 1089, 1022, 962, 859, 821, 788, 730, 656 cm^{-1}. **HR-MS (ESI-MS)** [$C_{13}H_{18}BN_3O_2^{+}$] calc. 259.14921, found 259.14928.

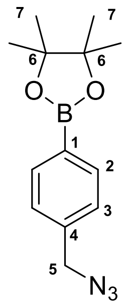

*(Z)-4-(4-(azidomethyl)phenyl)-6-chloro-2-(4,7-dimethoxy-2,2-dimethyl-2,3-dihydro-1H-inden-1-ylidene)benzo[b]thiophen-3(2H)-one 1-oxide **(10a)** and (Z)-6-(4-(azidomethyl)phenyl)-4-chloro-2-(4,7-dimethoxy-2,2-dimethyl-2,3-dihydro-1H-inden-1-ylidene)benzo[b]thiophen-3(2H)-one 1-oxide **(10b)**.* Z-**7** (198 mg, 0.453 mmol, 1 eq.) was dissolved in a water/THF mixture (1:5, 36 mL), Argon was bubbled through and an aqueous solution of 2 M K_2CO_3 (680 µL, 188 mg, 1.36 mmol, 3 eq.) was added. **9** (306 mg, 1.18 mmol, 2.6 eq.) and $Pd(PPh_3)_4$ (26 mg, 22 µmol, 0.05 eq.) were added and the mixture was stirred for 4 h at 60 °C. After cooling, a saturated solution of NH_4Cl (250 mL) was added and the reaction mixture was extracted with AcOEt (1 x 250mL, 2 x 200 mL). The combined organic layers were dried over $MgSO_4$ and evaporated *in vacuo*. The crude product was purified by column chromatography from Heptane/AcOEt (1:1 v/v) to obtain yellow/orange solids of **10** (159 mg,

66%) consisting of **10a** (35 mg, 14%) and **10b** (124 mg, 51%). **10a:** ^{1}**H NMR** (400 MHz, CD_2Cl_2) δ 8.13 (s, 1H, 6-C*H*), 7.85 (s, 1H, 4-C*H*), 7.74 (d, *J* = 7.9, 2H, 23-C*H*); 7.49 (d, *J* = 8.0, 2H, 24-C*H*); 7.06 (d, *J* = 8.8, 1H, 14-C*H*), 6.86 (d, *J* = 8.9 Hz, 1H, 15-C*H*), 4.45 (s, 2H, 26-C*H₂*), 4.05 (s, 3H, 18-CH_3), 3.83 (s, 3H, 19-CH_3), 3.00 (d, *J* = 16.4 Hz, 1H, 11-C*H*H), 2.92 (d, *J* = 16.5 Hz, 1H, 11-CH*H*), 1.56 (s, 3H, 20-CH_3/21-CH_3), 1.55 (s, 3H, 20-CH_3/21-CH_3) ppm. ^{13}**C NMR** (101 MHz, CD_2Cl_2) δ 182.1 (1-*C*), 169.8 (9-*C*), 154.1 (8-*C*), 151.3 (16-*C*), 150.1 (13-*C*), 147.7 (5-*C*), 143.4 (7-*C*), 138.6 (12-*C*), 137.6 (22-*C*), 137.1 (25-*C*), 131.9 (4-*C*), 132.6 (2-*C*), 130.1 (3-*C*), 129.0 (24-*C*), 127.8 (23-*C*), 123.0 (6-*C*), 126.2 (17-*C*), 116.4 (14-*C*), 109.4 (15-*C*), 55.9 (19-*C*), 55.0 (18-*C*), 54.3 (26-*C*), 51.8 (10-*C*), 47.1 (11-*C*), 28.2 (20-*C*/21-*C*), 25.2 (20-*C*/21-*C*) ppm. $\boldsymbol{R}_f$ 0.18 (AcOEt/Hept, 1:1 v/v). **IR (FTIR, solid)** 2988, 2962, 2923, 2902, 2839, 2087, 1681, 1589, 1541, 1494, 1446, 143, 1380, 125, 1222, 1188, 1098, 1044, 1064, 958, 909, 880, 825, 803, 718, 697, 616, 560, 523 cm^{-1}. **HR-MS (GC-EI-MS)** [$C_{28}H_{25}ClN_3O_4S^+$] calc. 534.12543, found 534.12461; [$C_{28}H_{24}ClN_3NaO_4S^+$] calc. 556.10737, found 556.10673. **10b:** ^{1}**H NMR** (400 MHz, CD_2Cl_2) δ 7.98 (d, *J* = 1.9 Hz, 1H, 6-C*H*), 7.51 (d, *J* = 1.8 Hz, 1H, 4-C*H*), 7.47 – 7.41 (m, 4H, 23-C*H* + 24-C*H*), 7.05 (d, *J* = 8.9 Hz, 1H, 14-C*H*), 6.85 (d, *J* = 8.8 Hz, 1H, 15-C*H*), 4.49 (s, 2H, 26-CH_2), 4.04 (s, 3H, 17-CH_3), 3.83 (s, 3H, 19-CH_3), 2.99 (d, *J* = 16.3 Hz, 1H, 11-C*H*H), 2.88 (d, *J* = 16.3 Hz, 1H, 11-CH*H*), 1.49 (s, 3H, 20-CH_3/21-CH_3), 1.42 (s, 3H, 20-CH_3/21-CH_3) ppm. ^{13}**C NMR** (101 MHz, CD_2Cl_2) δ 183.4 (1-*C*), 169.6 (9-*C*), 153.7 (8-*C*), 151.2 (16-*C*), 150.1 (13-*C*), 143.0 (7-*C*), 142.6 (3-*C*), 140.3 (5-*C*), 138.4 (12-*C*), 136.8 (22-*C*), 136.1 (25-*C*), 134.0 (4-*C*), 130.8 (2-*C*), 129.6 (24-*C*), 127.7 (23-*C*), 125.5 (6-*C*), 126.5 (17-*C*), 116.3 (14-*C*), 109.4 (15-*C*), 55.9 (19-*C*), 55.0 (18-*C*), 54.5 (26-*C*), 51.9 (10-*C*), 46.9 (11-*C*), 28.3 (20-*C*/21-*C*), 25.3 (20-*C*/21-*C*) ppm. $\boldsymbol{R}_f$ 0.25 (AcOEt/Hept, 1:1 v/v). **IR (FTIR, solid)** 2962, 2925, 2854, 2095, 1734 1674, 1543, 1492, 1436, 1360, 1261, 1217, 1093, 1057, 1042, 1019, 955, 880, 795, 719, 701, 672, 639, 604, 544, 517 cm^{-1}. **HR-MS (ESI-MS)** [$C_{28}H_{25}ClN_3O_4S^+$] calc. 534.12543, found 534.12548.

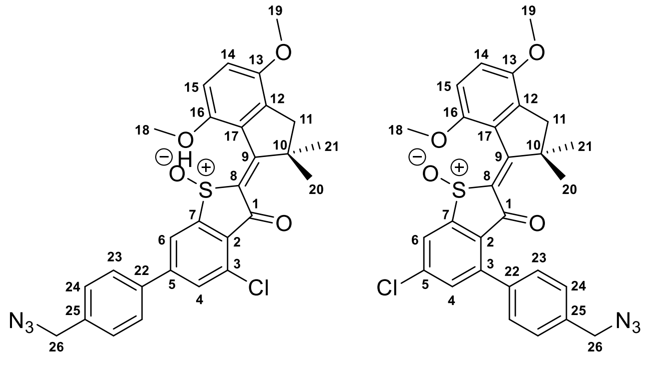

General preparation of polymersomes with DBCO handles. Modified from a previous report[49], a general procedure is described: MeO-PEG-*b*-PS (8 mg) and DBCO-PEG-*b*-PS (2 mg) were dissolved in a mixture of THF and 1,4-dioxane (1 mL, 4:1 v/v) in a 15 mL capped vial with a magnetic stir bar. After dissolving the solution for 0.5 h at 21 °C, a syringe pump equipped with a syringe and a needle was used to deliver ultrapure water with a rate of 1 mL/h for 0.5 h via a rubber septum, while vigorously stirring the mixture (900 rpm). Appearance of a cloudy suspension indicated formation of the polymersomes. Upon finishing the water addition, 8 mL of ultrapure water was added to the suspension, which ensured a rapid quenching of the PS domain within the bilayer of the polymersomes. The polymersomes were spun down via centrifuge (10 min, 10.000 rpm) and washed with ultrapure water a total of three times

Click Reaction on DBCO-PEG-*b*-PS Polymersomes. DBCO-PEG-b-PS polymersomes (10 mg, 20% functionalized) were diluted in MeOH (2 mL). An excess of **10b** in MeOH (100 µL,1 mg/mL) was added to the solution and the mixture was stirred for 16 h in the dark. The polymersomes were washed (3x) by centrifugation to remove the methanol and excess of **10b**, and were resuspended in ultrapure water.

Supplementary Tables and Figures

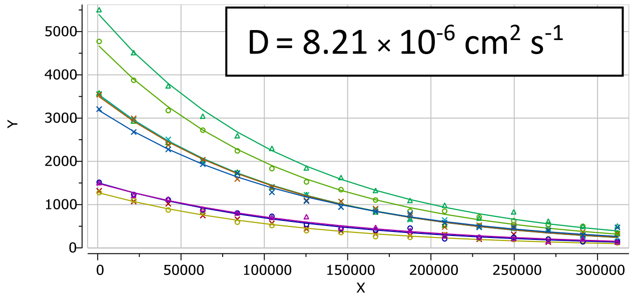

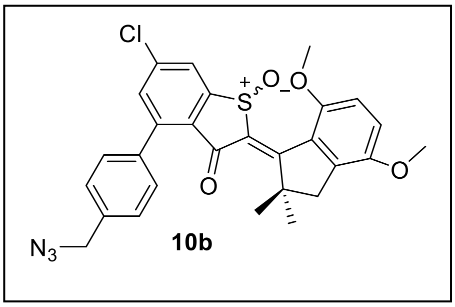

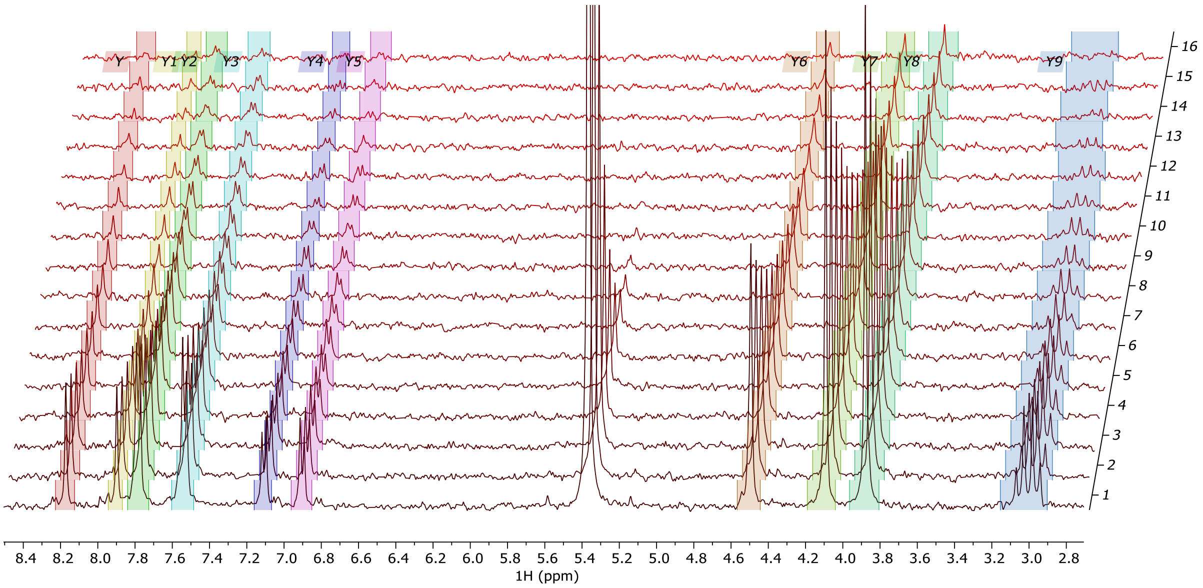

Figure S1 – ^{1}H NMR diffusion spectra (400 MHz, 298 K) of **10b** in CD_2Cl_2. Gradient strengths ranged from 5% to 95%. The absolute gradient strength was not calibrated prior to use as a relative comparison was all that was desired. D is the average diffusion coefficient of the molecule.

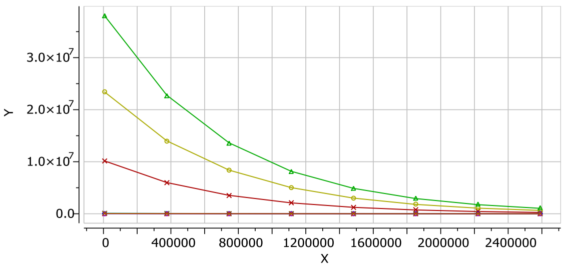

$D_{Y\text{-}Y2} = 1.40 \times 10^{-6}$ cm^2 s^{-1}

$D_{Y3\text{-}Y6} = 1.31 \times 10^{-6}$ cm^2 s^{-1}

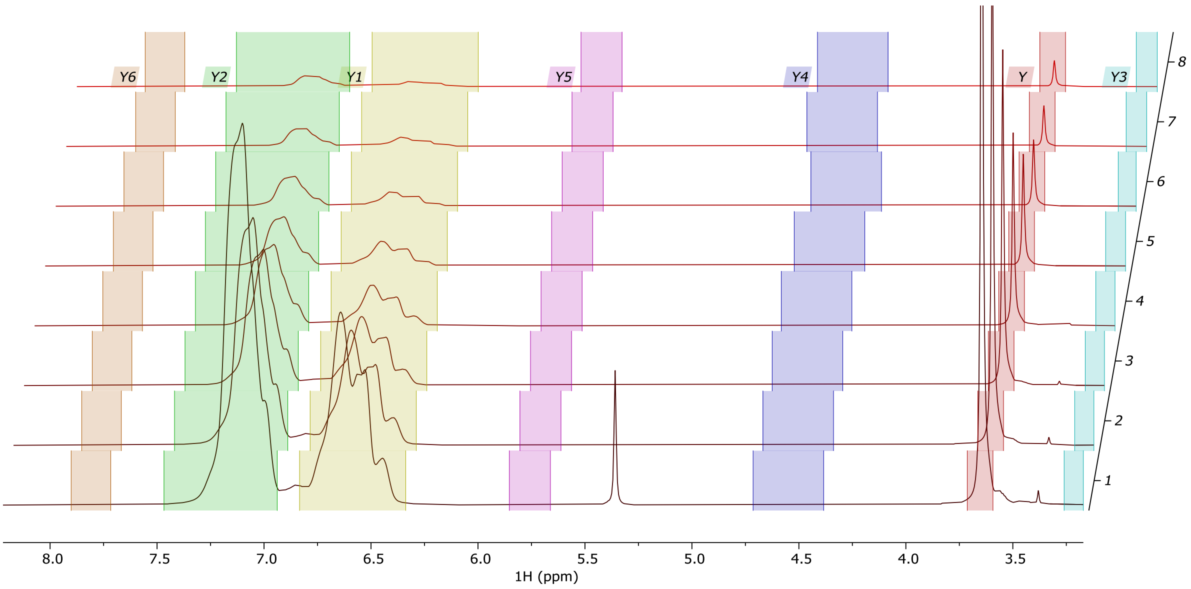

Figure S2 – ^{1}H NMR diffusion spectra (400 MHz, 298 K) of **10b**-poly(ethylene glycol)$_{44}$-*b*-polystyrene$_{176}$. Gradient strengths ranged from 5% to 95%. The absolute gradient strength was not calibrated prior to use as a relative comparison was all that was desired. $D_{Y\text{-}Y2}$ is the average diffusion coefficient of the polymer part of the molecule, while $D_{Y3\text{-}Y6}$ is the diffusion constant of the **10b** part. The diffusion coefficients are in the same order of magnitude and are ~10x lower than **10b** in solution, indicating successful binding.

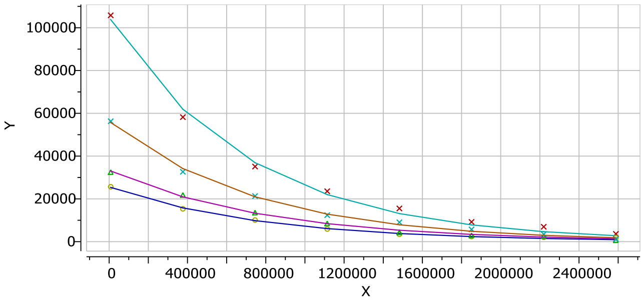

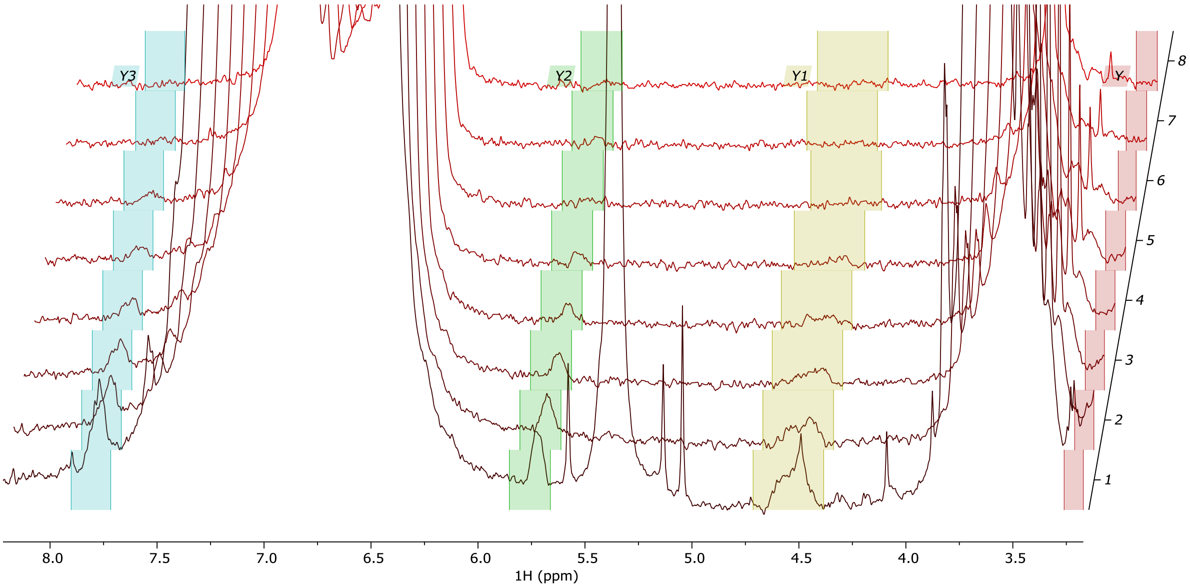

Figure S3 – ^{1}H NMR diffusion spectra (400 MHz, 298 K) of **10b**-poly(ethylene glycol)$_{44}$-*b*-polystyrene$_{176}$. Zoomed in on signals from **10b**. The absolute gradient strength was not calibrated prior to use as a relative comparison was all that was desired. Gradient strengths ranged from 5% to 95%.

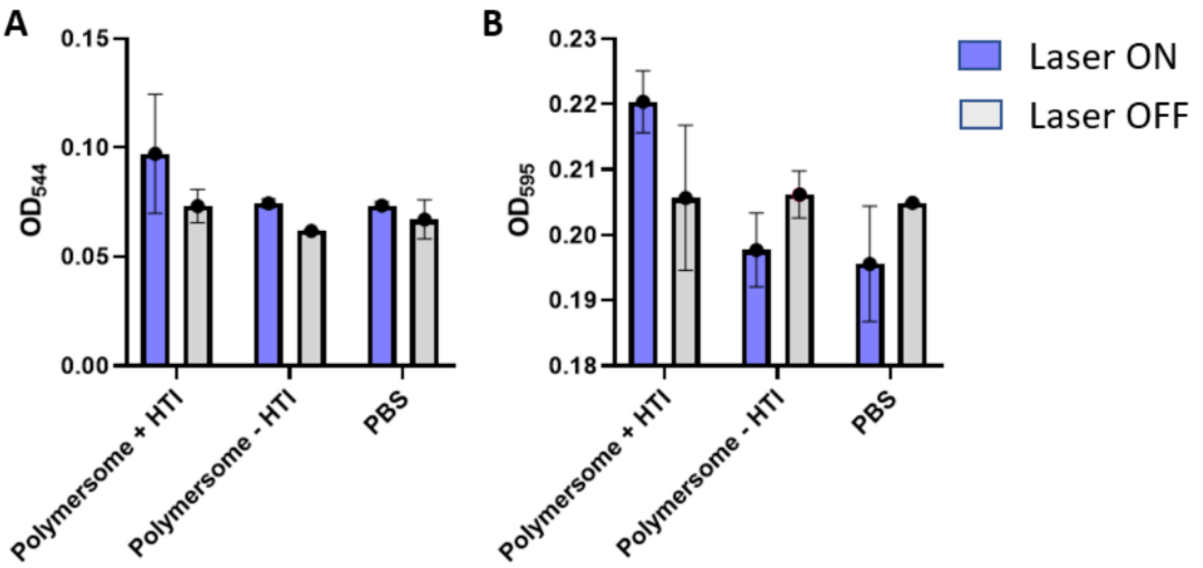

Figure S4. Polysaccharide (A) and Protein (B) content measured in supernatant after various treatments to determine release of ECM material. N = 6-8, no statistical significance could be detected which would indicate release of ECM material.

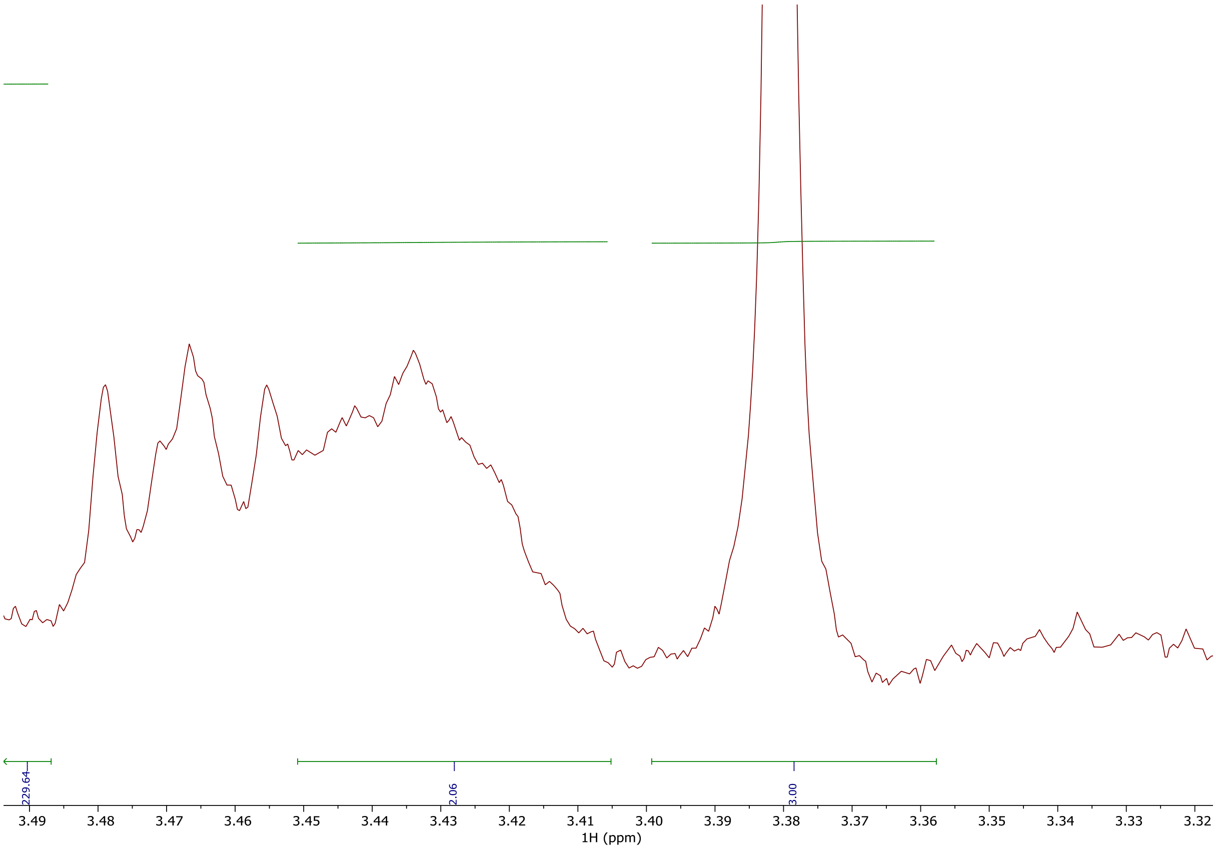

Figure S5 – ^{1}H qNMR spectrum of a 1/3rd 10b-PEG-*b*-PS polymer and 2/3rd MeO-PEG-*b*-PS polymer (400 MHz, NS = 128, CD_2Cl_2). In DCM, MeOH is at 3.42 ppm. There is a small peak, which was compared to the MeO-PEG-PS. Based on the 6.67 mg of MeO-PEG-PS measured, we calculated 3.03*10^-7 mol MeO-PEG-PS. This leads to 2.02*10^-7 mol MeOH, which is 6.27 ug of MeOH in the sample. This means that on our total sample of 3.33 mg 10b-PEG-PS, only 0.19% MeOH is present, which is near quantitative removal.

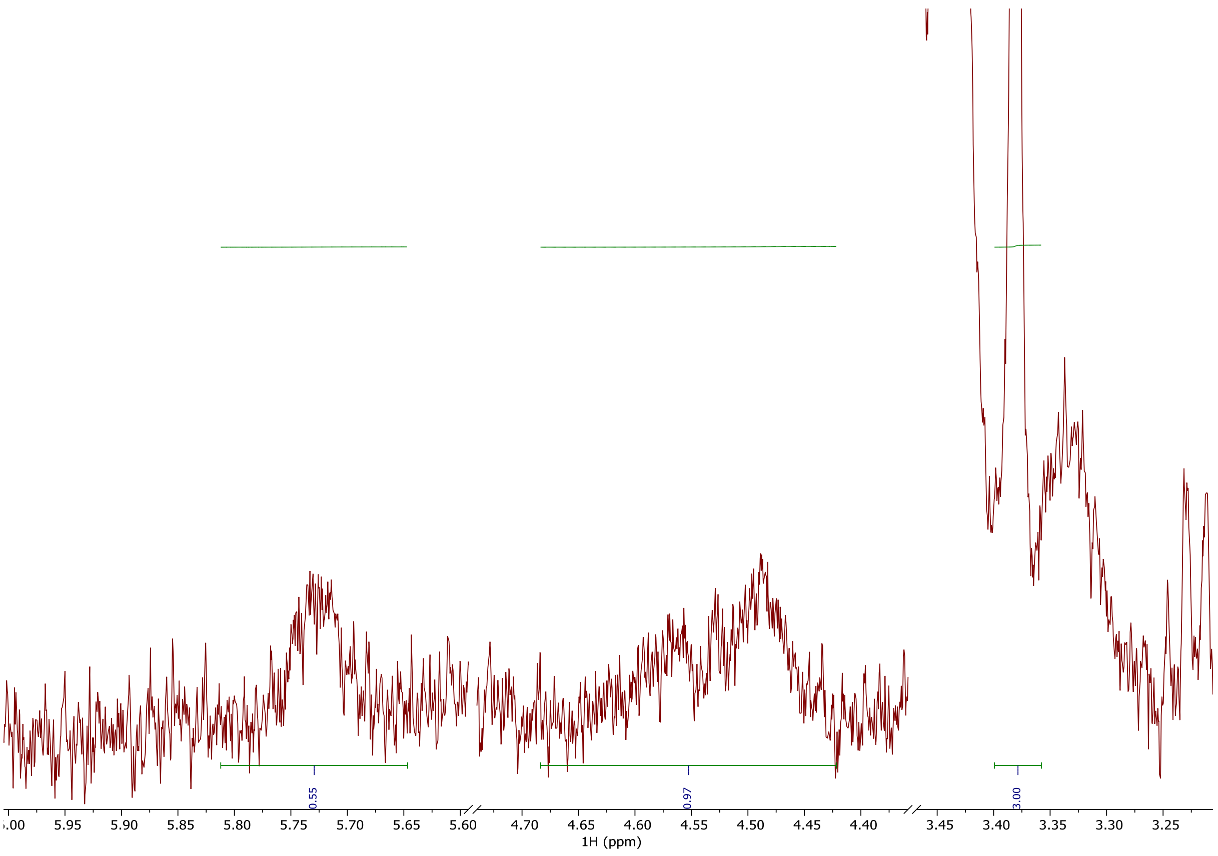

Figure S6 – ^{1}H qNMR spectrum of a 1/3rd 10b-PEG-*b*-PS polymer and 2/3rd MeO-PEG-*b*-PS polymer (400 MHz, NS = 128, CD_2Cl_2). By integrating the peak of the methoxy from the MeO-PEG-PS to 3, and then integrating 2 signals from the 10b which should be 1 and 2 protons respectively. However, since it's a 2/3 and 1/3 mix, the signals should be half, so 0.5 and 1. We observe integrals of 0.55 and 0.97, aligning with full conversion of the polymer and removal of excess 10b.

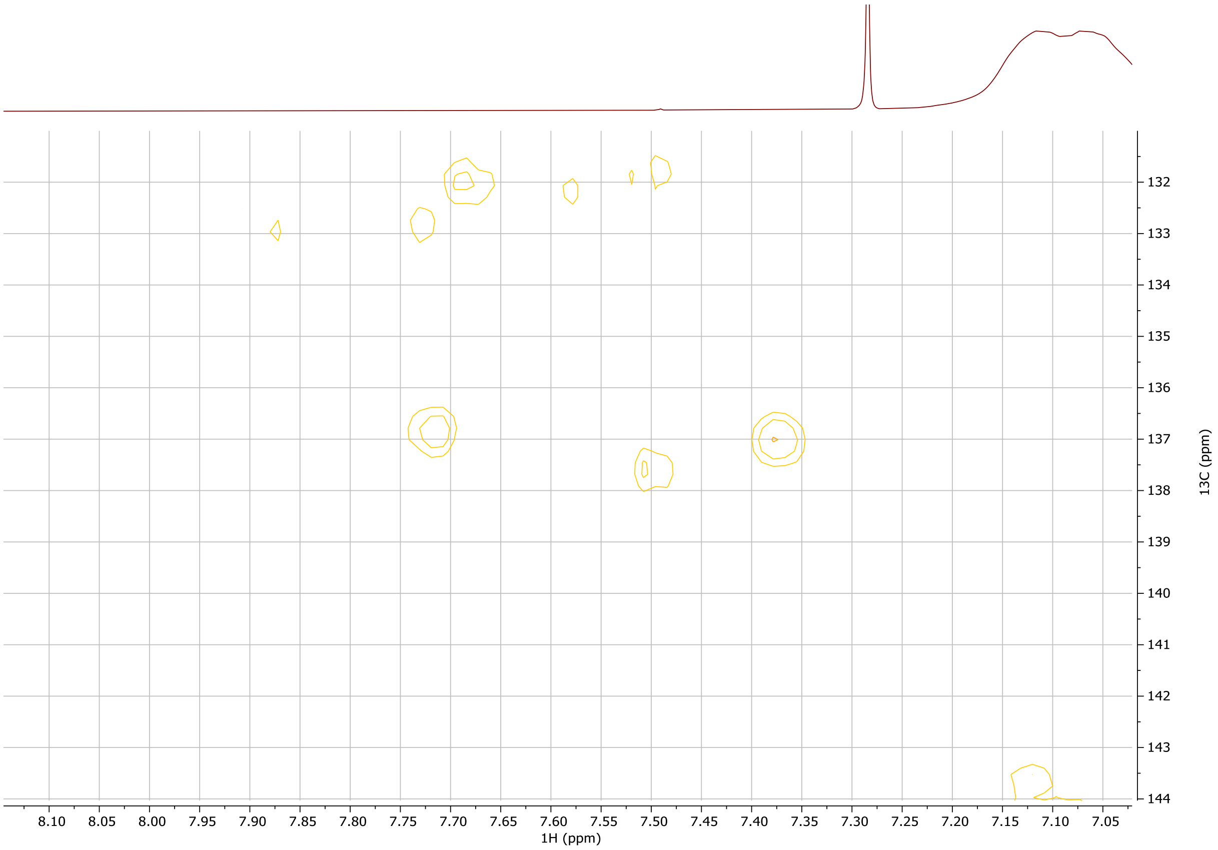
8.10
8.05
8.00
7.95
7.90
7.85
7.80
7.75
7.70
7.65
7.60
7.55
7.50
7.45
7.40
7.35
7.30
7.25
7.20
7.15
7.10
7.05
1H (ppm)
132
133
134
135
136
137
138
139
140
141
142
143
144
13C (ppm)

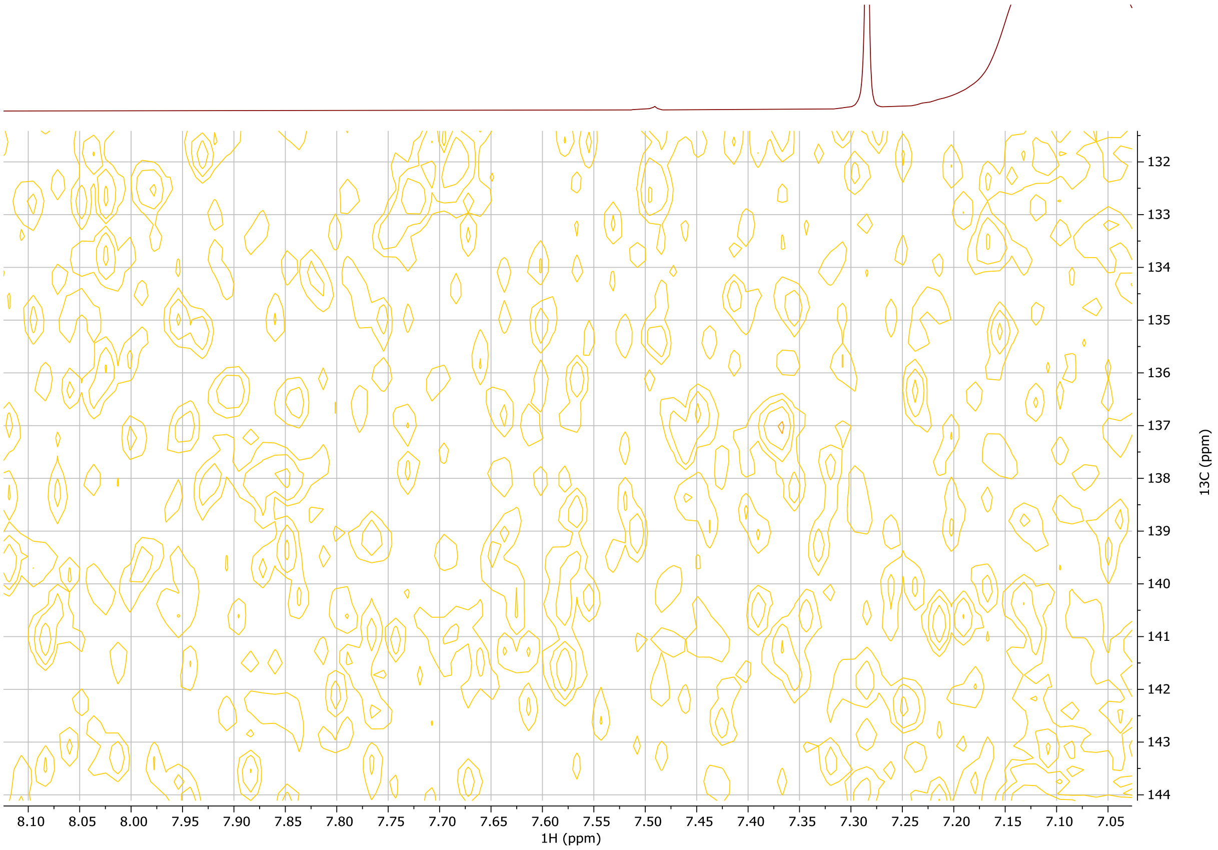
8.10
8.05
8.00
7.95
7.90
7.85
7.80
7.75
7.70
7.65
7.60
7.55
7.50
7.45
7.40
7.35
7.30
7.25
7.20
7.15
7.10
7.05
1H (ppm)
132
133
134
135
136
137
138
139
140
141
142
143
144
13C (ppm)

Figure S7 – ^{1}H-^{13}C HMBC spectrum of 10b-PEG-*b*-PS polymer (top) and DBCO-PEG-*b*-PS polymer (bottom). 500 MHz, NS = 192, $CDCl_3$, 128 increments. After performing the click reaction between DBCO of the polymer and N_3 from **10b**, the carbon peaks of the alkyne (85 ppm) shift to azide-bound alkene shifts. These are observed coupling to the aromatic signals of the DBCO at 137.0 and 137.5 ppm. Before the coupling, no signals are observed here above the noise

NMR, IR and MS data

2-((3,5-dichlorophenyl)thio)acetic acid) (**2**)

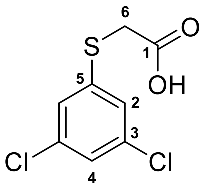

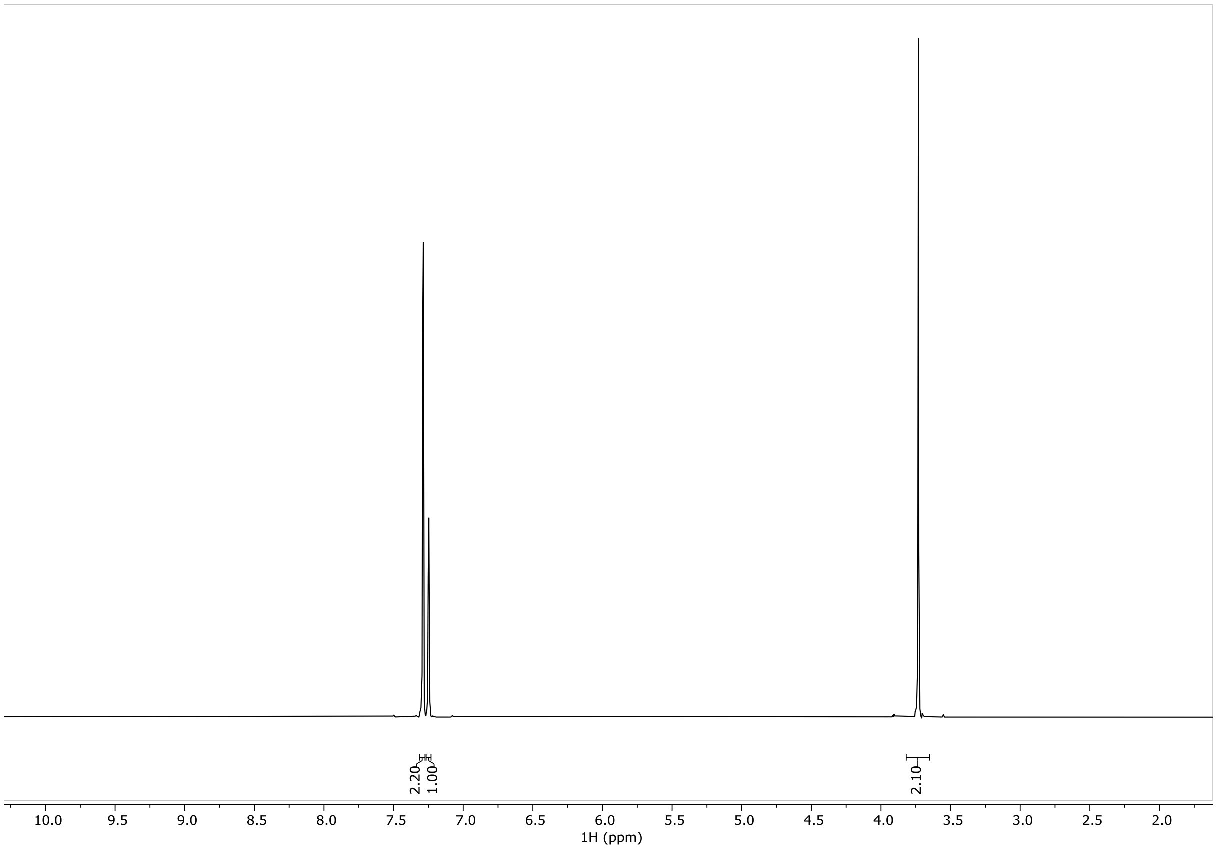
2.20
1.00
2.10
10.0 9.5 9.0 8.5 8.0 7.5 7.0 6.5 6.0 5.5 5.0 4.5 4.0 3.5 3.0 2.5 2.0
1H (ppm)

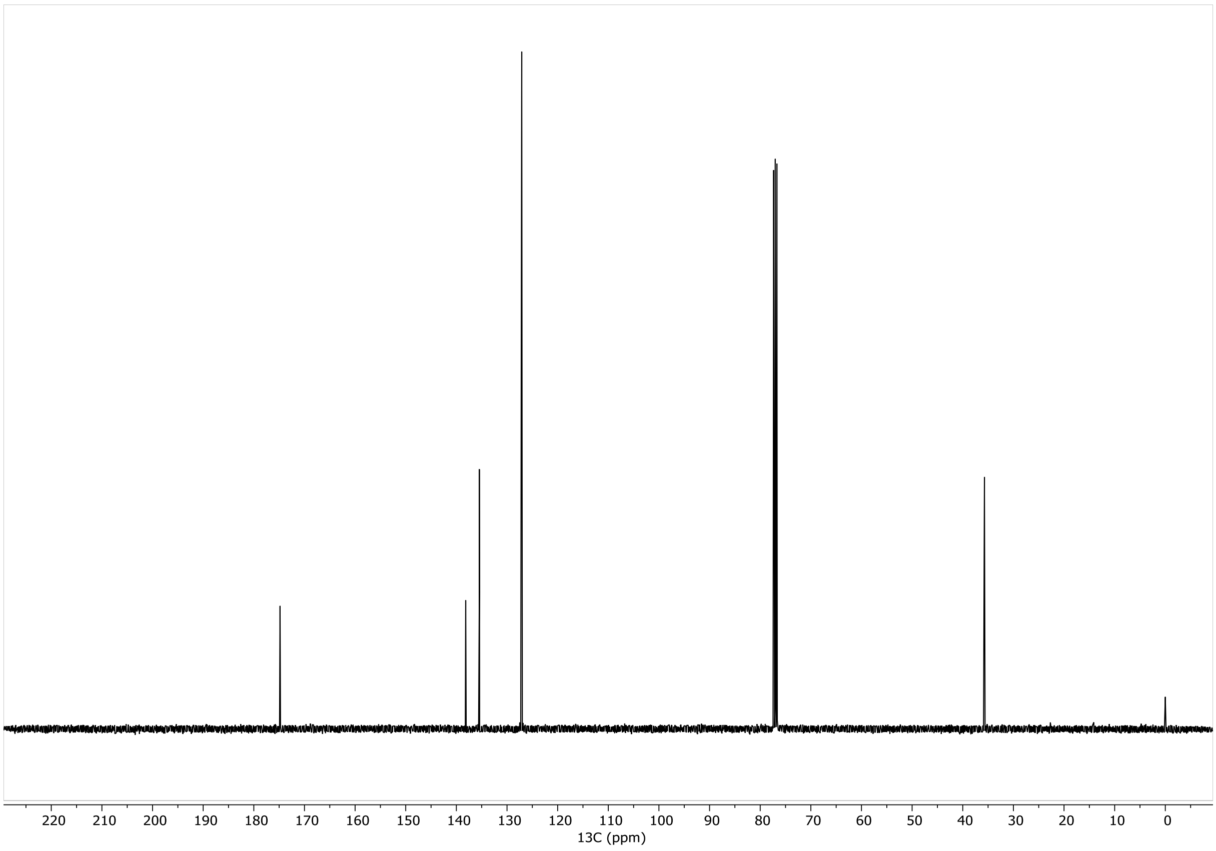
220 210 200 190 180 170 160 150 140 130 120 110 100 90 80 70 60 50 40 30 20 10 0
13C (ppm)

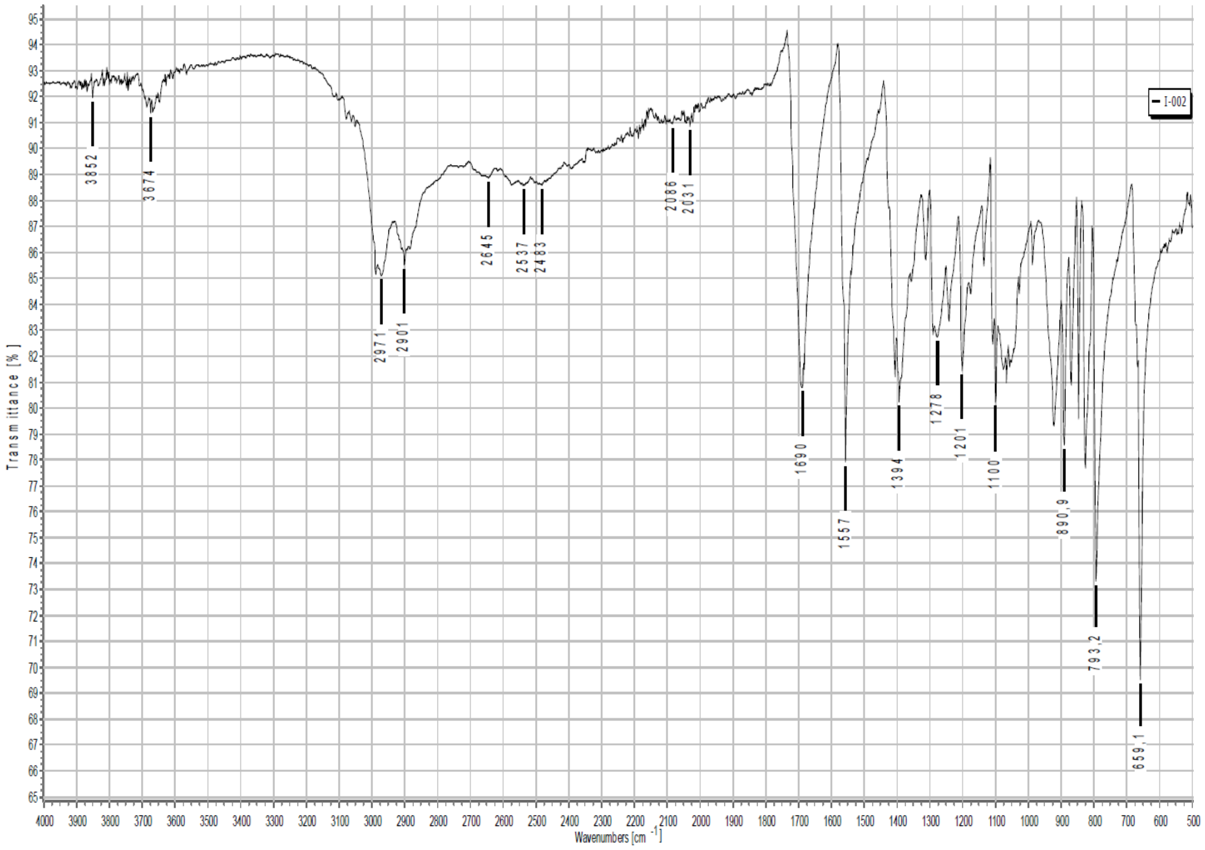

Transmittance [%]
Wavenumbers [cm -1]
I-002
3852
3674
2971
2901
2645
2537
2483
2086
2031
1690
1557
1394
1278
1201
1100
890,9
793,2
659,1

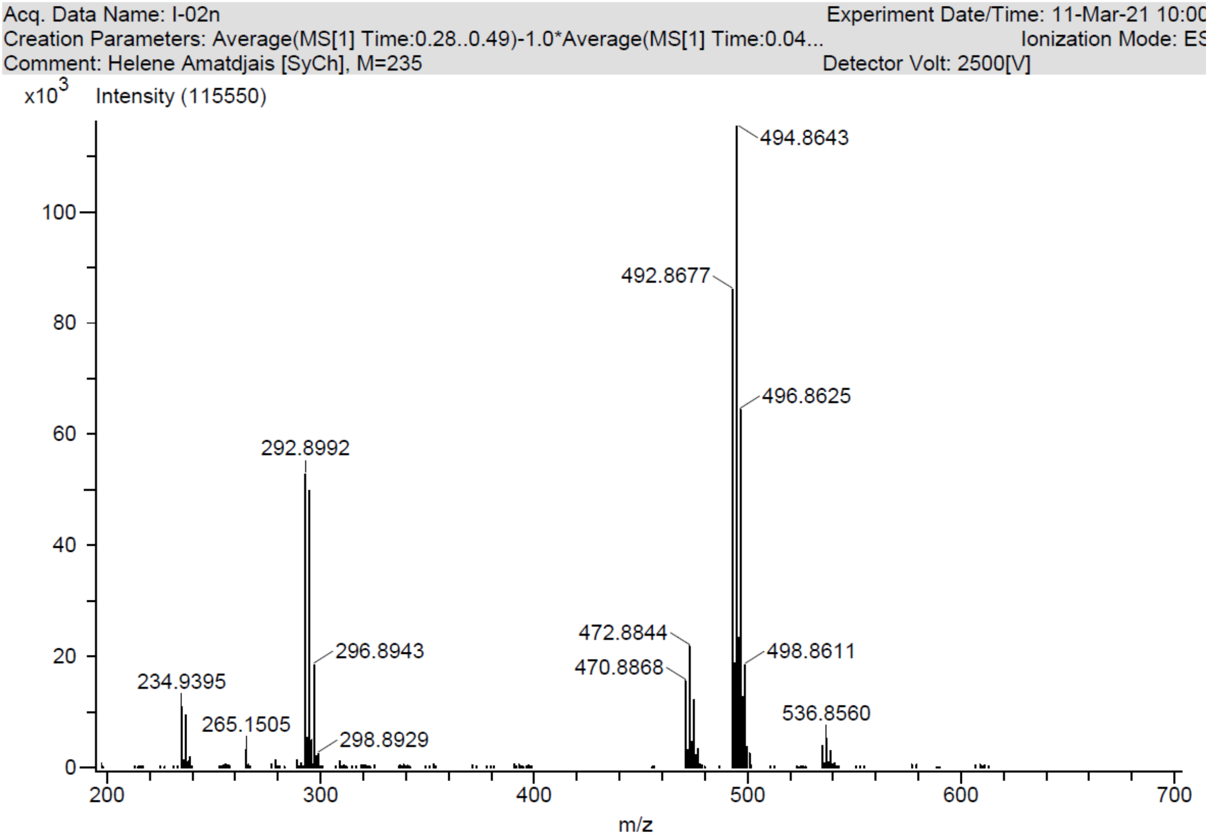

Acq. Data Name: I-02n
Experiment Date/Time: 11-Mar-21 10:00
Creation Parameters: Average(MS[1] Time:0.28..0.49)-1.0*Average(MS[1] Time:0.04...
Ionization Mode: ES
Comment: Helene Amatdjais [SyCh], M=235
Detector Volt: 2500[V]
x10^3 Intensity (115550)
494.8643
492.8677
496.8625
292.8992
472.8844
470.8868
498.8611
296.8943
234.9395
536.8560
265.1505
298.8929
m/z

4,6-dichlorobenzo[b]thiophen-3(2H)-one (**3**)

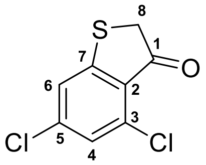

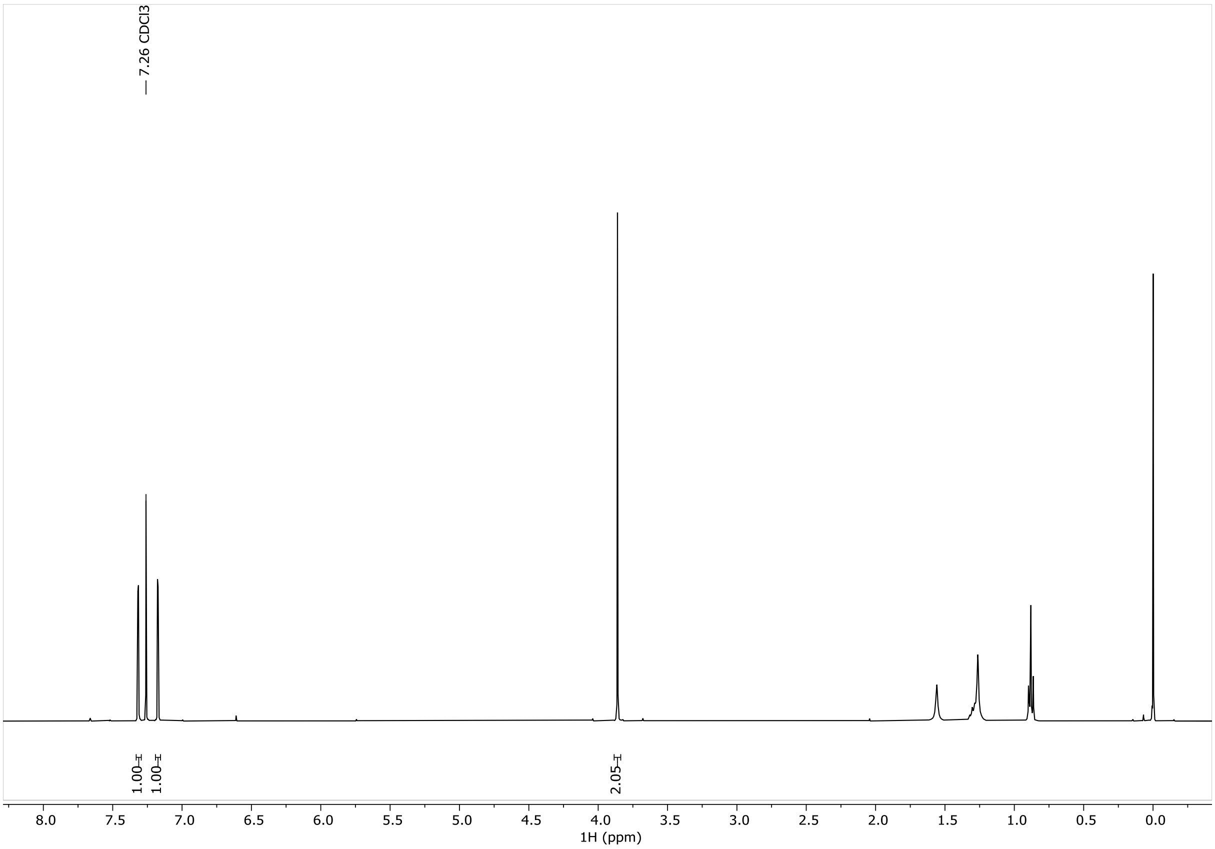

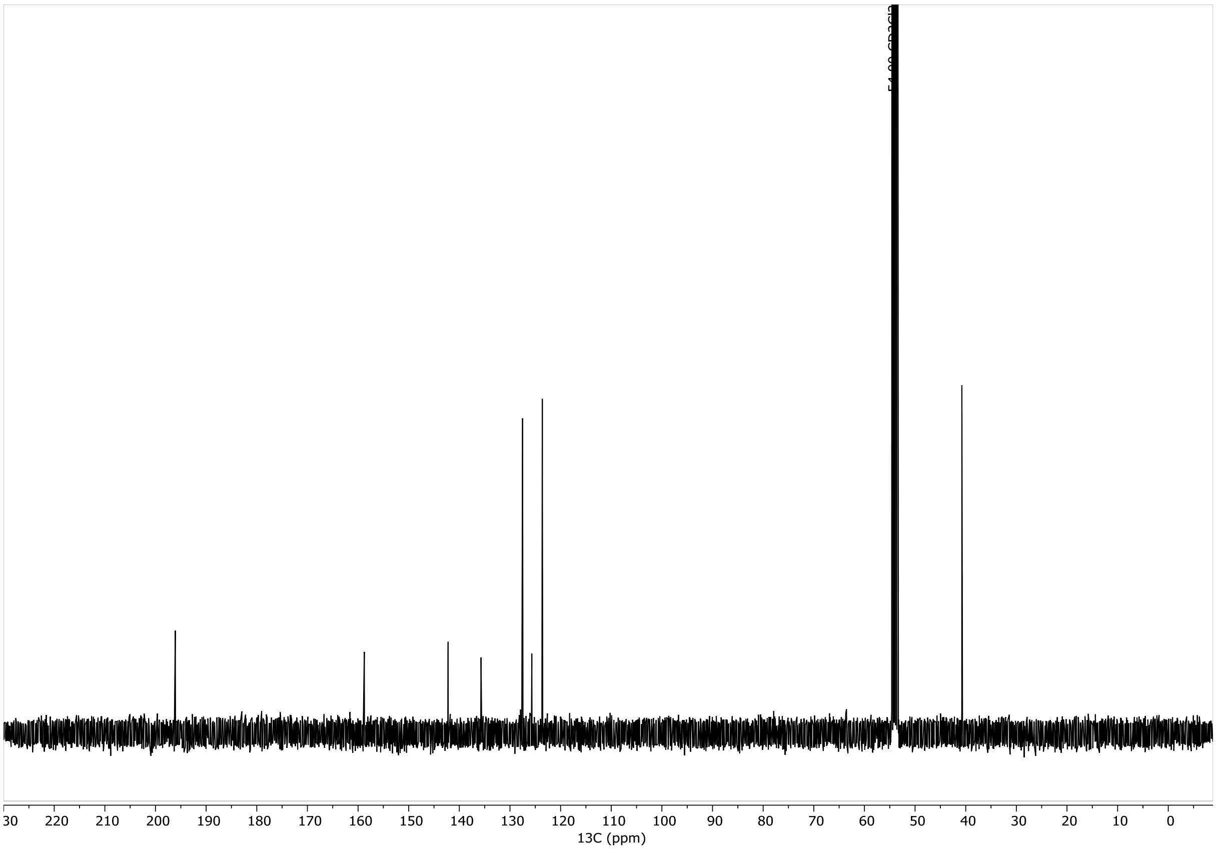
13C (ppm)

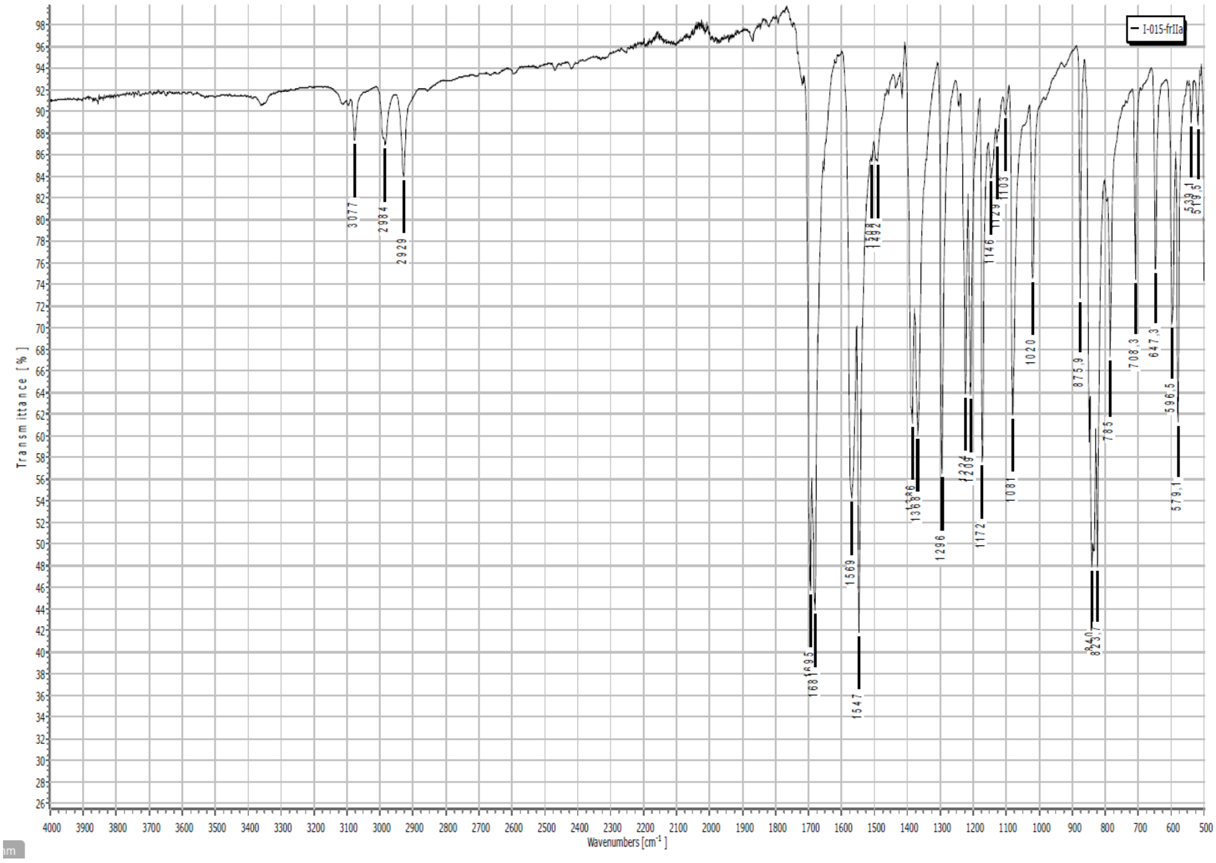
I-015-frIIa
Transmittance [%]
Wavenumbers [cm-1]
3077
2984
2929
1681
1695
1569
1547
1508
1492
1386
1368
1296
1224
1209
1172
1146
1129
1103
1081
1020
875.9
840
825
785
708.3
647.3
596.5
579.1

Acq. Data Name: HA_i-03b
Experiment Date/Time: 09-Apr-21 10:2...
Creation Parameters: Average(MS[1] Time:7.93..7.95)-1.0*Average(MS[1] Time:8...
Ionization Mode: EI+
Comment: Helene Amatdjais [DW], M=218

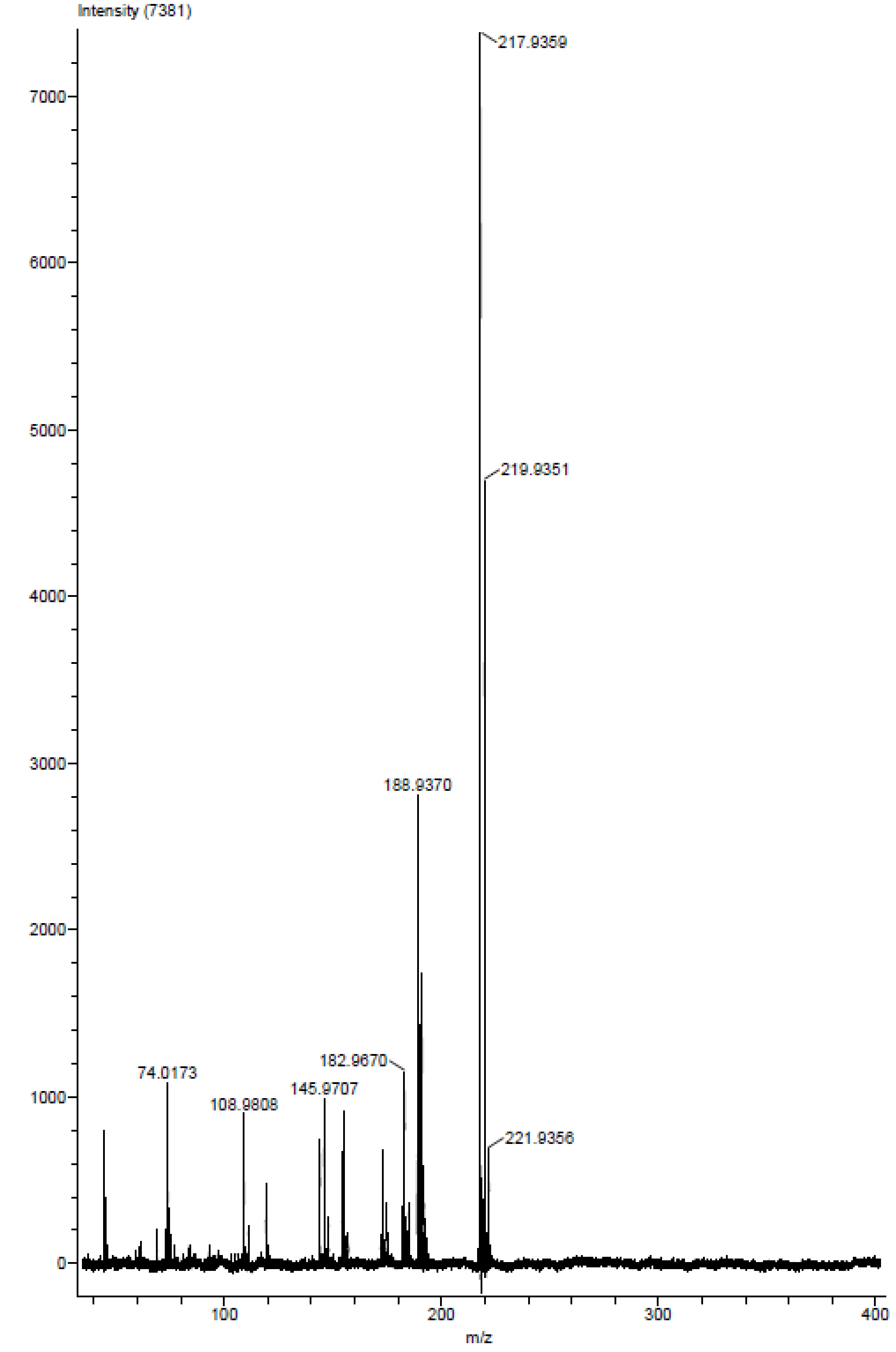

4,7-dimethoxy-2,2-dimethyl-2,3-dihydro-1H-inden-1-one (**5**)

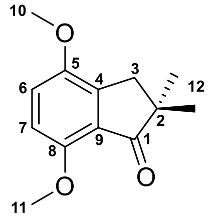

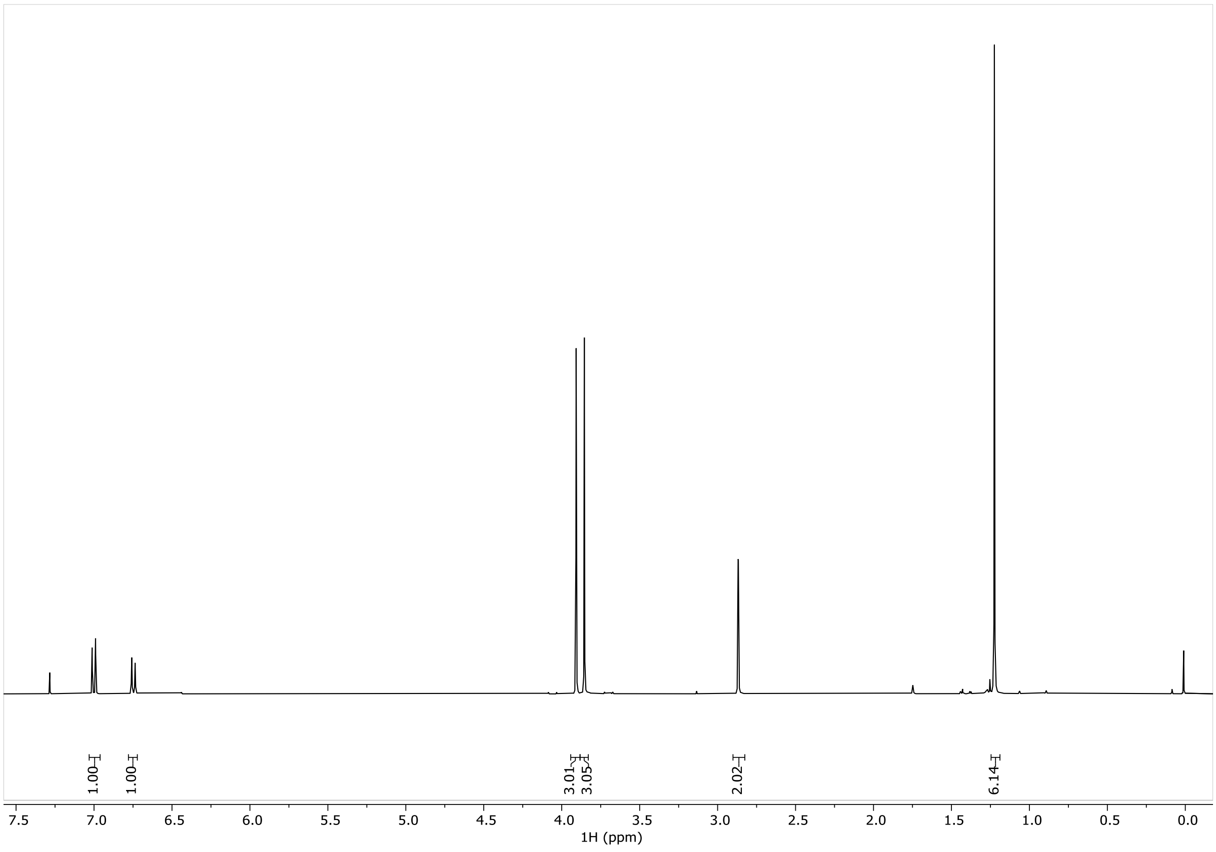

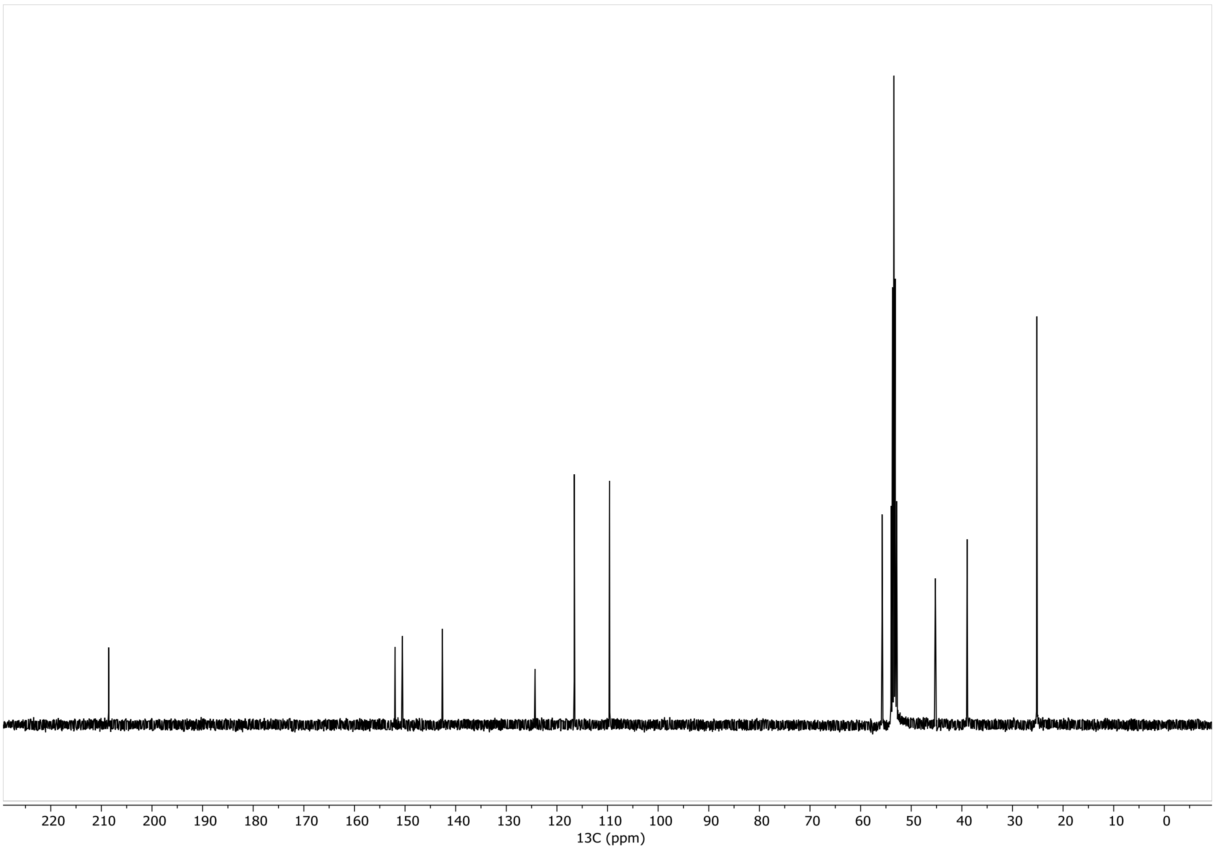
220 210 200 190 180 170 160 150 140 130 120 110 100 90 80 70 60 50 40 30 20 10 0
13C (ppm)

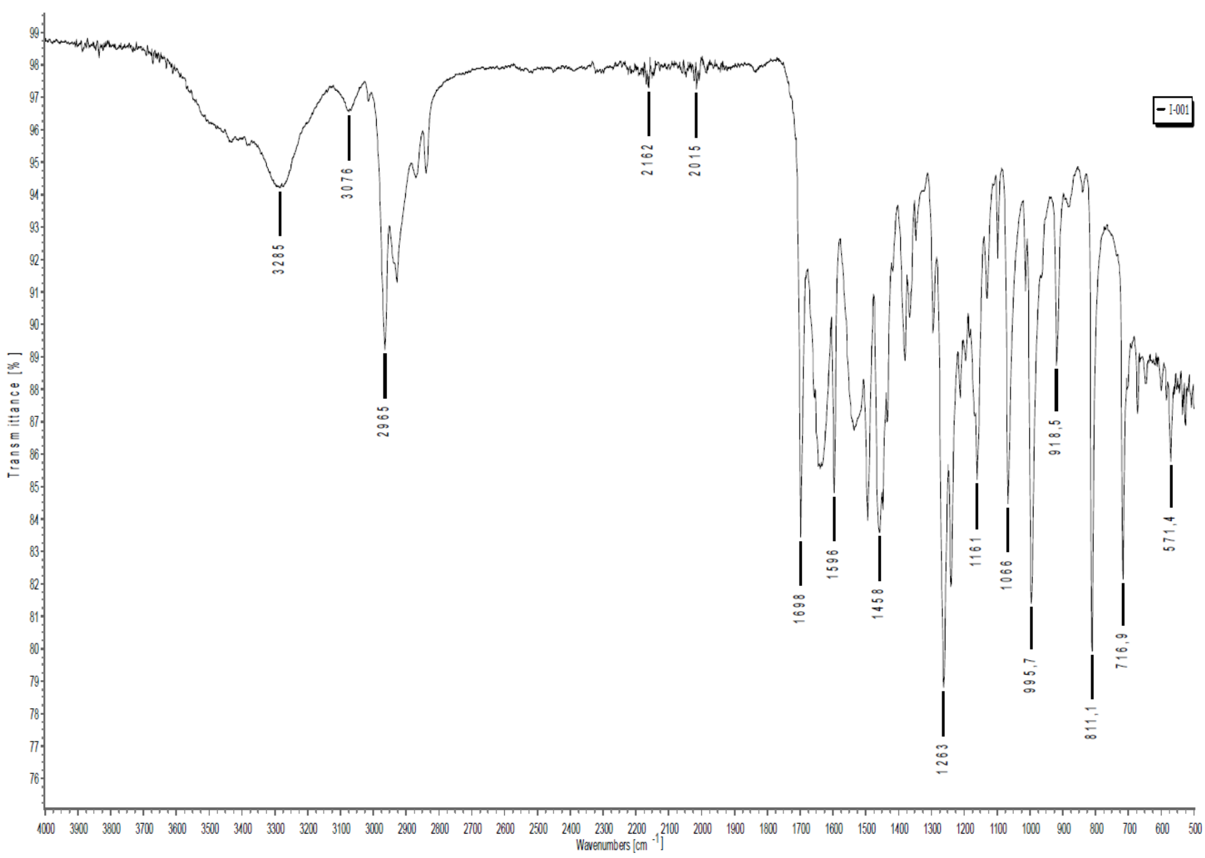
I-001
Transmittance [%]
Wavenumbers [cm -1]
4000 3900 3800 3700 3600 3500 3400 3300 3200 3100 3000 2900 2800 2700 2600 2500 2400 2300 2200 2100 2000 1900 1800 1700 1600 1500 1400 1300 1200 1100 1000 900 800 700 600 500
99 98 97 96 95 94 93 92 91 90 89 88 87 86 85 84 83 82 81 80 79 78 77 76
3285
3076
2965
2162
2015
1698
1596
1458
1263
1161
1066
995,7
918,5
811,1
716,9
571,4

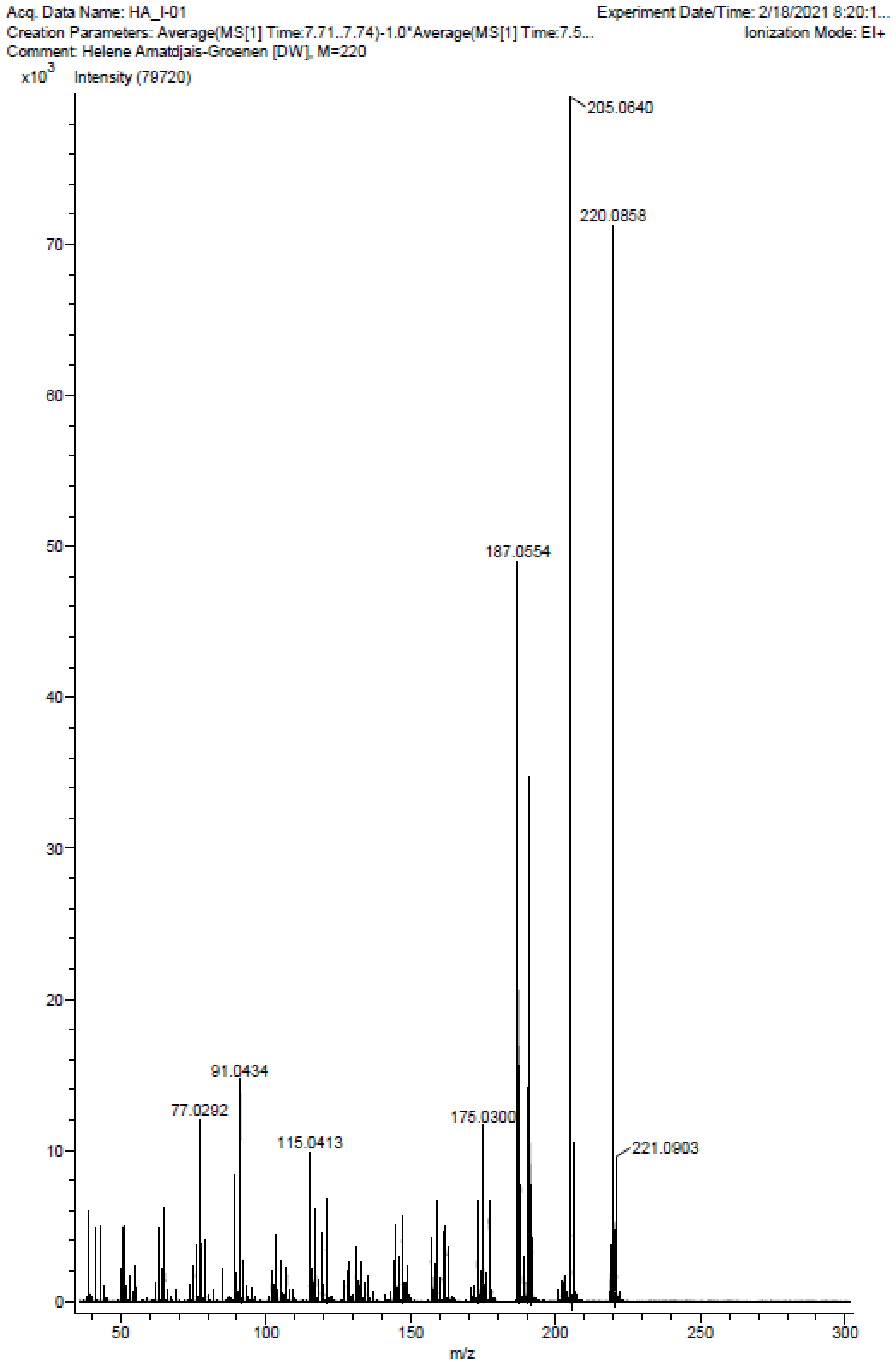
Acq. Data Name: HA_I-01
Experiment Date/Time: 2/18/2021 8:20:1...
Creation Parameters: Average(MS[1] Time:7.71..7.74)-1.0*Average(MS[1] Time:7.5...
Ionization Mode: EI+
Comment: Helene Amatdjais-Groenen [DW], M=220
x10^3 Intensity (79720)
205.0640
220.0858
187.0554
91.0434
77.0292
115.0413
175.0300
221.0903
m/z

(E)/(Z)-4,6-dichloro-2-(4,7-dimethoxy-2,2-dimethyl-2,3-dihydro-1H-inden-lidene)benzo[b]thiophen-3(2H)-one (**6**)

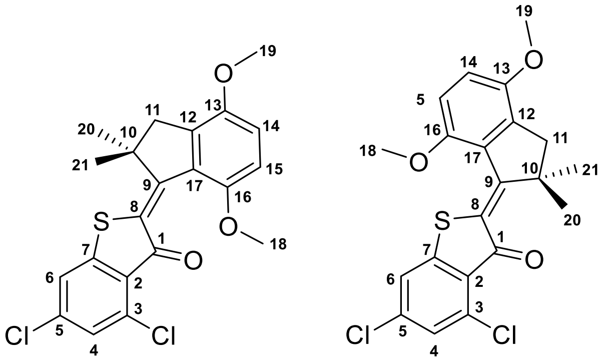

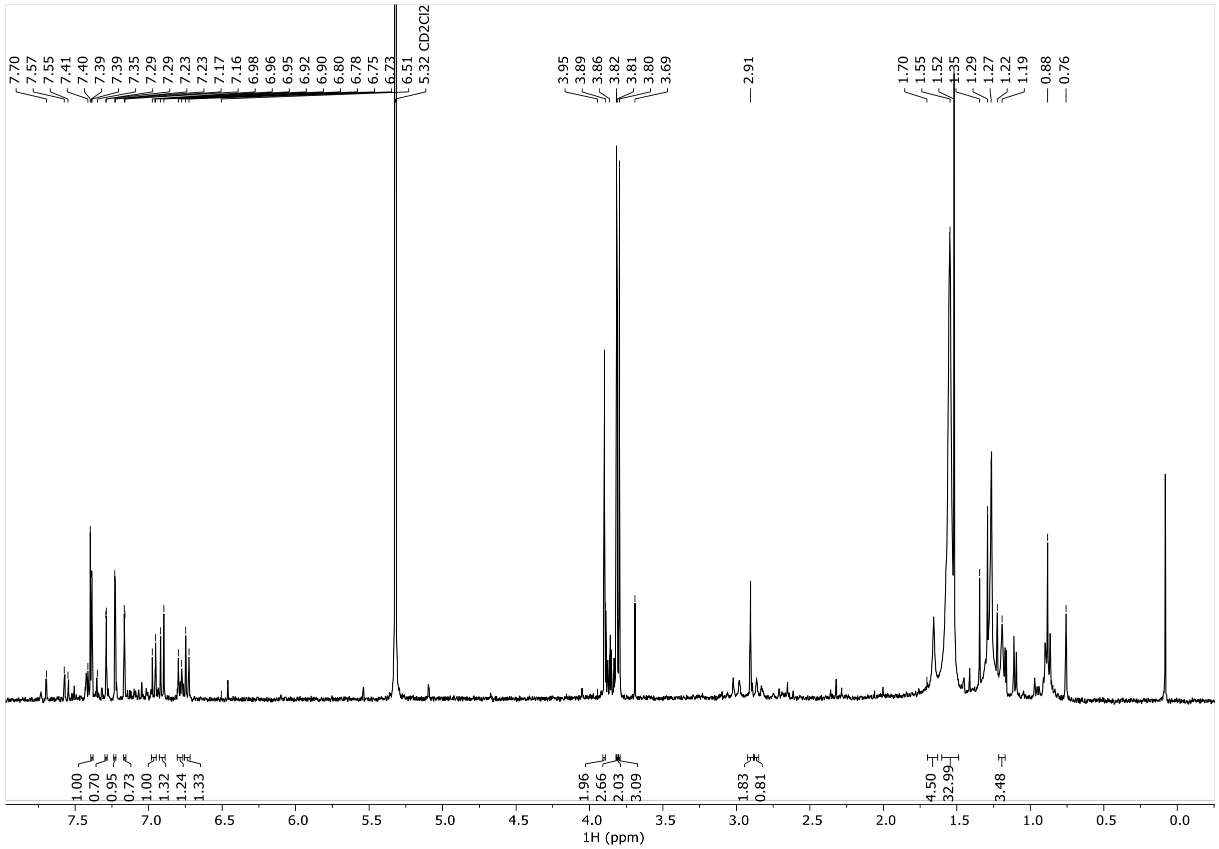

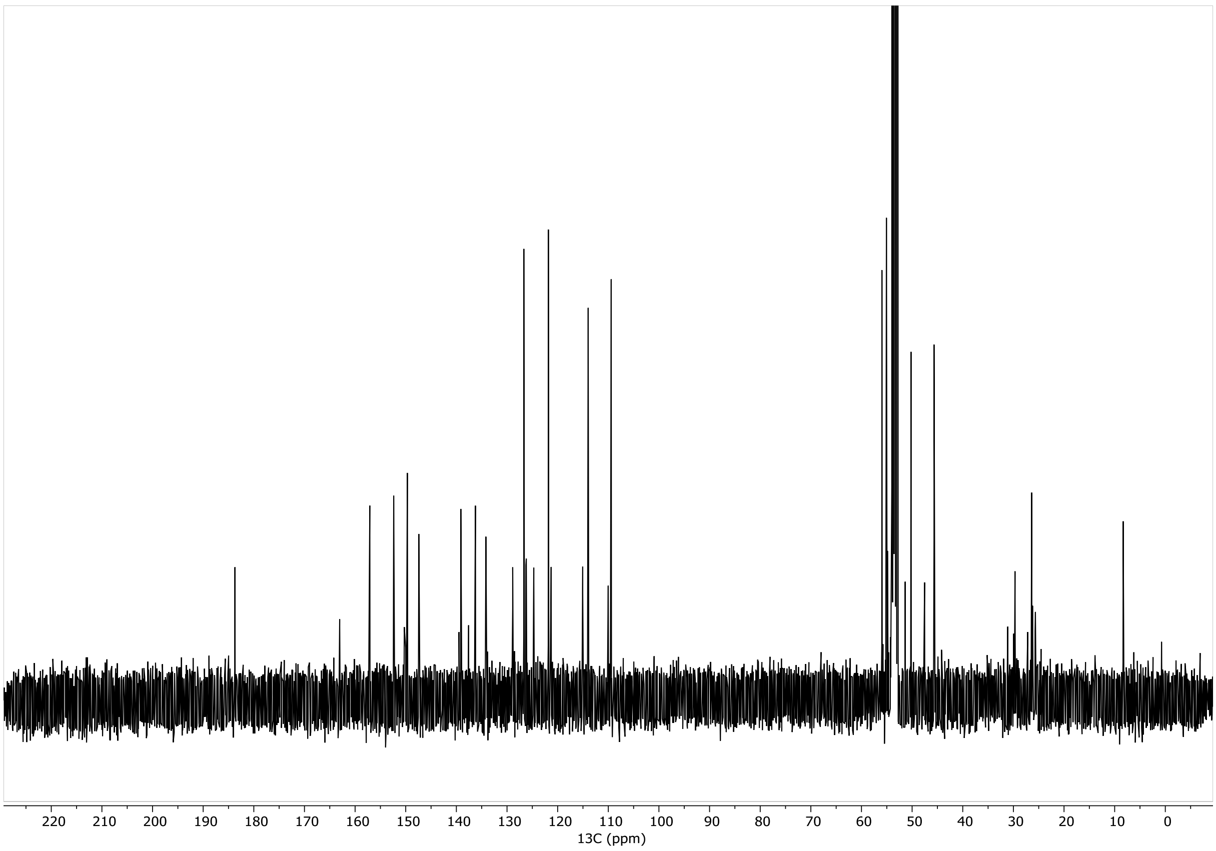
220 210 200 190 180 170 160 150 140 130 120 110 100 90 80 70 60 50 40 30 20 10 0
13C (ppm)

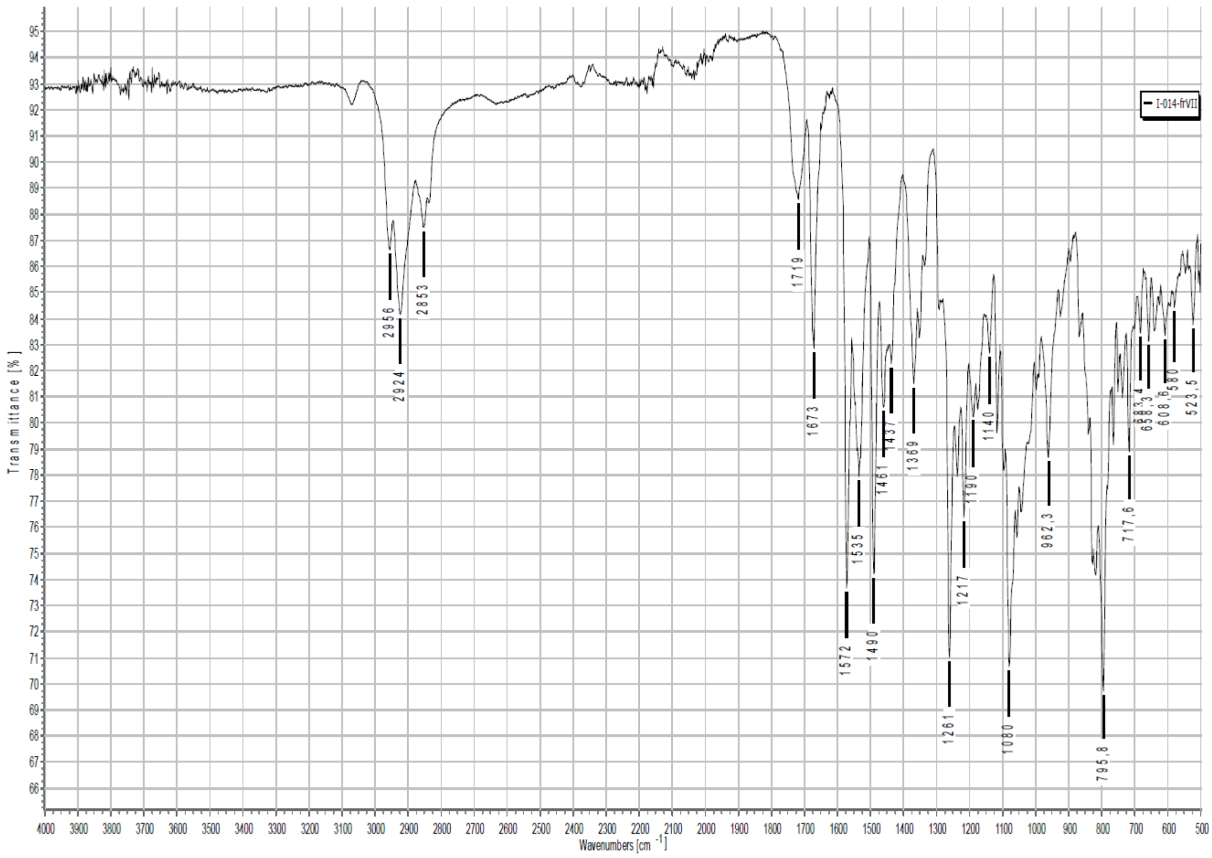
I-014-frVII
Transmittance [%]
Wavenumbers [cm -1]
2956
2924
2853
1719
1673
1572
1535
1490
1461
1437
1369
1261
1217
1190
1140
1080
962,3
795,8
717,6
683,4
658,3
608,6
580
523,5

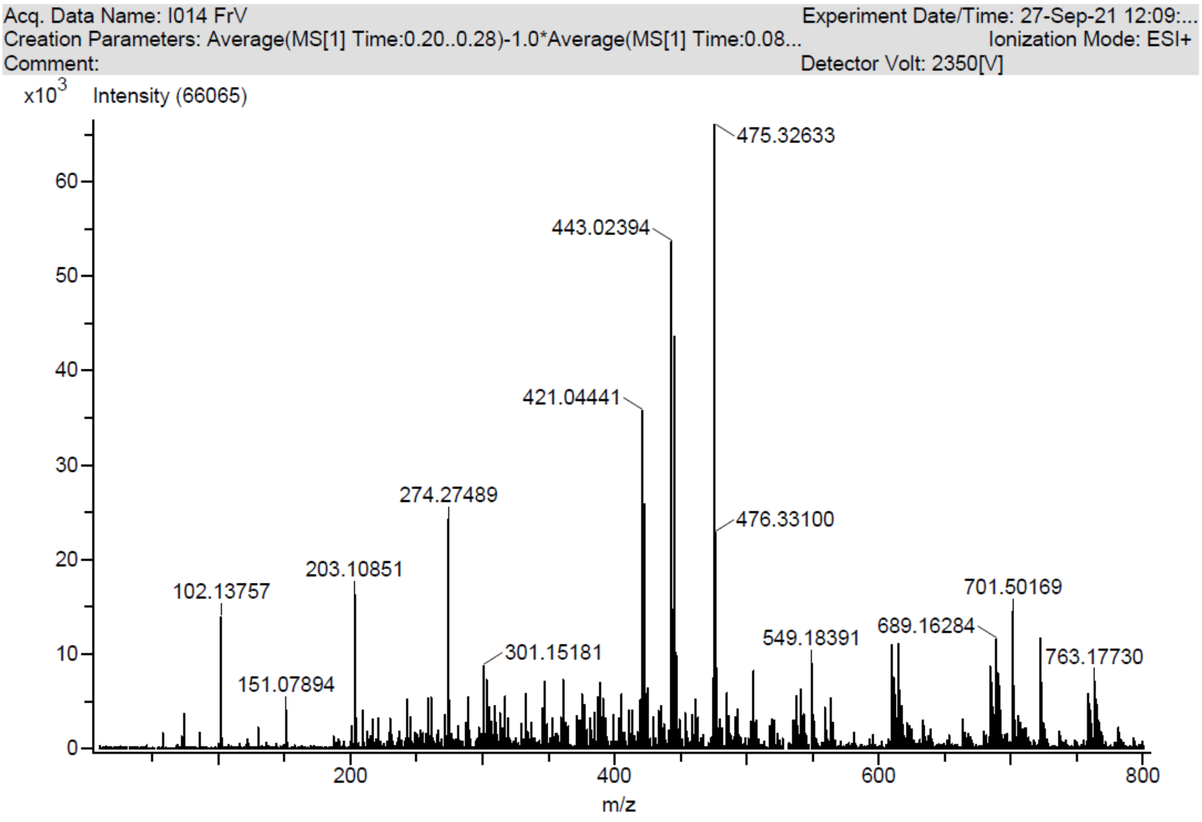
Acq. Data Name: I014 FrV
Experiment Date/Time: 27-Sep-21 12:09:...
Creation Parameters: Average(MS[1] Time:0.20..0.28)-1.0*Average(MS[1] Time:0.08...
Ionization Mode: ESI+
Comment:
Detector Volt: 2350[V]
x10^3 Intensity (66065)
475.32633
443.02394
421.04441
274.27489
476.33100
203.10851
102.13757
701.50169
689.16284
549.18391
301.15181
763.17730
151.07894
m/z

(E)/(Z)-4,6-dichloro-2-(4,7-dimethoxy-2,2-dimethyl-2,3-dihydro-1H-inden-1-ylidene)benzo[b]thiophen-3(2H)-one 1-oxide (**7**)

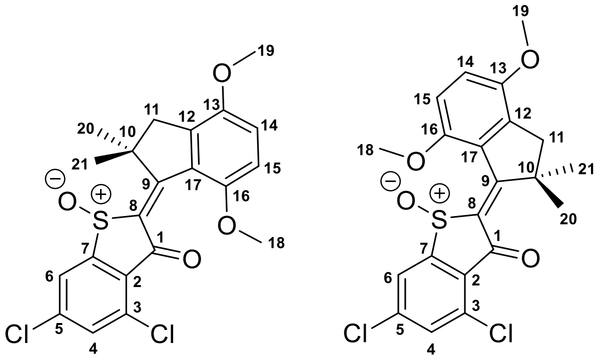

E:

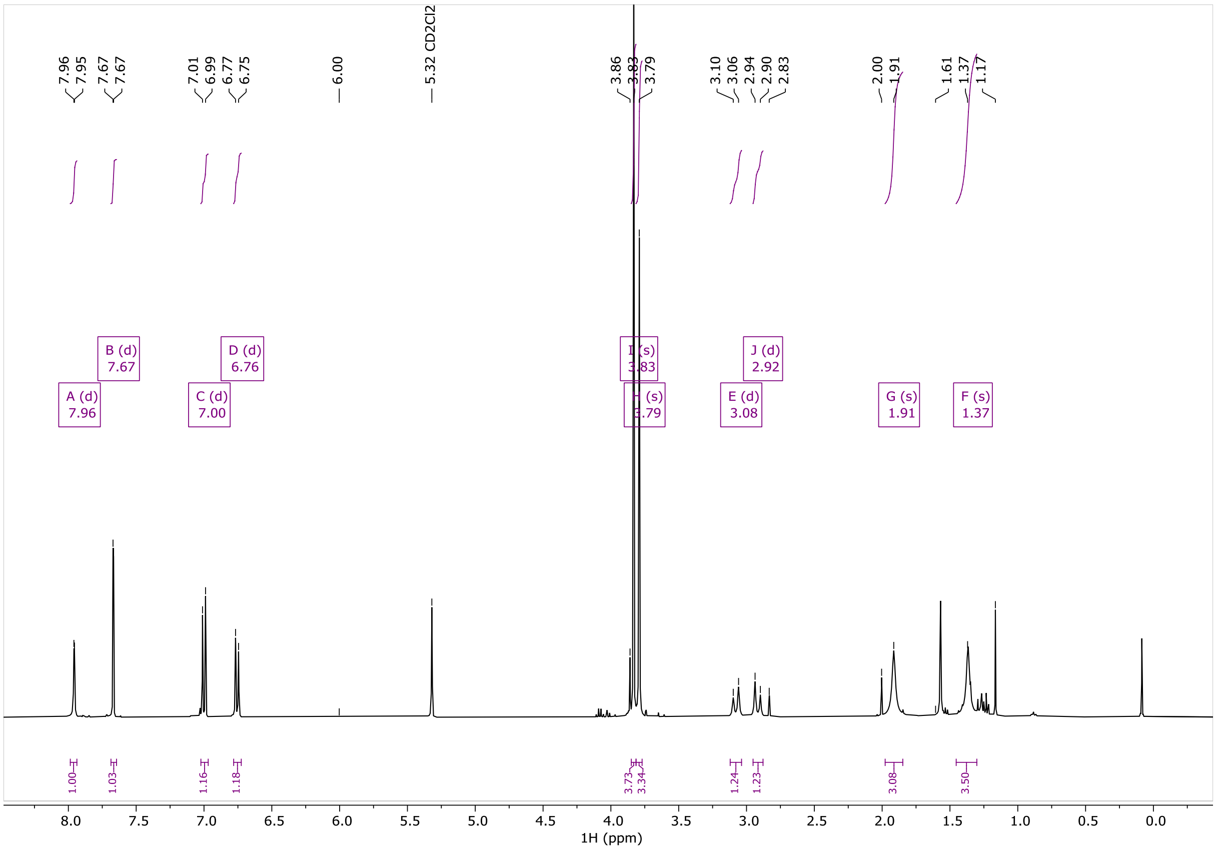

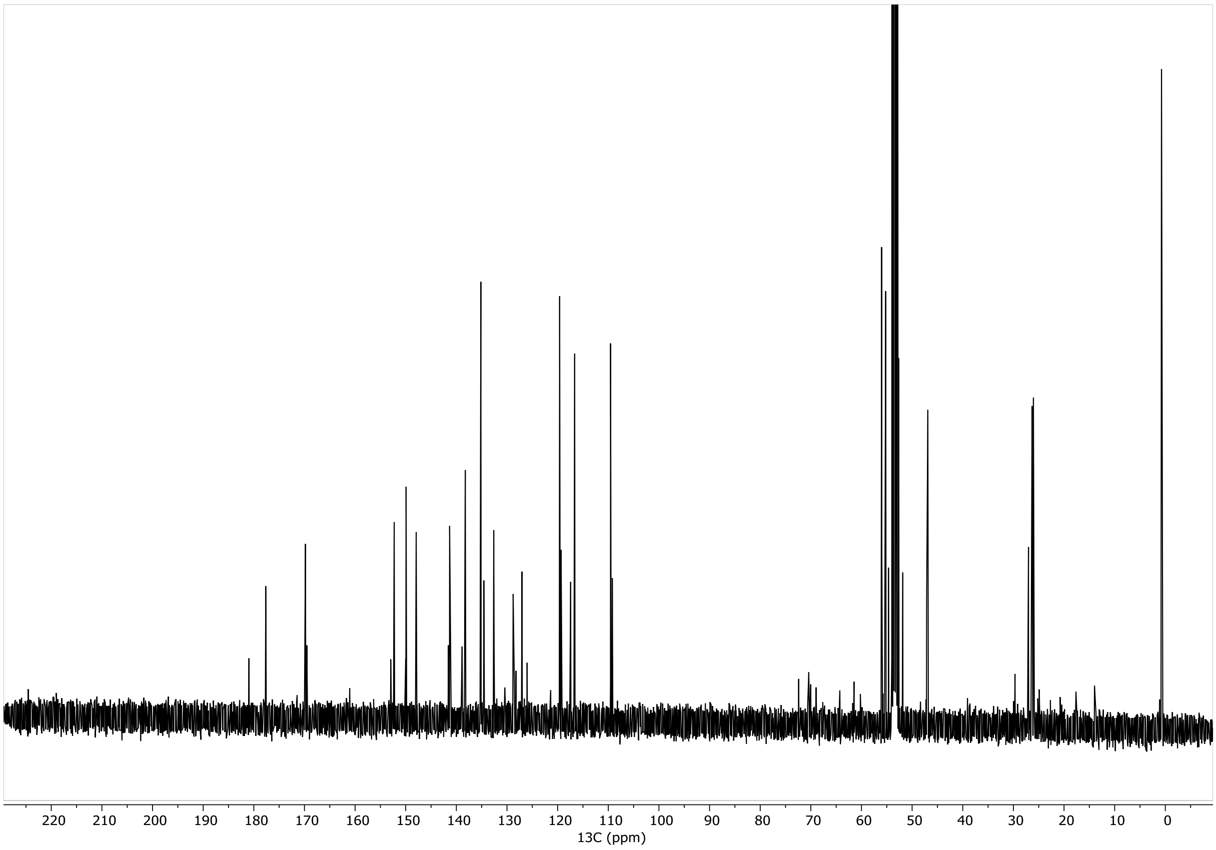

220 210 200 190 180 170 160 150 140 130 120 110 100 90 80 70 60 50 40 30 20 10 0
13C (ppm)

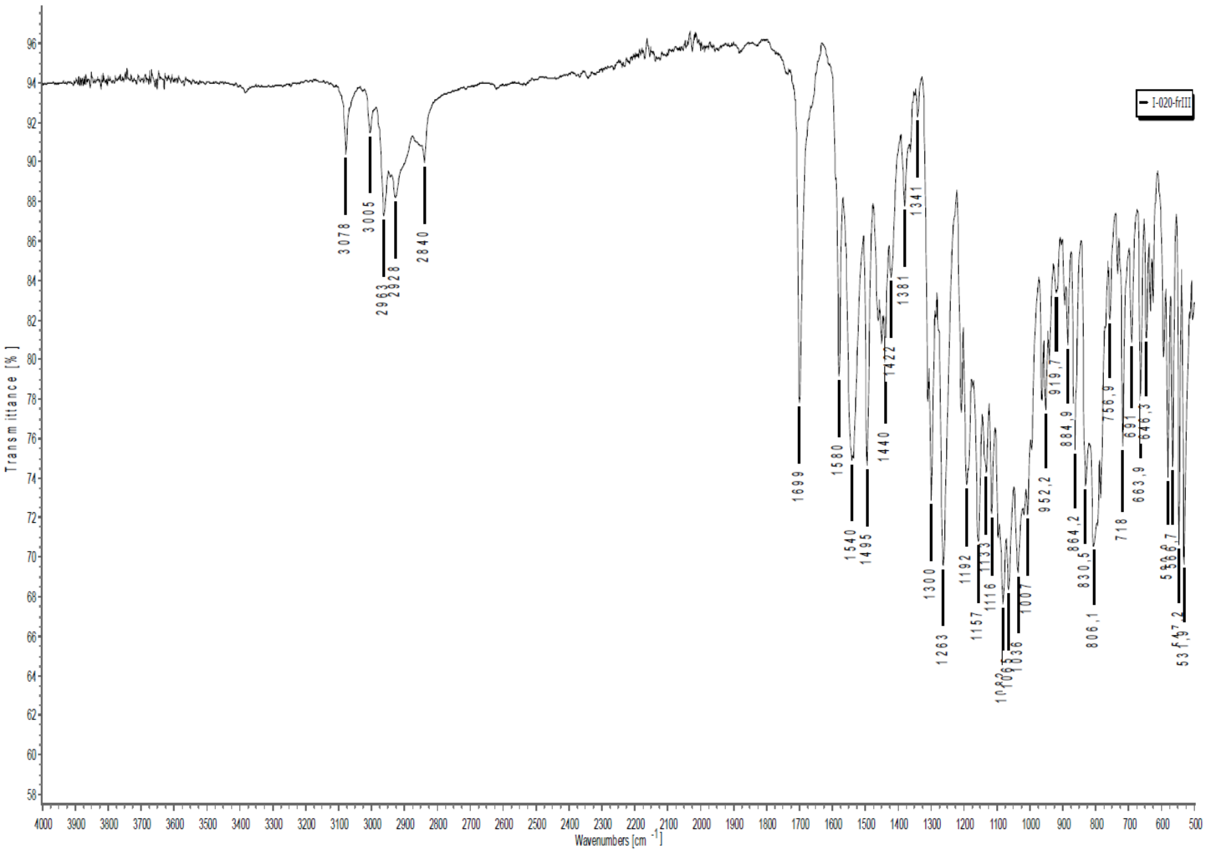

I-020-frIII
Transmittance [%]
Wavenumbers [cm -1]
3078
3005
2963
2928
2840
1699
1580
1540
1495
1440
1422
1381
1341
1300
1263
1192
1157
1133
1116
1036
1007
952,2
919,7
884,9
864,2
830,5
806,1
756,9
718
691
663,9
646,3
547,2
531,9

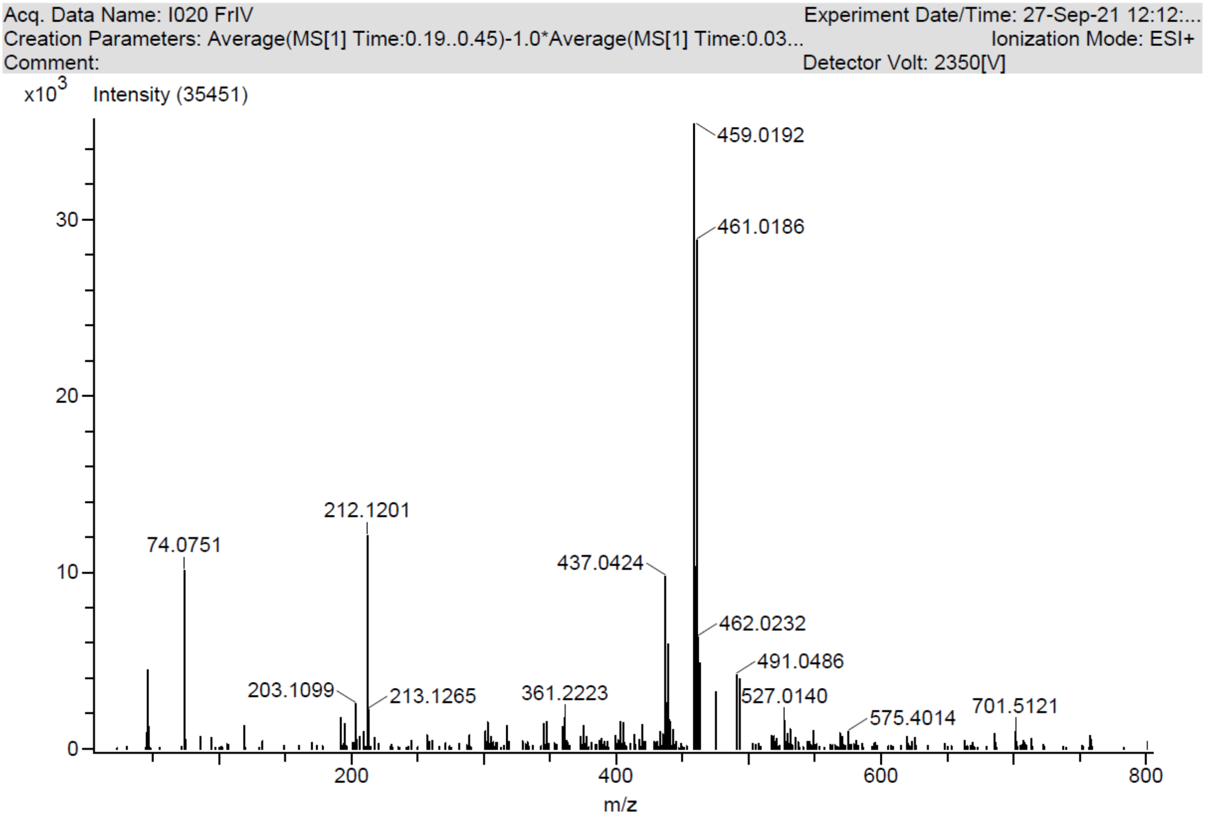

Acq. Data Name: I020 FrIV
Experiment Date/Time: 27-Sep-21 12:12:...
Creation Parameters: Average(MS[1] Time:0.19..0.45)-1.0*Average(MS[1] Time:0.03...
Ionization Mode: ESI+
Comment:
Detector Volt: 2350[V]
x10^3 Intensity (35451)
459.0192
461.0186
212.1201
74.0751
437.0424
462.0232
491.0486
203.1099
213.1265
361.2223
527.0140
575.4014
701.5121
m/z

Z:

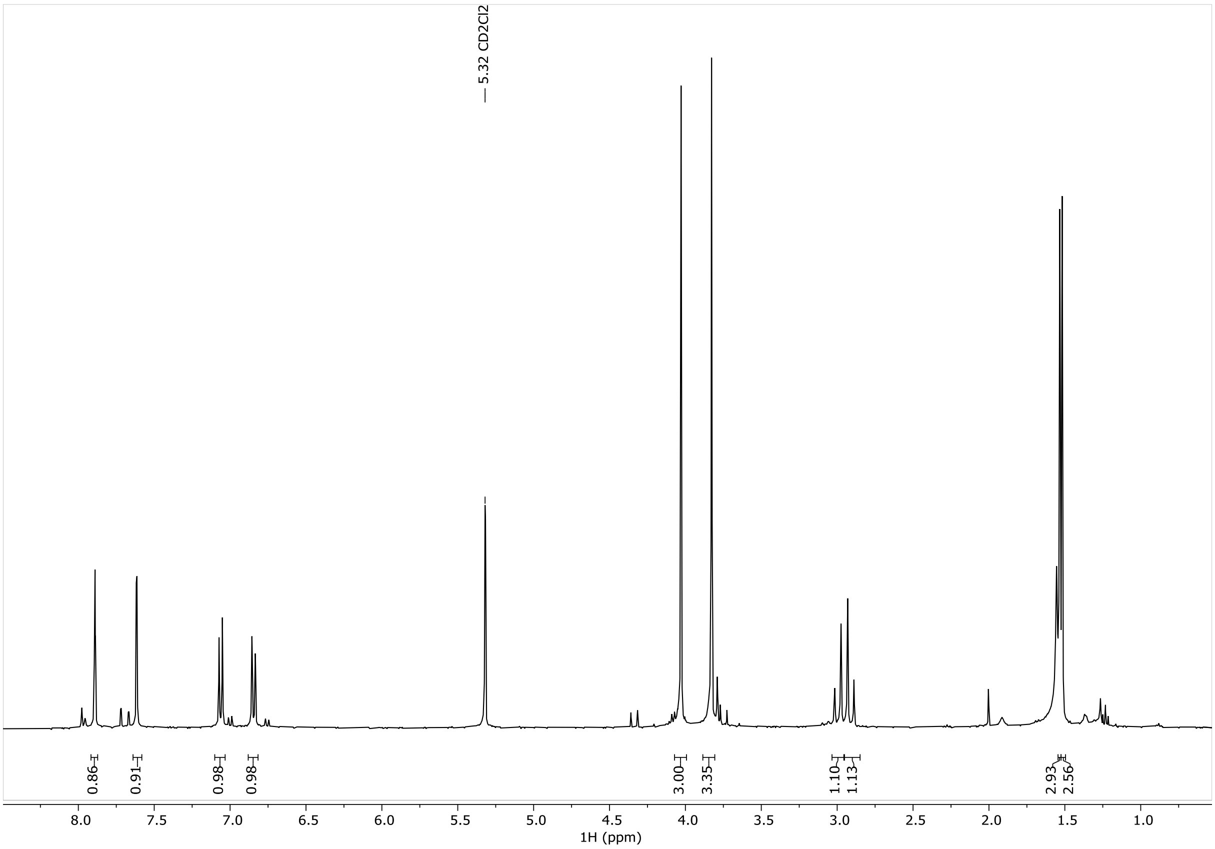

5.32 CD2Cl2
0.86
0.91
0.98
0.98
3.00
3.35
1.10
1.13
2.93
2.56
1H (ppm)

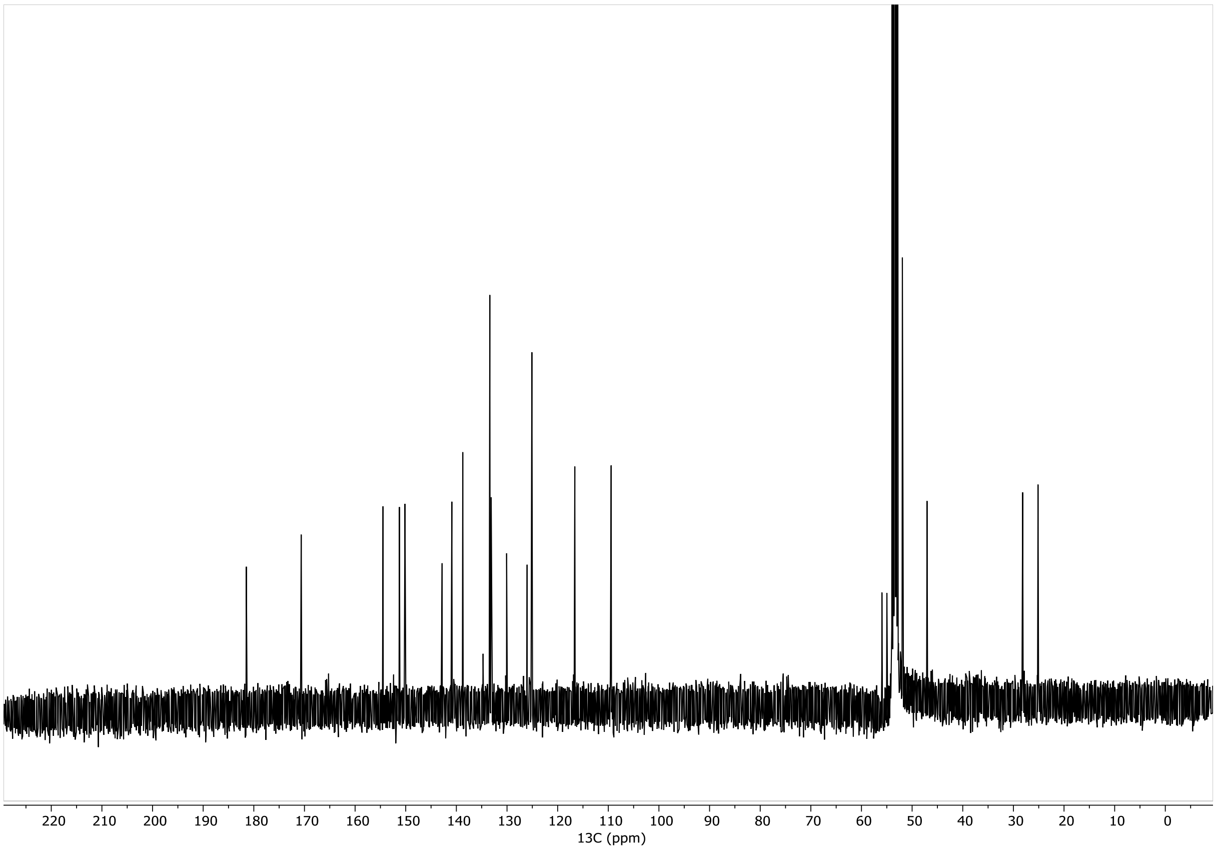
220 210 200 190 180 170 160 150 140 130 120 110 100 90 80 70 60 50 40 30 20 10 0
13C (ppm)

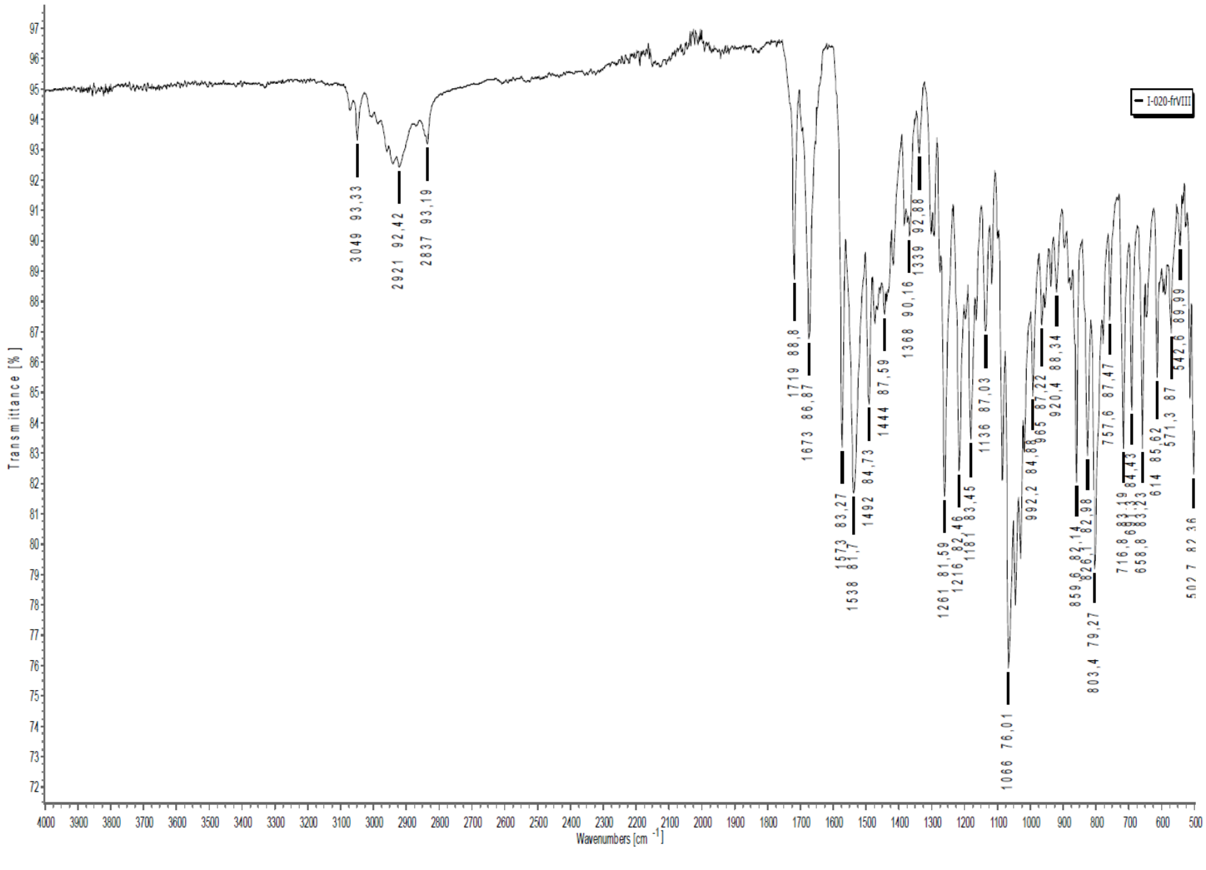
Transmittance [%]
Wavenumbers [cm -1]
I-020-frVIII
3049 93,33
2921 92,42
2837 93,19
1719 88,8
1673 86,87
1573 83,27
1538 81,7
1492 84,73
1444 87,59
1368 90,16
1339 92,88
1261 81,59
1216 82,46
1181 83,45
1136 87,03
1066 76,01
992,2 84,88
965 87,22
920,4 88,34
859,6 82,14
826,1 82,98
803,4 79,27
757,6 87,47
716,8 83,19
658,8 83,23
614 85,62
571,3 87
542,6 89,99

Acq. Data Name: I-020-frVIII Experiment Date/Time: 08-Feb-22 10:58:...
Creation Parameters: Average(MS[1] Time:0.20..0.29)-1.0*Average(MS[1] Time:0.07... Ionization Mode: ESI+
Comment: C21H18Cl2O4S Detector Volt: 2400[V]

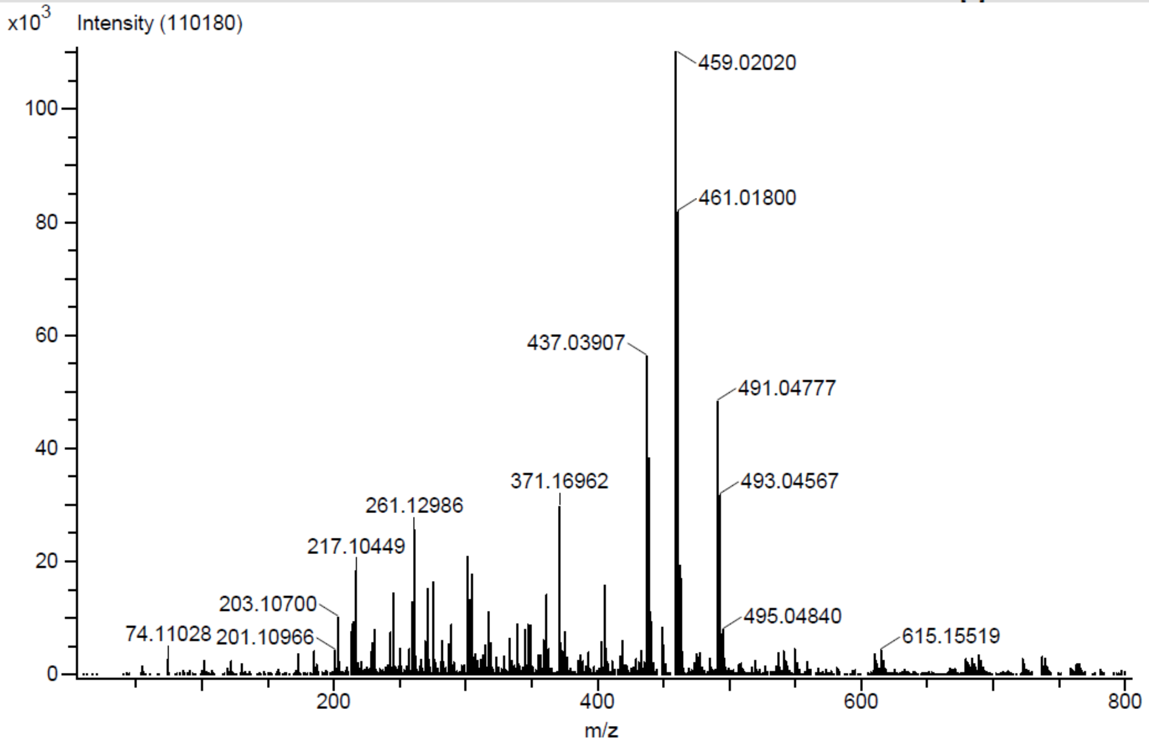

2-(4-(azidomethyl)phenyl)-4,4,5,5-tetramethyl-1,3,2-Dioxaborolane (**9**)

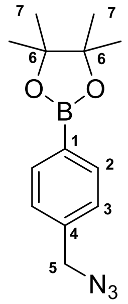

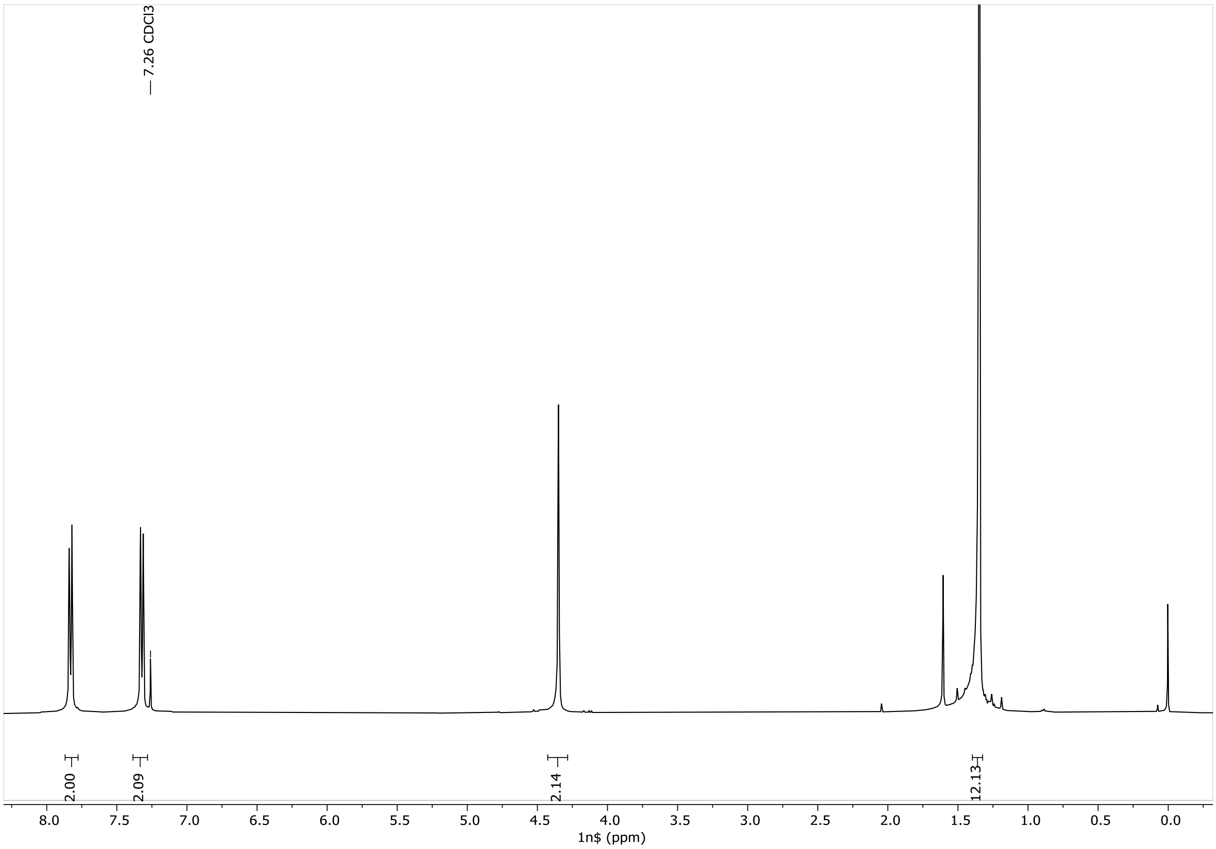

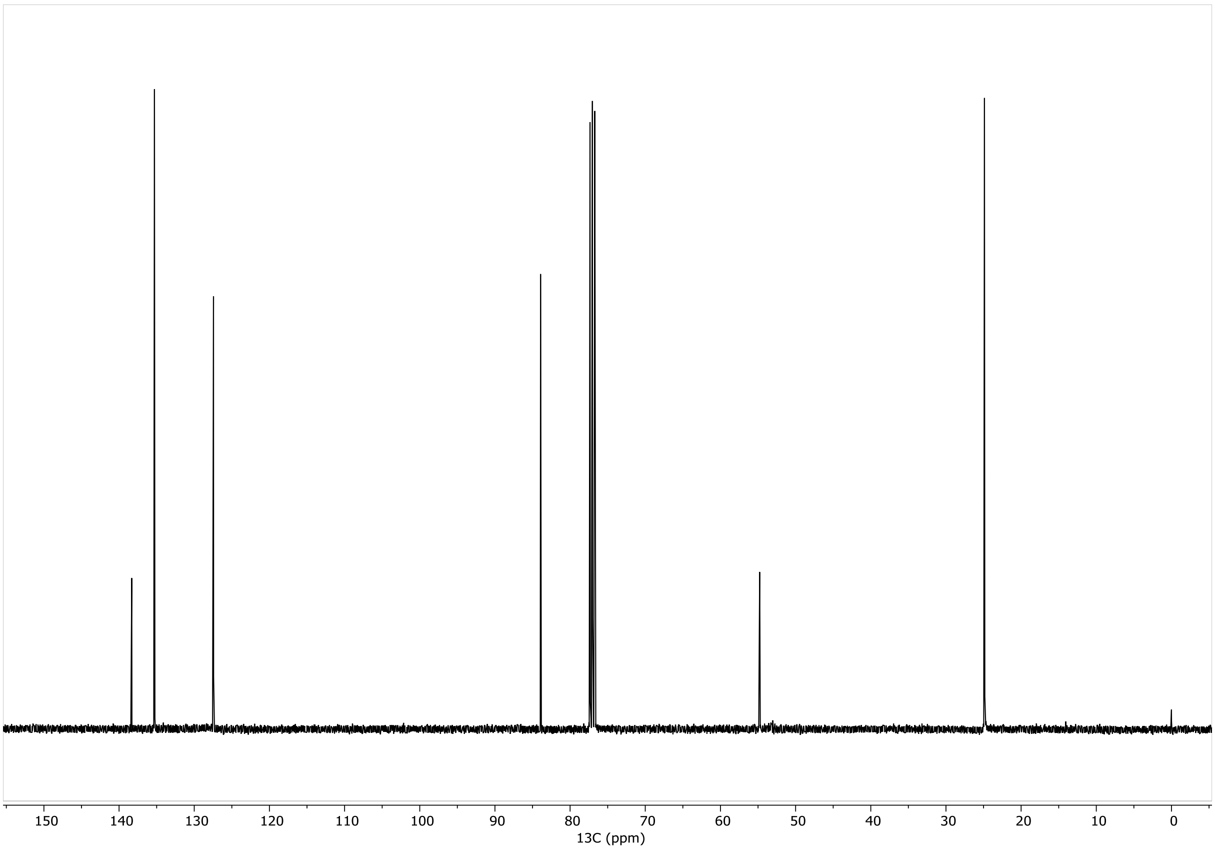
150
140
130
120
110
100
90
80
70
60
50
40
30
20
10
0
13C (ppm)

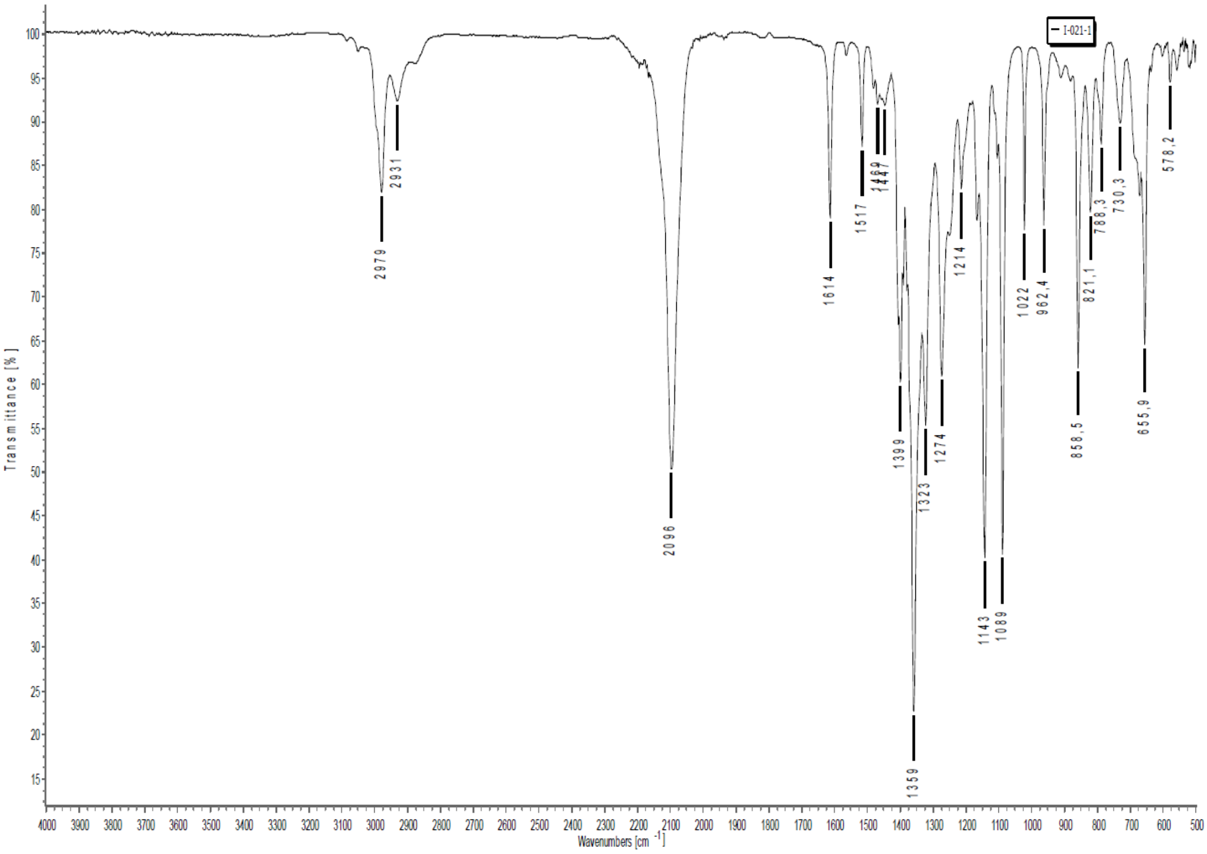
I-021-1
Transmittance [%]
Wavenumbers [cm -1]
2979
2931
2096
1614
1517
1447
1399
1359
1323
1274
1214
1143
1089
1022
962,4
858,5
821,1
788,3
730,3
655,9
578,2

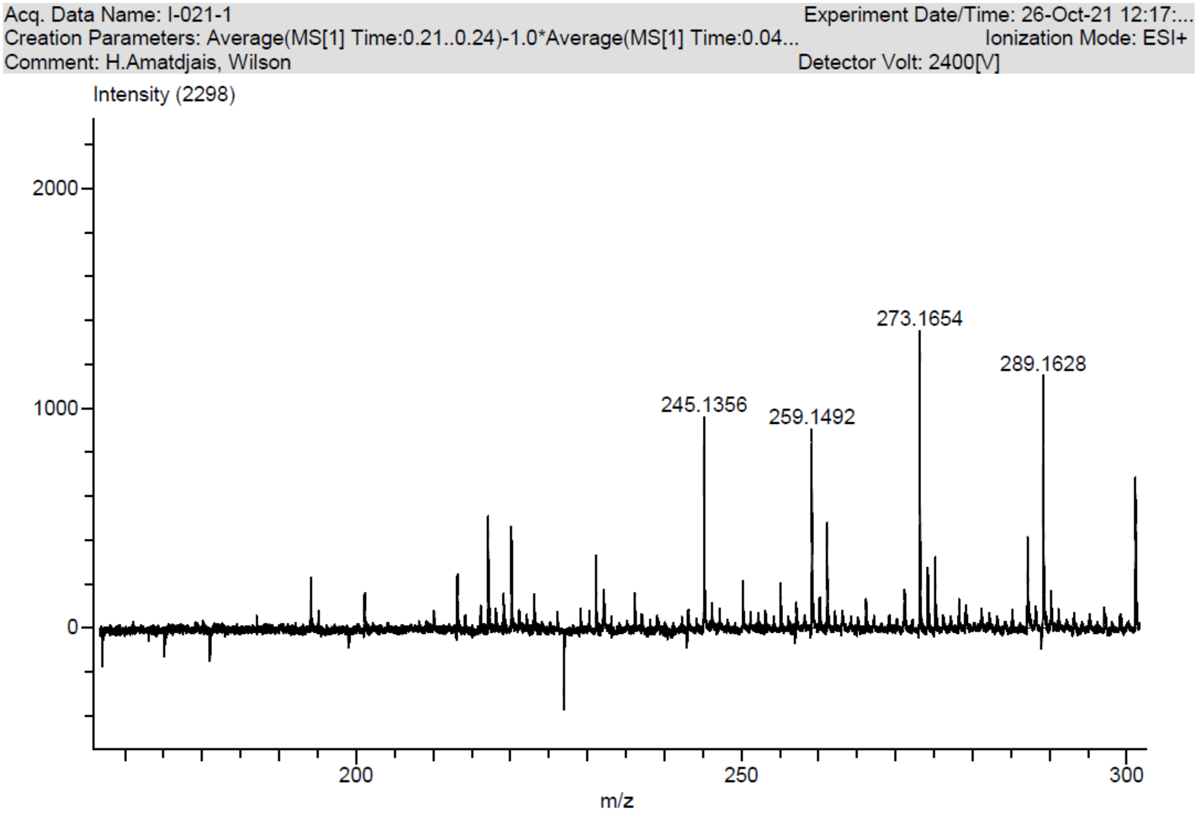
Acq. Data Name: I-021-1
Experiment Date/Time: 26-Oct-21 12:17:...
Creation Parameters: Average(MS[1] Time:0.21..0.24)-1.0*Average(MS[1] Time:0.04...
Ionization Mode: ESI+
Comment: H.Amatdjais, Wilson
Detector Volt: 2400[V]
Intensity (2298)
2000
1000
0
273.1654
289.1628
245.1356
259.1492
200
250
300
m/z

(Z)-4-(4-(azidomethyl)phenyl)-6-chloro-2-(4,7-dimethoxy-2,2-dimethyl-2,3-dihydro-1H-inden-1-ylidene)benzo[b]thiophen-3(2H)-one 1-oxide (**10a**) and (Z)-6-(4-(azidomethyl)phenyl)-4-chloro-2-(4,7-dimethoxy-2,2-dimethyl-2,3-dihydro-1H-inden-1-ylidene)benzo[b]thiophen-3(2H)-one 1-oxide (**10b**)

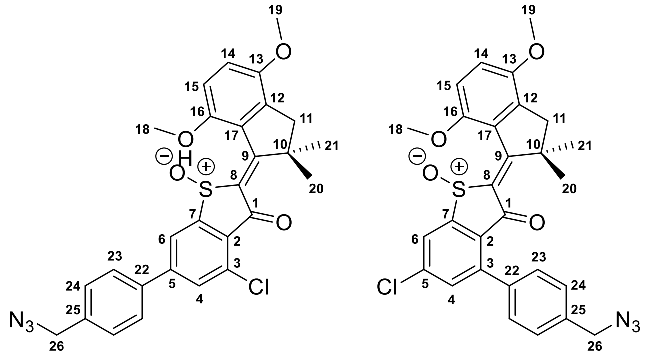

10a:

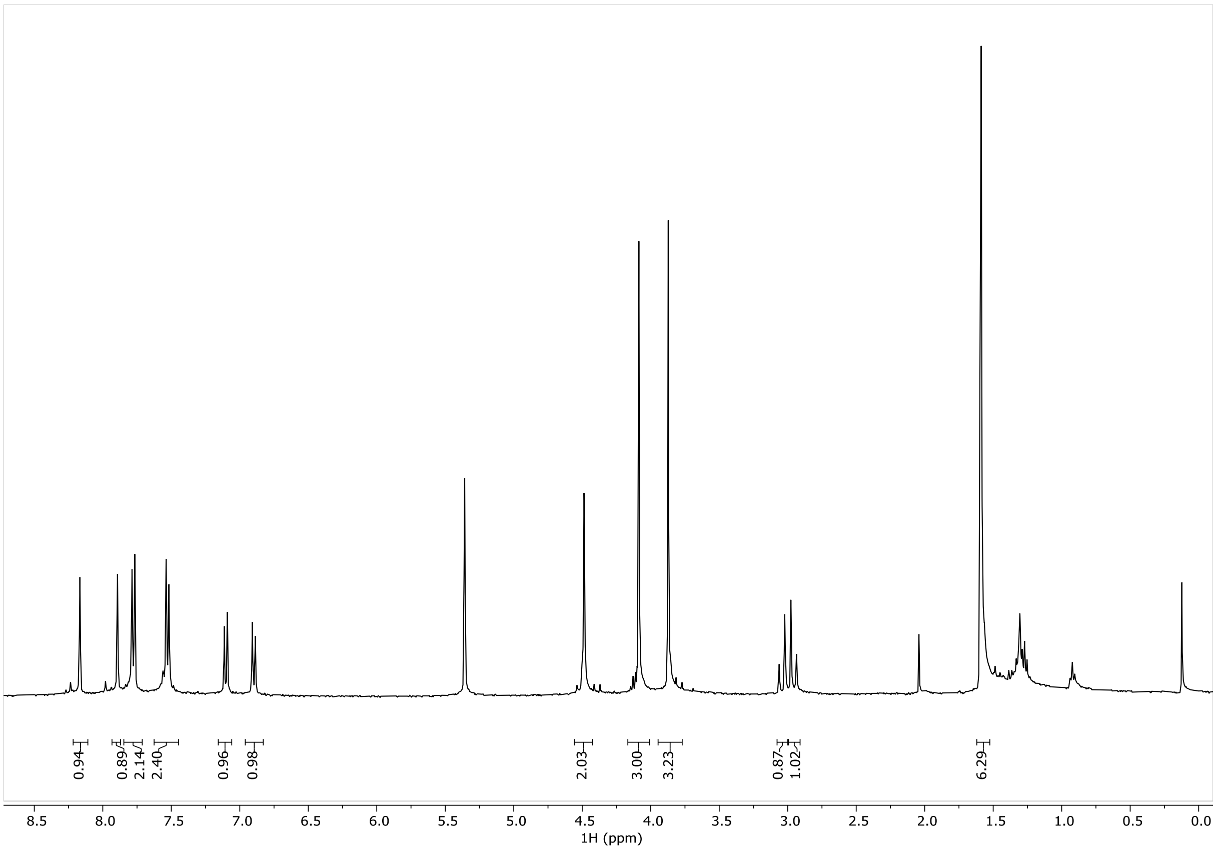

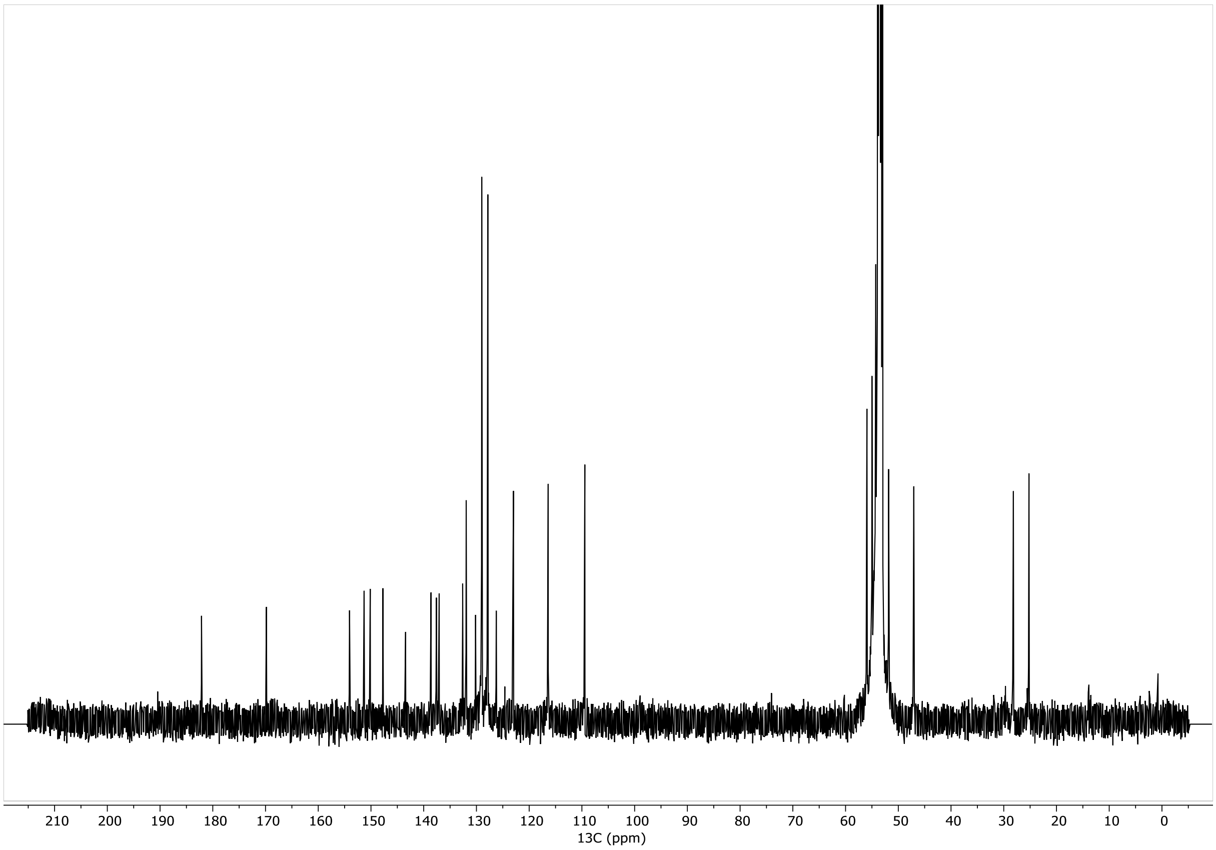
210 200 190 180 170 160 150 140 130 120 110 100 90 80 70 60 50 40 30 20 10 0
13C (ppm)

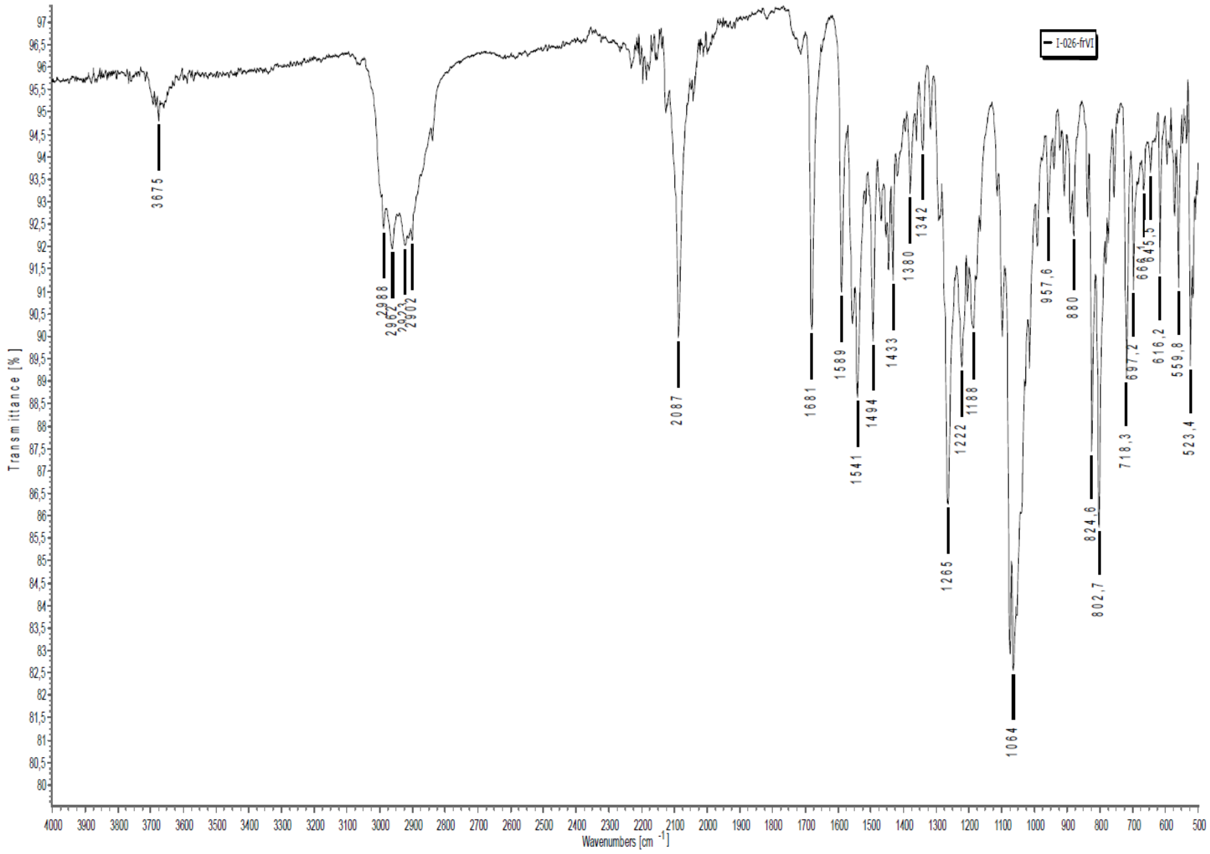
I-026-frVI
Transmittance [%]
Wavenumbers [cm -1]
3675
2988
2962
2902
2087
1681
1589
1541
1494
1433
1380
1342
1265
1222
1188
1064
957,6
880
824,6
802,7
718,3
697,2
645,5
616,2
559,8
523,4

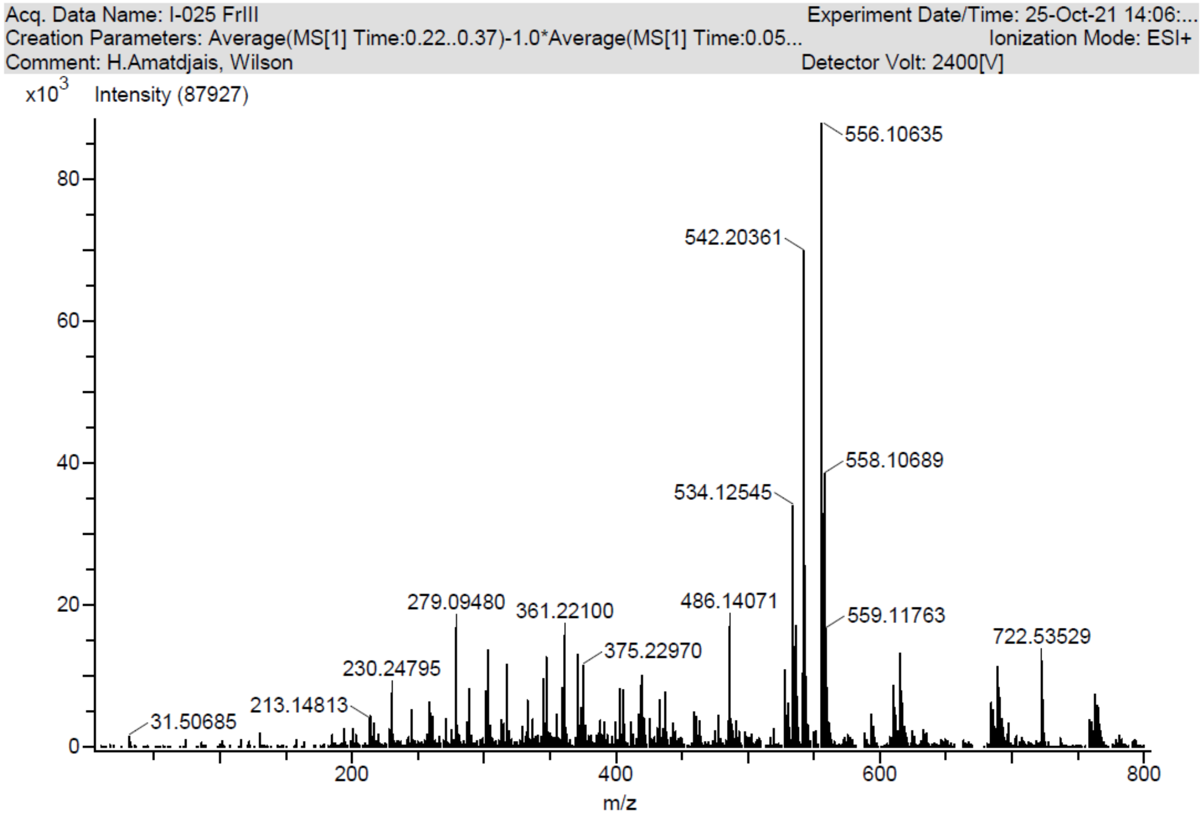
Acq. Data Name: I-025 FrIII
Experiment Date/Time: 25-Oct-21 14:06:...
Creation Parameters: Average(MS[1] Time:0.22..0.37)-1.0*Average(MS[1] Time:0.05...
Ionization Mode: ESI+
Comment: H.Amatdjais, Wilson
Detector Volt: 2400[V]
x10^3 Intensity (87927)
556.10635
542.20361
558.10689
534.12545
279.09480
361.22100
486.14071
559.11763
722.53529
375.22970
230.24795
213.14813
31.50685
m/z

10b:

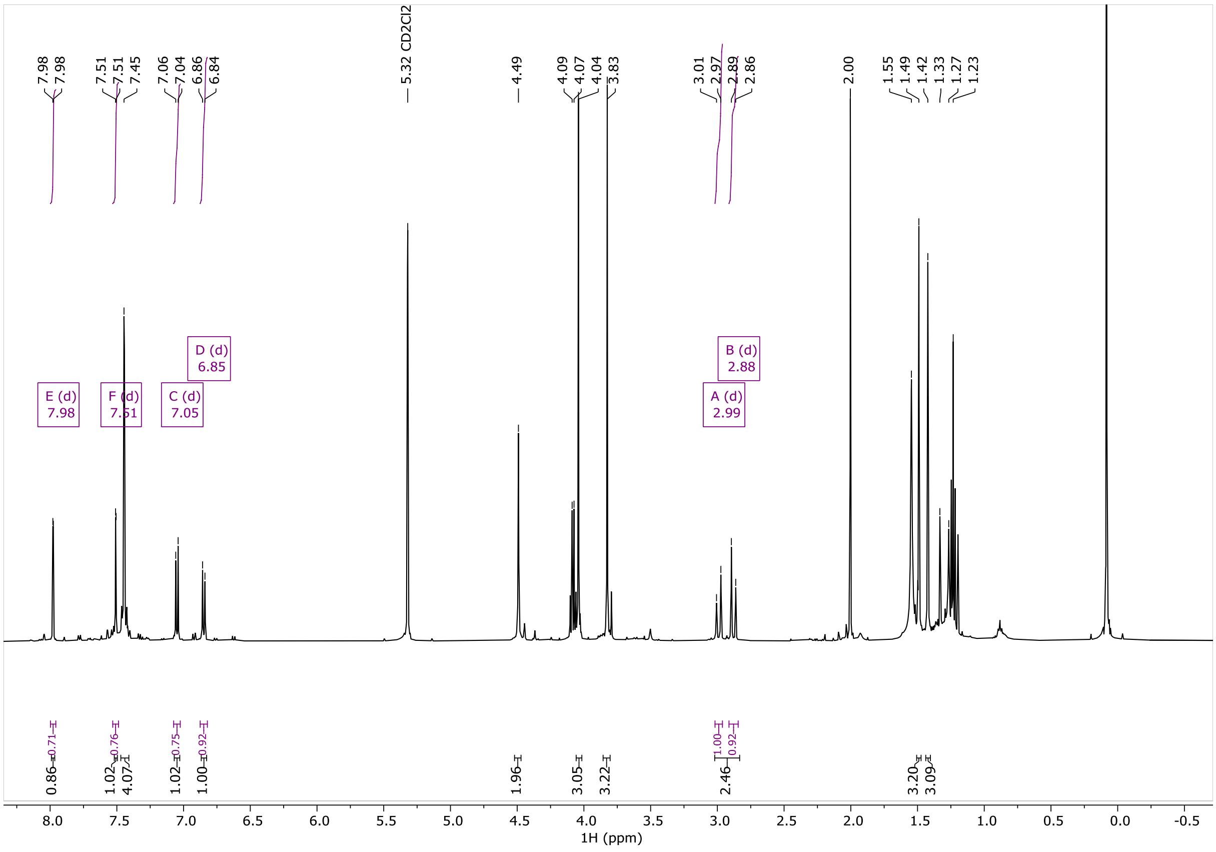
5.32 CD2Cl2
E (d)
7.98
F (d)
7.51
C (d)
7.05
D (d)
6.85
B (d)
2.88
A (d)
2.99
1H (ppm)

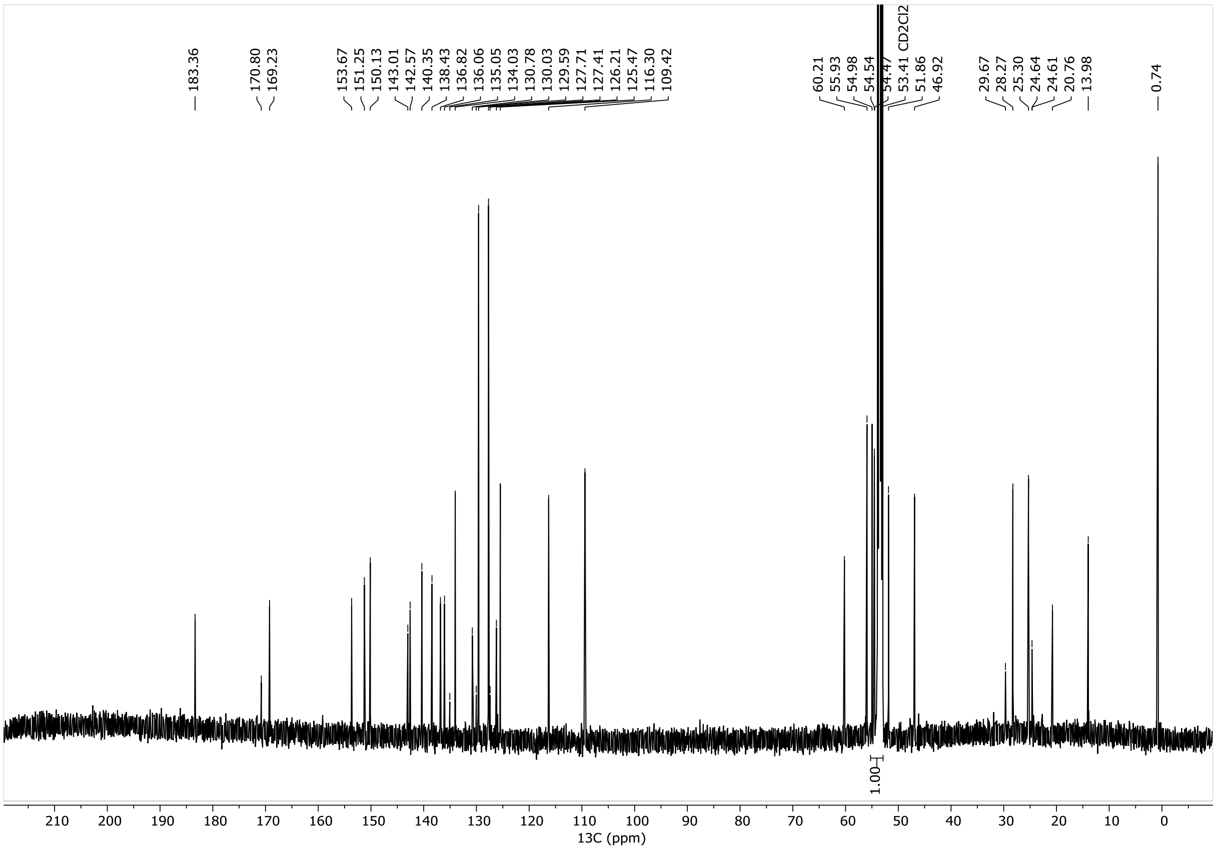
183.36
170.80
169.23
153.67
151.25
150.13
143.01
142.57
140.35
138.43
136.82
136.06
135.05
134.03
130.78
130.03
129.59
127.71
127.41
126.21
125.47
116.30
109.42
60.21
55.93
54.98
54.54
54.47
53.41 CD2Cl2
51.86
46.92
29.67
28.27
25.30
24.64
24.61
20.76
13.98
0.74
1.00
13C (ppm)

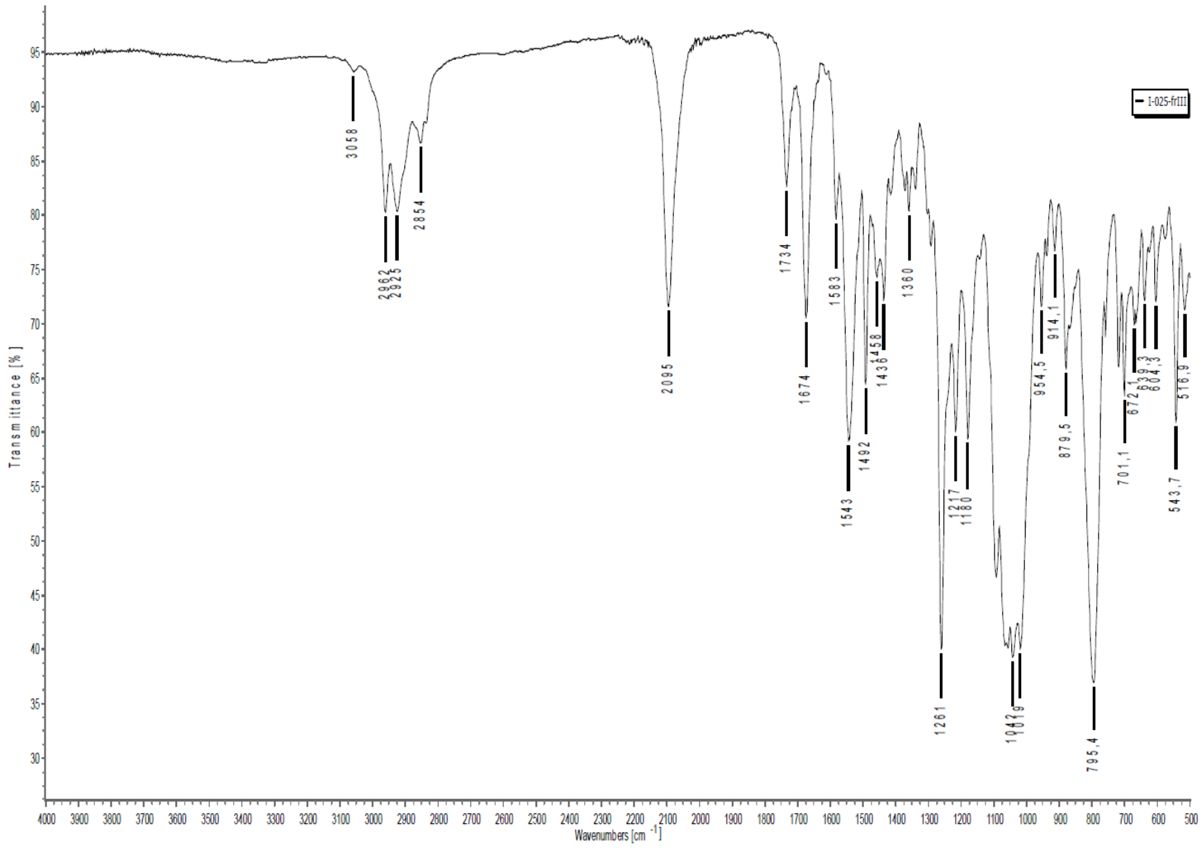
I-025-frIII
Transmittance [%]
Wavenumbers [cm -1]
3058
2962
2925
2854
2095
1734
1674
1583
1543
1492
1458
1436
1360
1261
1217
1180
1042
1019
954,5
914,1
879,5
795,4
701,1
672,1
639,3
604,3
543,7
516,9

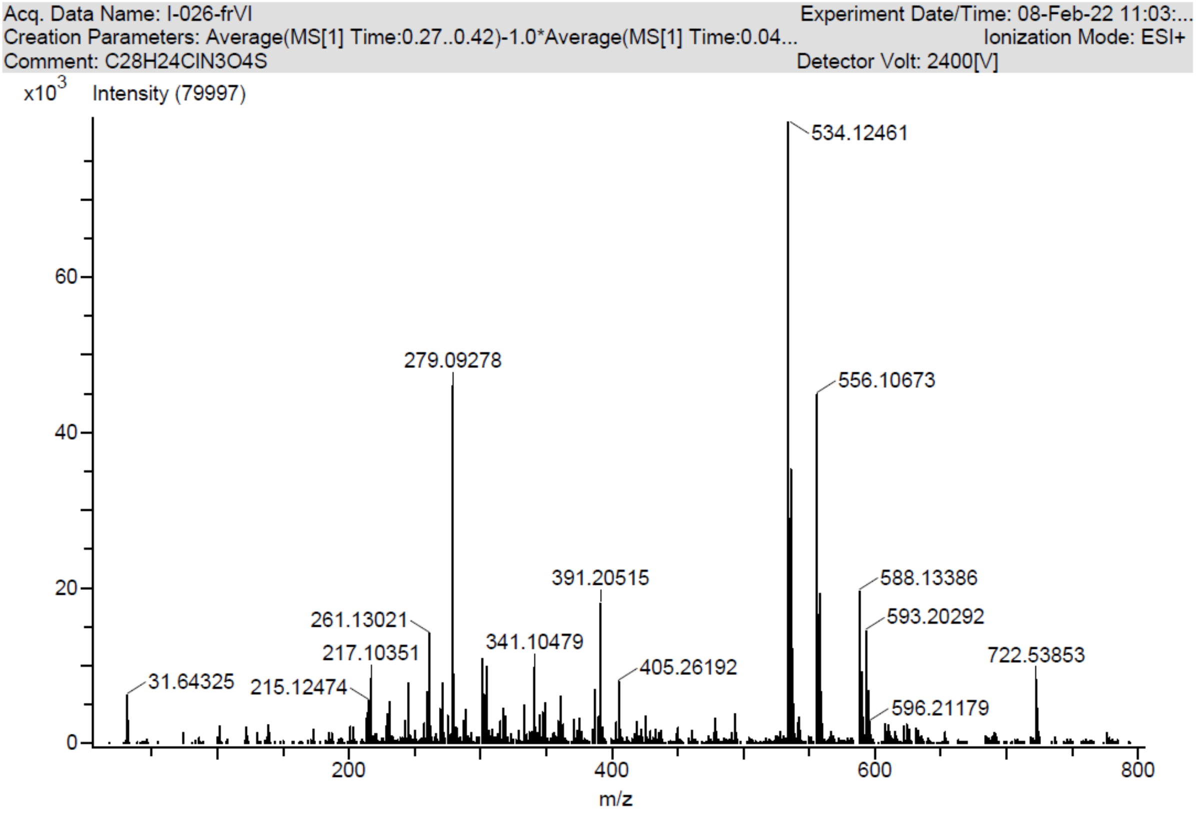
Acq. Data Name: I-026-frVI
Experiment Date/Time: 08-Feb-22 11:03:...
Creation Parameters: Average(MS[1] Time:0.27..0.42)-1.0*Average(MS[1] Time:0.04...
Ionization Mode: ESI+
Comment: C28H24ClN3O4S
Detector Volt: 2400[V]
x10^3 Intensity (79997)
534.12461
556.10673
279.09278
391.20515
588.13386
593.20292
261.13021
341.10479
217.10351
722.53853
405.26192
31.64325
215.12474
596.21179
0
20
40
60
200
400
600
800
m/z
